# Antidiabetic and Antioxidant Potential of a New Bisglyceride Derivative Together with Other Compounds from the Root Bark of *Pithecellobium dulce*: In Vitro and In Silico Studies

**DOI:** 10.3390/molecules31122166

**Published:** 2026-06-19

**Authors:** Gertrude Nembot Messah, Peron Bosco Leutcha, Gabrielle Ange Amang à Ngnoung, Guy Roussel Takuissu Nguemto, Brice Junior Edie Enang, Hamadou Mamoudou, Soh Désiré, William Feudjou Fouatio, Alembert Tiabou Tchinda, Bienvenu Tsakem, Madan Poka, Patrick Hulisani Demana, Mehmet Öztürk, Xavier Siwe Noundou, Yves Oscar Nganso Ditchou

**Affiliations:** 1 Department of Chemistry, Faculty of Science, University of Maroua, Maroua P.O. Box 814, Cameroon; gertrudemessah@ymail.com; 2 Department of Chemistry, Faculty of Science, Muğla Sıtkı Koçman University, Kötekli 48121, Muğla, Turkey; mehmetozturk@mu.edu.tr; 3 Department of Biological Sciences, Faculty of Medicine and Pharmaceutical Sciences, University of Douala, Douala P.O. Box 2701, Cameroon; gabrielleangeamang@gmail.com; 4 Laboratory of Metabolism, Center of Research in Food, Food Security and Nutrition Research, Institute of Medical Research and Medicinal Plant Studies, Yaoundé P.O. Box 13033, Cameroon; tgroussel@yahoo.fr; 5 Laboratory of Pharmacology and Toxicology, Department of Biochemistry, Faculty of Science, University of Yaoundé 1, Yaoundé P.O. Box 812, Cameroon; briceenang@gmail.com; 6 Department of Biological Sciences, Biochemistry, Bioinformatics, and Therapeutic Innovations, Research Unit, Faculty of Science, University of Maroua, Maroua P.O. Box 814, Cameroon; mamoudhmd@gmail.com; 7 Department Basic Science Education, National Advanced School of Engineering, University of Maroua, Maroua P.O. Box 46, Cameroon; 8 Department of Chemistry, Higher Teacher Training College, The University of Bamenda, Bamenda P.O. Box 39, Cameroon; desiresoh75@gmail.com; 9 Laboratory of Phytochemistry, Centre for Research on Medicinal Plants and Traditional Medicine, Institute of Medical Research and Medicinal Plants Studies, Yaoundé P.O. Box 13033, Cameroon; feudjoufouatiow@yahoo.fr (W.F.F.); talembert@gmail.com (A.T.T.); 10 Department of Pharmaceutical Sciences, School of Pharmacy, Sefako Makgatho Health Sciences University, Pretoria 0204, South Africa; tsakemb@yahoo.fr (B.T.); madan.poka@smu.ac.za (M.P.); patrick.demana@smu.ac.za (P.H.D.); 11 Faculty of Chemistry and Chemical Technology, Al-Farabi Kazakh National University, Almaty-Kazakhstan 71, Almaty 050040, Kazakhstan

**Keywords:** *Pithecellobium dulce*, glycerol derivatives, antidiabetic activity, antioxidant activity, molecular docking

## Abstract

Background: Type 2 diabetes mellitus (T2DM) is a global health challenge characterized by chronic hyperglycemia and oxidative stress. *Pithecellobium dulce* root has long been recognized for its antidiabetic potential; however, its specific bioactive constituents and mechanisms of action remain poorly defined. This study aimed to evaluate the antidiabetic and antioxidant properties of extracts and isolated molecules from *P. dulce* root bark. Methods: The DCM/MeOH crude extract of *P. dulce* root bark was fractionated with *n*-hexane (PDEH) and ethyl acetate (PDAE), followed by chromatographic purification and spectroscopic characterization, yielding seventeen compounds (**1**–**17**). The antioxidant activity (DPPH, ABTS, FRAP) and antidiabetic potential of PDEH, PDAE, and **1**–**17** were assessed in vitro using yeast-derived enzymes and in silico (targeting human α-glucosidase [PDB: 2QLY] and human α-amylase [PDB: 4GQR]). The in vitro α-glucosidase experiments used saccharomyces cerevisiae enzyme, which varies from the human target. Therefore, these results should be taken as preliminary screening data that needs confirmation with human enzymes. Results: Compound **1** was identified as new, while **2** was isolated for the first time from a natural source. The cell-free chemical tests DPPH, ABTS, and FRAP measured antioxidant capability. These tests quantify radical-scavenging and electron-transfer capabilities in vitro and are preliminary chemical screening methods. They do not directly represent biological antioxidant activity in cells or organisms. PDEH demonstrated strong radical scavenging against DPPH (IC_50_ = 15.30 μg/mL) and ABTS (IC_50_ = 12.80 μg/mL), while pristriol (**16**) showed ferric reducing power (EC_50_ = 4200 μM FeSO_4_/g). Enzyme inhibition assays demonstrated activity against *α*-amylase (IC_50_ 53.88–112.24 µg/mL; acarbose IC_50_ = 91.20 µg/mL) and *α*-glucosidase (IC_50_ 18.38–136.88 µg/mL; acarbose IC_50_ = 11.31 µg/mL). Compounds **15**, **1**, and **2** showed superior activity compared to acarbose for α-amylase, with effect sizes (Cohen’s d) of 2.15, 0.94, and 0.82, respectively, and IC_50_ values of 53.88, 88.15, and 92.62 µg/mL; for α-glucosidase, IC_50_ values were 18.38, 39.25, and 36.40 µg/mL, respectively. Docking studies supported these findings, revealing binding energies of −9.08, −8.34, and −7.22 kcal/mol for compounds **1**, **2**, and **15** with *α*-amylase, and −10.35 and −9.79 kcal/mol for compounds **1** and **2** with *α*-glucosidase. ADME profiling further identified **1** and **2** as promising lead candidates for dual-enzyme inhibition. Conclusions: *P. dulce* root bark represents a potent source of bioactive molecules with both antioxidant and dual-enzyme-inhibitory properties. These findings validate its traditional use and highlight its potential in the development of multitarget therapies for T2DM management.

## 1. Introduction

Diabetes mellitus represents a major global public health challenge, with its prevalence reaching epidemic proportions. According to the International Diabetes Federation (IDF), approximately 537 million adults were affected in 2021, and this number is projected to rise to 783 million by 2045 [1]. Type 2 diabetes mellitus (T2DM), defined by insulin resistance and a relative insufficiency of insulin, constitutes more than 90% of all diagnosed cases [2,3,4,5]. Persistent hyperglycemia, a hallmark of this metabolic disorder, significantly contributes to the development of microvascular and macrovascular complications, including cardiovascular disease, nephropathy, retinopathy, and neuropathy, ultimately leading to increased morbidity, mortality, and substantial healthcare costs [4,6].

Oxidative stress is a fundamental mechanism connecting chronic hyperglycemia to diabetic complications. Increased glucose levels generate excessive reactive oxygen species through auto-oxidation, the polyol pathway, and advanced glycation end products [7,8,9]. This imbalance exacerbates insulin resistance, impairs *β*-cell function, and damages macromolecules, thereby accelerating disease progression [10,11]. Thus, reducing postprandial hyperglycemia and oxidative stress is critical for diabetes management. Postprandial hyperglycemia, driven by *α*-amylase and *α*-glucosidase activity, promotes carbohydrate breakdown and glucose absorption. By reducing their activity, carbohydrate digestion and glucose absorption are slowed, resulting in a flattened blood glucose curve after meals [12]. Current inhibitors, such as acarbose, miglitol, and voglibose, are effective but limited by gastrointestinal side effects from undigested carbohydrate fermentation [13]. Hence, new inhibitors with improved tolerability are needed. In this regard, medicinal plants act as a valuable source of bioactive compounds that could exhibit antidiabetic properties, often showing multi-target action mechanisms and fewer side effects [14].

*Pithecellobium dulce* (Roxb.) Benth. from the Fabaceae family is a plant of notable ethnobotanical importance. Traditionally, different parts of this plant, such as the bark, leaves, and seeds, have been utilized in various traditional medicine practices, including diabetes [15]. Previous phytochemical studies have shown that *P. dulce* contains various secondary metabolites, including flavonoids, tannins, alkylphenols, fatty acids, sterols, amino-acids, alkaloids, coumarins, anthraquinones, sugar, lignin, terpenoids, and hydrocarbon [15,16]. Significantly, multiple studies have highlighted the in vitro antidiabetic, anti-Alzheimer’s, and antioxidant properties of extracts from its leaves, seeds, and bark [16,17]. For example, fractions rich in saponins from seeds and raw extracts from bark have shown notable inhibitory potential against *α*-amylase and *α*-glucosidase [18,19].

Notwithstanding these encouraging results, comprehensive studies on the individual bioactive chemicals accountable for the reported benefits, especially from under-researched plant sections or by systematic bioactivity-guided isolation, are notably few. Furthermore, the concurrent assessment of antioxidant activity, essential for alleviating the oxidative stress linked to T2DM, frequently exhibits a weak correlation with enzyme inhibition findings. To bridge this gap, the present study focused on the extractions, isolation, and characterization of bioactive compounds from the root barks of *P. dulce* and systematically assessed their antidiabetic and antioxidant potential using both in vitro and in silico approaches.

## 2. Results

### 2.1. Chemical Characterization

**Figure 1:**
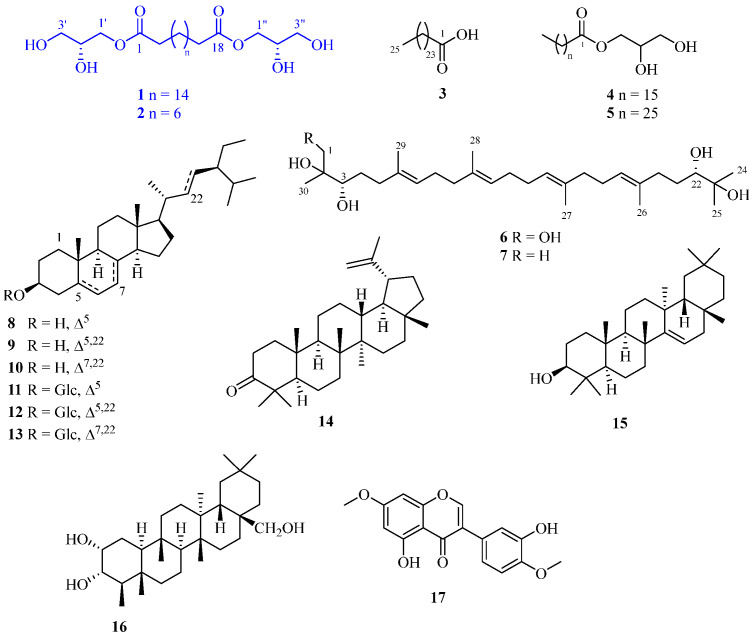
Compounds isolated and characterized (**1**–**17**); 1-((2′*R*)-2′,3′-dihydroxypropyl)-18-((2″*S*)- 2″,3″-dihydroxypropyl)octadecanedioate (**1**), 1-((2′*R*)-2′, 3′-dihydroxypropyl)-10-((2″*S*)-2″,3″-dihydroxypropyl)decanedioate (**2**), pentacosanoic acid (**3**), 1-heptadecanoylglycerol (**4**), heptacosanoate glycerol (**5**), (3*S*,6*E*,10*E*, 14*E*,18*E*,22*S*)-2,6,10,15,19,23-hexamethyltetracosa-6,10,14,18-tetraene-1,2,3, 22,23-pentaol (**6**), (3*S*,6*E*,10*E*,14*E*, 18*E*,22*S*)-2,6,10,15,19,23-hexamethyltetracosa-6,10,14,18-tetraene- 2,3,22,23-tetraol (**7**), *β*-sitosterol (**8**), stigmasterol (**9**), spinasterol (**10**), glucoside of *β*-sitosterol (**11**), glucoside of stigmasterol (**12**), glucoside of spinasterol (**13**), lupenone (**14**), taraxerol (**15**), pristriol (**16**), 7,4′-dimethyl-3′-hydroxygenistein (**17**).

The chromatographic and spectroscopic investigation of the root barks of *P. dulce* led to the structural characterization of two bisglycerol derivatives, among which compound (**1**) is new and compound (**2**) is reported for the first time from a natural source (**2**). In addition, fifteen known compounds (**3**–**17**) (Figure 1) were also characterized. All known compounds were identified as one fatty acid: pentacosanoic acid (**3**) [20], two monoglycerols: 1-heptadecanoylglycerol or heptadecanoate 2,3-dihydroxy-propyle (**4**) [21] and heptacosanoate glycerol or heptacosanoate 2′,3′-dihydroxypropyle (**5**) [22], two linear triterpenoids: (3*S*,6*E*,10*E*,14*E*,18*E*,22*S*)-2,6,10,15,19,23-hexamethy tetracosa-6,10,14,18- tetraene-1,2,3,22, 23-pentaol (**6**) [23] and (3*S*,6*E*,10*E*,14*E*,18*E*,22*S*)-2,6,10,15,19,23-hexamethyltetracosa-6,10,14, 18-tetraene-2,3,22,23-tetraol (**7**) [24], onefriedelane: friedelane- 2*α*,3*α*,28*α*-triol or pristriol (**16**) [25], one taraxerane: taraxerol (**15**) [26], one lupane: lupenone (**14**) [27], one isoflavonoid: 7,4′-dimethyl-3′-hydroxy genistein (**17**) [28] and six phytosterols: mixture of *β*-sitosterol (**8**) and stigmasterol (**9**) [29], spinasterol (**10**) [14], and a mixture of glucoside of *β*-sitosterol (**11**), glucoside of stigmasterol (**12**), and glucoside of spinasterol (**13**) [30].

Compound **1** was isolated as an amorphous white powder. C_24_H_46_O_8_ was deduced as its chemical formula, containing two double-bond equivalents from its high-resolution ESI data (Figure S1). The analysis of the ^1^H-NMR (Figure S2) together with HSQC (Figure S5) spectra revealed characteristic signals of two oxymethelene protons at *δ*_H_/*δ*_C_ 4.16 (m, 1H)-4.09 (m, 1H)/65.2 and 3.58 (t, *J* = 5.1 Hz, 2H)/62.8, together with an oxymethine proton at *δ*_H_/*δ*_C_ 3.84 (m, 1H)/69.8, which are characteristics of the glycerol moiety. In addition, the signals attributable to an aliphatic long-chain hydrocarbon without an angular methyl group were also observed at *δ*_H_/*δ*_C_ 2.37 (t, *J* = 7.6 Hz, 2H)/33.8, 1.64 (p, *J* = 7.4 Hz, 2H)/24.7, and 1.29 (brs, nH)/29.4–28.9 [31,32,33]. The absence of the aforementioned angular methyl group typical of fatty acid derivatives was further supported by the ^13^C-NMR (Figure S3) coupled to DEPT-135 (Figure S3) spectra. Nevertheless, the signals of aliphatic carbons (33.7 to 24.7 ppm) and glycerol (69.8 to 62.8 ppm) derivatives were observed. In addition, a carboxylic carbon resonance was detected at *δ*_C_ 174.4. The HMBC spectrum (Figure S6) through heteronuclear ^3^*J*_C-H_ correlations enabled identification of the main skeleton of compound **1**. Correlations between the protons at *δ*_H_ 4.16/4.09 (H1′ab/1″ab) and the carboxylic carbon at *δ*_C_ 174.4 (C1/18) supported the connectivity between the glycerol moiety and the lipophilic chain. The absence of a –Me group together with the superimposition of the 1D- and 2D-NMR data of compound **1** with those reported by Melon et al. [31], supported the duplication of the glycerol moiety and a C-9 symmetric diester [33]. The relative stereochemistry of compound **1** was suggested as 2′R and 2″S based on the comparison of our coupling constant at 3.58 (t, *J* = 5.1 Hz, 2H) with the reported value of 4.12 (d, *J* = 5.4 Hz, 2H) [31]. Finally, compound **1** was characterized as a new bisglyceride derivative named bis(2,3-dihydroxypropyl) octadecanedioate or 1-((2′*R*)-2′,3′-dihydroxypropyl) -18-((2″*S*)-2″,3″-dihydroxypropyl) octadecanedioate.

Compound **2** was isolated as an amorphous white powder. Its ^1^H- and ^13^C-NMR (Figures S8 and S9) data were superimposable to those of compound **1**, which easily enables its identification as a symmetric diester. Its molecular formula was deduced from HR-ESI (+ve) (Figure S7), which revealed a quasi-molecular ion peak [M + 3H]^3+^ at *m*/*z* 353.2140 for C_16_H_33_O_8_ with two double-bond equivalents. From this formula, we assumed that compound **2** had a C-5 symmetric plane in its lipophilic moiety. Its relative stereochemistry was deduced from its coupling constants in comparison with Melong et al.’s literature [31]. Compound **2** was previously synthesized and named 1-((2′*R*)-2′,3′- dihydroxypropyl)-10-((2″*S*)-2″,3″-dihydroxypropyl)decanedioate, as reported in the patent N° 4,661,519 of Shiga et al. This manuscript reports its first isolation from a natural source as well as its detailed NMR data.

### 2.2. Antidiabetic Effects of Crude Extracts and Compounds

**Figure 2:**
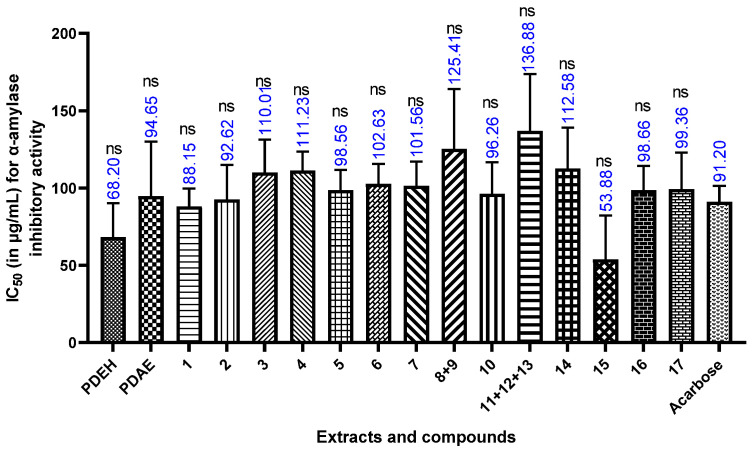
IC_50_ for *α*-amylase inhibitory activity of plant extracts and compounds: **PDEH**: Hexane extract; **PDAE**: Ethyl acetate extract, Compounds (**1**–**17**), ns: no significant difference between the IC_50_ of tested extracts/compounds and acarbose.

**Figure 3:**
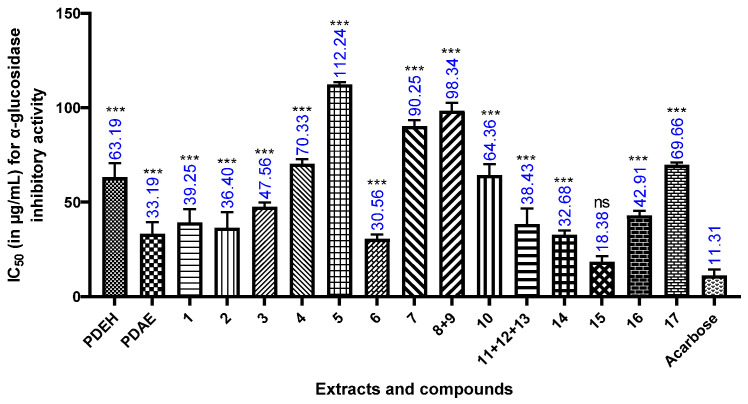
IC_50_ for *α*-glucosidase inhibitory activity of plant extracts and compounds: **PDEH**: Hexane extract; **PDAE**: Ethyl acetate extract, Compounds (**1**–**17**), ns: no significant difference between the IC_50_ of tested extracts/compounds and acarbose. **, ***: significant difference between the IC_50_ of tested extracts/compounds and acarbose respectively at *p* < 0.01 and *p* < 0.001.

The antidiabetic effect of the isolated compounds was evaluated through the inhibitory effect on *α*-amylase (Figure 2) and on *α*-glucosidase (Figure 3). The α-glucosidase used in these tests was obtained from *Saccharomyces cerevisiae* (Type I, Sigma-Aldrich, G5003), a non-human enzyme model. Although yeast α-glucosidase and human maltase-glucoamylase exhibit structural similarities, they differ in substrate selectivity, inhibitor sensitivity, and kinetic characteristics [13,14]. The in vitro IC_50_ values presented here are preliminary screening data and may not directly affect human enzyme inhibition. To provide additional target-relevant insights, complementary molecular docking studies were performed using human α-glucosidase (PDB: 2QLY). While these in silico analyses support the potential interaction of the investigated compounds with the human enzyme, experimental validation using recombinant human α-glucosidase remains necessary to confirm their inhibitory activity. All experiments were performed in triplicate, and IC_50_ values were calculated by non-linear regression (log[inhibitor] vs. normalized response). Statistical power analysis indicated that three replicates provided > 80% power to detect a 20% difference in IC_50_ at α = 0.05. The results demonstrated that both the crude extracts and the isolated compounds effectively inhibited both *α*-amylase and *α*-glucosidase. Taraxerol (**15**) was the most active against both enzymes, with an α-amylase IC_50_ of 53.88 µg/mL (95% CI: 50.2–57.8), which was significantly lower than that of acarbose (91.20 µg/mL [95% CI: 86.4–96.3]; *p* < 0.001, one-way ANOVA with Tukey’s post hoc). The effect size (Cohen’s d) for compound **15** vs. acarbose was 2.15, indicating a large practical significance. Compounds **1** and **2** also outperformed acarbose for α-amylase, with IC_50_ values of 88.15 µg/mL (95% CI: 83.1–93.5; d = 0.94) and 92.62 µg/mL (95% CI: 87.4–98.2; d = 0.82), respectively. For α-glucosidase, compound **15** gave an IC_50_ of 18.38 µg/mL (95% CI: 16.9–20.0), which was higher than acarbose (11.31 µg/mL [95% CI: 10.2–12.5]; *p* < 0.05), but still within a therapeutically relevant range. Compounds **1** and **2** showed IC_50_ values of 39.25 µg/mL (95% CI: 36.1–42.7) and 36.40 µg/mL (95% CI: 33.5–39.6), respectively. The mixture of β-sitosterol glucoside (**11**), stigmasterol glucoside (**12**), and spinasterol glucoside (**13**) was the least active against α-amylase, IC_50_ = 112.24 µg/mL, while heptacosanoate glycerol (**5**) was the least active against α-glucosidase, IC_50_ = 136.88 µg/mL. The statistical analysis revealed no significant difference between Taraxerol (**15**) and the reference drug, acarbose (IC_50_ of 11.31 µg/mL), but instead a significant difference (*p* < 0.001) between acarbose and the other compounds and crude extracts.

### 2.3. In Vitro Chemical Radical-Scavenging and Reducing Capacity of Crude Extracts and Compounds

The antioxidant activity was determined through the DPPH and an ABTS radicals-scavenging assay, and the ferric reducing antioxidant power.

#### 2.3.1. Scavenging Effects of Crude Extracts and Compounds

**Figure 4:**
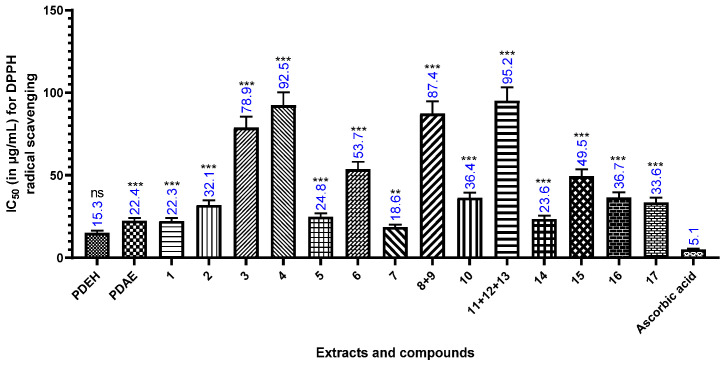
IC_50_ for DPPH radical-scavenging activity of plant extracts and compounds. **Note**: This cell-free chemical assay measures hydrogen atom transfer capacity and does not directly represent biological antioxidant activity. **PDEH**: Hexane extract; **PDAE**: Ethyl acetate extract, Compounds (**1**–**17**), ns: no significant difference between the IC_50_ of tested extracts/compounds and Ascorbic acid **, ***: significant difference between the IC_50_ of tested extracts/compounds and Ascorbic acid, respectively, at *p* < 0.01 and *p* < 0.001.

**Figure 5:**
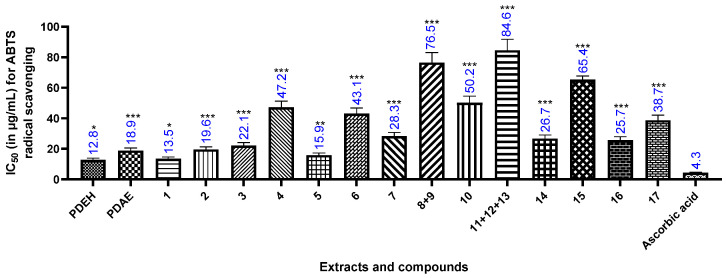
IC_50_ for ABTS radical-scavenging activity (electron transfer assay) of plant extracts and compounds**: PDEH**: Hexane extract; **PDAE**: Ethyl acetate extract, Compounds (**1**–**17**), *, **, ***: significant difference between the IC_50_ of tested extracts/compounds and Ascorbic acid, respectively, at *p* < 0.05, *p* < 0.01 and *p* < 0.001.

The results demonstrated that both the crude extracts and the isolated compounds effectively scavenged both DPPH (Figure 4) and ABTS (Figure 5) radicals. The crude hexane extract (**PDEH**) was the most active, and the mixture of *β*-sitosterol glucoside (**11**) + stigmasterol glucoside (**12**) + spinasterol glucoside (**13**) was the least active for the two radicals. For the DPPH radical, the efficacy of the tested samples varied, with IC_50_ ranging from 15.30 µg/mL to 95.20 µg/mL. The statistical analysis revealed no significant difference between the crude hexane extract (**PDEH**) and the reference, ascorbic acid (IC_50_ of 5.10 µg/mL), but a significant difference (at least *p* < 0.01) was revealed between ascorbic acid and all the other compounds and the Ethyl acetate extract (**PDAE)**. For the ABTS radical, the efficacy of the tested samples varied, with IC_50_ ranging from 12.80 µg/mL to 84.60 µg/mL. The statistical analysis revealed a significant difference between the different crude extracts and compounds, with the reference, ascorbic acid (IC_50_ of 4.30 µg/mL).

#### 2.3.2. Ferric Reducing Power of Crude Extracts and Compounds

**Figure 6:**
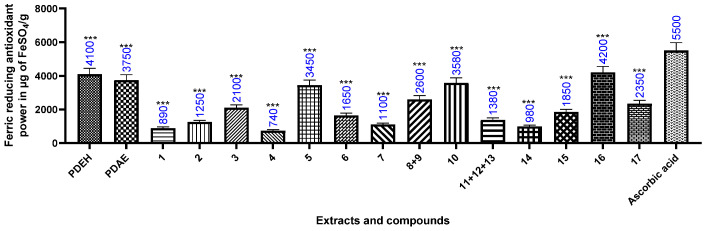
EC_50_ for ferric reducing antioxidant power activity of plant extracts and compounds: **Note:** FRAP measures chemical reducing capacity under acidic conditions; results do not directly represent cellular antioxidant activity. **PDEH**: Hexane extract; **PDAE**: Ethyl acetate extract, Compounds (**1**–**17**), ***: significant difference between the IC_50_ of tested extracts/compounds and Ascorbic acid, respectively at *p* < 0.001.

The results demonstrated that both the crude extracts and the isolated compounds effectively have reducing power (Figure 6). Pristriol (**16**) was the most active alongside 1-heptadecanoylglycerol (**4**). The efficacy of the tested samples varied, with EC_50_ ranging from 700 µM FeSO_4_/g to 4200 µM FeSO_4_/g. The statistical analysis revealed a significant difference between the different crude extracts (Hexane extract (**PDEH**), Ethyl acetate extract (**PDAE**)), and all the tested compounds, with the reference, ascorbic acid (EC_50_ of 5500 µM FeSO_4_/g).

### 2.4. Binding Score Analysis 

The molecular docking investigations were performed on all characterized molecules (**1**–**17**). Each molecule was treated individually, even when identified as part of a mixture. The docking study targeted human maltase-glucoamylase (*α*-Glucosidase, PDB, Id: 2QLY) and human pancreatic alpha-amylase (*α*-Amylase, PDB, Id: 4GQR), two key enzymes implicated in type II diabetes. Acarbose was employed as the reference drug (Table 1). Binding energies lower than −4.25 kcal/mol are generally regarded as indicative of favorable compound–target interactions [34]. For the purposes of this study, however, compounds were considered noteworthy only if their binding energies (BE) were less than or equal to −7.00 kcal/mol (BE ≤ −7.00 kcal/mol) [14,35,36].

### 2.5. ADME Interpretation

The aim of the ADME studies was to further filter the isolated compounds from *P. dulce* as candidates for clinical trials and to evaluate their bioavailability and metabolic stability. Compounds **1**–**7** and **11**–**13** (Tables S5 and S6) were selected based on their lower binding energies (BE ≤ −9.00 kcal/mol) close to those of acarbose (−9.70 kcal/mol, *α*-amylase and −9.42, *α*-glucosidase) for further evaluation of their druggability.

## 3. Discussion

### 3.1. Chemical Significances

The root barks of *P. dulce* were extracted with a system of DCM/MeOH (1:1, *v*:*v*) and further extracted with *n*-hexane (PDEH) and ethyl acetate (PDAE) respectively. PDEH and PDAE were chromatographed with normal-phase CC, leading to the isolation of seventeen compounds (**1**–**17**). These compounds were all characterized based on their 1D- and 2-NMR, MS, and by comparison with the reported literature. The characterized compounds were grouped as two bisglycerol derivatives (**1**–**2**), one fatty acid (**3**), two monoglycerols (**4**–**5**), five triterpenoids classified as two linear triterpenoids (**6**–**7**), one friedelane (**16**), one taraxerane (**15**), and one lupine (**14**), one isoflavonoid (**17**), and six phytosterols including a mixture of *β*-sitosterol (**8**) and stigmasterol (**9**), spinasterol (**10**), and a mixture of glucoside of *β*-sitosterol (**11**), glucoside of stigmasterol (**12**), and glucoside of spinasterol (**13**). The presence of this plethora of classes of natural products is not a fortuitous event, as recent investigations using state-of-the-art instruments (UPLC-ESI–MS/MS) revealed their existence or derivatives [16].

### 3.2. Bioactive Significances

#### 3.2.1. In Vitro Significances

Type 2 diabetes mellitus is defined by persistent hyperglycaemia linked to elevated oxidative stress and heightened activity of critical digestive enzymes, including α-amylase and α-glucosidase. This enzymatic hyperactivity intensifies postprandial glucose fluctuations and leads to *β*-cell impairment and insulin resistance [37]. Inhibitors of these enzymes are therefore recognized as an effective therapeutic strategy for controlling postprandial hyperglycaemia and limiting long-term complications [11,38,39]. In the present study, the hexane and ethyl acetate extracts of *P. dulce*, as well as several isolated compounds, exhibited significant inhibitory activity against α-amylase and α-glucosidase, with IC_50_ values comparable to acarbose. These results indicate a genuine potential to reduce postprandial hyperglycaemia through the inhibition of key carbohydrate-digesting enzymes. These findings are consistent with previous reports on *P. dulce* bark, seeds, and leaves, where extracts and saponin-enriched fractions markedly inhibited these enzymes in vitro and improved glycemic control in animal models [17].

At the molecular level, taraxerol (**15**), the most active compound identified in this study, is a pentacyclic triterpene whose inhibitory activity against α-amylase and α-glucosidase has been supported by docking and in silico analyses. These studies demonstrate strong binding within the catalytic groove, driven mainly by hydrophobic interactions and stabilized by a limited number of hydrogen bonds [19,40]. More broadly, triterpenes are increasingly recognized as antidiabetic agents capable of inhibiting digestive enzymes, modulating insulin signaling, and protecting *β*-cells from glucotoxic, lipotoxic, and oxidative damage [41]. Similarly, the friedelane-type triol and related triterpenoid polyols isolated in this work combine a large hydrophobic scaffold with multiple hydroxyl groups. This structural configuration allows effective occupation of hydrophobic subsites of the enzymes while providing hydrogen-bond donors for catalytic or neighboring residues, a binding pattern reported for triterpenoids in several α-glucosidase studies [42].

Phytosterols such as *β*-sitosterol (**8**) and stigmasterol (**9**), along with their glycosides (**11**–**13**), have been associated with improved glycemic regulation and insulin sensitivity in both experimental and clinical studies. These effects are partly mediated through the modulation of carbohydrate metabolism, the enhancement of GLUT4-mediated glucose uptake, and a reduction in pro-inflammatory signaling [43]. Although their direct inhibitory effect on α-amylase and α-glucosidase may be weaker than that of triterpenes, these compounds likely contribute to the overall antihyperglycaemic effect through complementary metabolic mechanisms. In contrast, long-chain glycerol esters and free fatty acids showed only moderate enzyme inhibition, suggesting that high hydrophobicity alone is insufficient for strong activity and that the number and arrangement of polar functional groups are critical for effective binding. Altogether, the enzyme inhibition profile observed for *P. dulce*, involving pentacyclic triterpenes, phytosterols, and lipid-derived polyols, supports the previously described multi-target antidiabetic action of this species and other triterpene-rich medicinal plants [44,45].

Chronic hyperglycaemia in type 2 diabetes is significantly associated with oxidative stress via processes including increased mitochondrial superoxide generation, formation of advanced glycation end products, activation of the polyol pathway, and protein kinase C activation. These mechanisms elevate reactive oxygen and nitrogen species, resulting in β-cell malfunction and vascular problems [41]. In this context, the pronounced antioxidant activity observed for *P. dulce* extracts and several isolated compounds is mechanistically relevant. Both crude extracts demonstrated radical-scavenging activity against DPPH and ABTS, while the most active fractions and triterpenes exhibited low IC_50_ values and significant ferric-reducing power, in some cases approaching that of ascorbic acid. This pattern is consistent with previous reports where triterpene- and phenolic-rich extracts displayed strong DPPH and ABTS scavenging activities, high FRAP values, and elevated polyphenol content associated with protection against diabetes-related oxidative stress [46].

The isoflavone-type compound 7,4′-dimethyl-3′-hydroxygenistein (**17**) identified in *P. dulce* presents classical structural features of flavonoid antioxidants, including an extended conjugated system and phenolic hydroxyl groups. These features enable efficient hydrogen atom or electron donation and resonance stabilization of the resulting radicals, as described for genistein and related flavonoids [47]. Triterpenes further contribute to the antioxidant effect. Numerous studies report that pentacyclic triterpenes reduce diabetes-induced oxidative damage by scavenging free radicals, enhancing endogenous antioxidant enzymes (SOD, catalase, GPx), and inhibiting pro-oxidant inflammatory pathways in pancreatic and peripheral tissues [41]. Phytosterols, although weaker radical scavengers, contribute by stabilizing cellular membranes, reducing lipid peroxidation, and improving overall redox balance [48,49]. Therefore, the antioxidant properties observed for *P. dulce* are fully consistent with its chemical composition, dominated by triterpenes, phytosterols, and flavonoid-like compounds, and with the established roles of these metabolites in restoring redox homeostasis in diabetes.

#### 3.2.2. Molecular Docking Interactions Analysis (α-Glucosidase, 2QLY and α-Amylase, 4GQR)

##### Alpha-Glucosidase (2QLY) Significances

For *α*-glucosidase (2QLY), acarbose achieved a score of −9.42 kcal/mol, with primary interactions with Arg520, Ala509, Leu286, Met567, Lys776, and Ile523 amino-acid residues (Table 2). These amino-acids are well known to exert protective effects in diabetes management, even though Alanine and Methionine require careful consideration due to their potential adverse roles. The aforementioned amino-acids have been implicated in the modulation of glucose metabolism and the prevention of type II diabetes. Arginine (Arg) has been shown to stimulate insulin secretion through nitric oxide-mediated pathways and to improve endothelial function, thereby enhancing glucose uptake [50,51]. Leucine (Leu) and Isoleucine (Ile), both branched-chain amino-acids, act as potent stimulators of insulin release via activation of the mechanistic Target Of Rapamycin (*m*TOR pathway) pathway and incretin secretion, respectively, contributing to improved glucose tolerance [52,53]. Lysine (Lys) supplementation has been reported to reduce the formation of advanced glycation end products (AGEs), offering protection against diabetic complications [54]. Alanine (Ala) plays a dual role: while it can stimulate insulin release in pancreatic *β*-cells, elevated plasma Alanine levels are often associated with insulin resistance [55,56]. In contrast, excessive Methionine (Met) intake has been linked to oxidative stress and impaired insulin sensitivity, whereas Methionine restriction has demonstrated beneficial metabolic effects [57].

The docked compounds that shared specific residues with acarbose include: compound **1** (−10.35 kcal/mol), which shares Leu286 (H-donor and H-acceptor), Met567 (H-donor), and Ile523 (H-acceptor); compound **2** (−9.79 kcal/mol), sharing Met567 (H-donor), Lys776 (H-acceptor), and Leu286 (H-acceptor); compound **3** (−10.58 kcal/mol), sharing Met567 (H-acceptor); compound **4** (−10.58 kcal/mol), sharing Ala285 (H-donor); compound **5** (−10.20 kcal/mol), sharing Leu286 (H-donor); compound **11** (−7.33 kcal/mol), sharing Lys513 (H-acceptor); and compounds **7** (−9.93 kcal/mol), **6** (−10.75 kcal/mol), **12** (−8.08 kcal/mol), and **17** (−7.78 kcal/mol), all of which share the Met567 residue via H-donor or H-acceptor interactions. The significantly lower binding energies observed for compounds **1**–**7**, often surpassing the affinity of acarbose, are primarily driven by consistent and strong interactions with Leucine and/or Methionine. These residues may act as critical anchors within the substrate-binding tunnel. In addition, the acyclic structural characteristics of these compounds, coupled with their functional similarities: multiple hydroxyl and carboxylic groups, alongside long aliphatic chains (alcohol, fatty acids and esters), favor the formation of specific polar hydrogen bonds and extensive hydrophobic stabilization, which results in superior binding energies compared to the reference.

The computational *α*-glucosidase outcomes of several compounds show a positive correlation with their experimental inhibitory activities. Compound **6**, with the most favorable docking score (−10.75 kcal/mol), demonstrated one of the strongest in vitro activities (IC_50_ of 30.56 μg/mL). In addition, compounds **1** and **2** exhibited high binding affinities (−10.35 and −9.79 kcal/mol, respectively) and showed potent inhibition (IC_50_ values of 39.25 and 36.40 μg/mL, respectively). On the other hand, significant disparities exist for certain compounds, suggesting that experimental or theoretical approaches alone do not always provide absolute biological potency. Compound **15** presents the most striking contrast. It has a highly unfavorable docking score (+1.00 kcal/mol) while being the most potent natural *α*-glucosidase inhibitor with an IC_50_ of 18.38 μg/mL. Similarly, lupenone (**14**), another cyclic triterpenoid, revealed a comparable discrepancy (docking score of −2.31 kcal/mol, IC_50_ of 32.68 μg/mL). These observations may be attributed to their main skeleton and functional groups [14], or to the possibility that these compounds may bind to allosteric sites or via mechanisms that the current docking model (focused on the acarbose binding pocket) did not fully capture.

##### Alpha-Amylase (4GQR) Significances

Acarbose was utilized as the reference medication to examine α-amylase, similar to its application for α-glucosidase. Acarbose demonstrates a docking score of −9.70 kcal/mol and engages with residues Asp300 (H-donor), Asp197 (H-donor), Ala106 (H-acceptor), Glu233 (H-donor), His299 (H-acceptor), and Tyr62 (H-pi) (Table 3). The catalytic functions of Glutamate (Glu) and Aspartate (Asp) involve acting as an acid/base catalyst and a nucleophile, respectively, facilitating the hydrolysis of α 1,4-glycosidic linkages and so serving as major anchors for inhibitor binding [58]. Histidine (His) contributes to the stabilization of substrate orientation and chloride ions (Cl^−^), and its modification is known to markedly reduce enzymatic activity [59]. To the best of our knowledge, Alanine (Ala) and Tyrosine (Tyr) residues are not directly involved in the catalytic process of *α*-amylase. However, Alanine (Ala) provides hydrophobic support that helps position inhibitors within the active site pocket [55,60], while Tyrosine (Tyr) regulates substrate specificity and cleavage patterns, ensuring proper recognition of inhibitors such as acarbose [61].

Among the docked compounds, only compound **17** did not show noteworthy binding energy values (BE ≤ −7.00 kcal/mol). This is because **17** established an H-donor with Thr163 and a 6-ring π-H interaction with Leu165, which are different from those established by the reference (acarbose). On the other hand, compounds (**1**–**4**, **6**–**13**, and **15**) met the selective criteria (BE ≤ −7.00 kcal/mol) (Table 1) by sharing at least one of their interactive amino-acid residues with those of acarbose, suggesting a similar mechanism of action. Compound **1** (−9.08 kcal/mol) interacts with Glu233 (H-donor) and His299 (H-acceptor). Compound **2** (−8.34 kcal/mol) shares Asp300 (H-donor) and Glu233 (H-donor). Compound **3** (−8.52 kcal/mol) interacts with Glu233 (H-donor). Compound **4** (−8.06 kcal/mol) shares Asp300 (H-donor), Glu233 (H-donor), and His299 (H-acceptor). Compound **6** (−9.64 kcal/mol) shares Asp300 (H-donor). Compound 7 (−9.50 kcal/mol) shares Glu233 (H-donor), Asp197 (H-donor), and His299 (H-acceptor). Compound **8** (−7.73 kcal/mol) shares Glu233 (H-donor). Compound **9** (−7.98 kcal/mol) shares Asp197 (H-donor). Compound **10** (−7.86 kcal/mol) shares Asp197 (H-donor). Compound **11** (−9.63 kcal/mol) shares Glu233 (H-donor), Asp300 (H-donor), and His299 (H-acceptor). Compound **12** (−9.15 kcal/mol) shares Asp197 (H-donor), Glu233 (H-donor), and His299 (H-acceptor). Compound **13** (−9.35 kcal/mol) shares Glu233 (H-donor), Asp300 (H-donor), Asp197 (H-donor), and His299 (H-acceptor). And compound **15** (−7.22 kcal/mol) shares Asp197 (H-donor). In contrast, the remaining docked compounds (**5**, **14**, and **16**) met the selective criteria (BE ≤ −7.00 kcal/mol) without sharing their interactive amino-acid residues with the reference drug. Compound **5** (−9.34 kcal/mol) established an H-acceptor interaction with Lys200, compound **8** (−7.86 kcal/mol) established an H-donor interaction with Thr163, and compound **17** (−7.45 kcal/mol) formed a 6-ring H-π interaction with Trp59. Based on the aforementioned, these three residues (Lys, Thr, and Trp) may play a significant role in the inhibition of *α*-amylase.

The in silico *α*-amylase outcomes of several compounds align with their experimental inhibitory activities. Compounds **1** and **2**, which are chain isomers, exhibited a favorable BE of −9.08 and −8.34 kcal/mol, respectively, matching their strong inhibitory activities (IC_50_ = 88.15 and 92.62 μg/mL, respectively). In addition, compound **1** is slightly more potent than the reference drug, and its isomer (compound **2**) is very close to the reference in the in vitro test. Other chain isomers, comprising compounds **4** and **5**, exhibited favorable BE of −8.52 and −9.34 kcal/mol, respectively, and also demonstrated moderate-to-strong in vitro inhibitory effects (IC_50_ of 111.23 and 98.56 μg/mL, respectively). The functional isomers, comprising compounds **6** and **7**, demonstrated high BE (−9.64 and −9.50 kcal/mol, respectively) and showed moderate-to-strong experimental inhibition (IC_50_ of 102.63 and 101.56 μg/mL, respectively). The correlations invoked above are supported by the molecular isomerism, functional groups, as well as their ability to form multiple hydrogen bonds with the enzyme’s catalytic triad (Asp197, Glu233, and Asp300), effectively mimicking the binding mode of acarbose [62,63]. In contrast, discrepancies occur when compounds show high biological activity despite modest docking scores, or vice versa. Similar to *α*-glucosidase, compound **15** (taraxerol) presents the strongest contrast. It has a relatively modest docking score of −7.22 kcal/mol yet emerges as the most potent inhibitor in vitro (IC_50_ of 53.88 μg/mL). Compounds **11**–**13** (phytosterol glycosides) show very high docking affinities (ranging from −9.15 to −9.63 kcal/mol), while their experimental mixture (**11** + **12** + **13**) yielded the lowest in vitro potency (IC_50_ of 136.88 μg/mL). This indicates that these compounds are antagonists when mixed.

#### 3.2.3. ADME Significances

**Figure 7:**
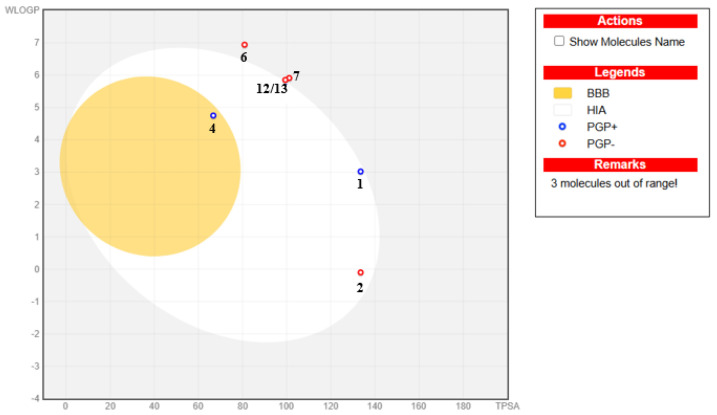
Boiled-egg graph of compounds **1**–**7** and **11**–**13**.

Among the tested compounds, solubility profiles exhibit significant variation, ranging from very soluble to poorly soluble (Figure 7). Compound **2** is identified as the most soluble molecule according to the “ESOL”/”Ali” solubility models (Log S: 0.99/−2.51), followed by compound **1** (Log S: −3.88/−7.00). This solubility is attributed to their polyhydroxylation, characterized by four free –OH groups and two ester groups, as well as their short lipophilic moieties. The solubility of these dihydroxypropyl esters (**1** and **2**) is further consistent with their high values of Topological Polar Surface Area (TPSA) (133.52 Å^2^). In contrast, long-chain fatty acid and sterol derivatives (compounds **3**–**7** and **11**–**13**) are either moderately or poorly soluble due to their dominant lipophilic fragments, with their TPSA ranging from 37.30 to 101.15 Å^2^ (Table 4).

In regard to pharmacokinetics properties, Gastrointestinal (GI) absorption was found to be high for compounds **1**, **2**, **4**, **12**, and **13**, making them the most promising candidates for oral administration (Table 4). With the exception of compound **4**, all the evaluated compounds are not blood–brain barrier (BBB) permeant, which is ideal for avoiding central nervous system side effects. This observation is also evident in the boiled-egg diagram (Figure 7). On the other hand, compounds **1**, **4**, **12**, and **13** are P-glycoprotein (P-gp) substrates, which may influence their efflux and overall bioavailability.

Regarding the druglikeness of these compounds, Lipinski’s Rule of Five (RoF) was not violated by compounds **1** and **2**. The remaining compounds showed a single RoF violation, which was either MLOGP > 4.15 (for **3**, **5**, **6**, and **7**) or molecular weight (MW > 500 Da, for **11**, **12**, and **13**). Despite these RoF violations, their bioavailability scores were either 0.55 (for **1**, **2**, **4**–**7**, and **11**–**13**) or 0.85 for compound **4**. These observations suggest that these compounds possess promising drug-like properties for further investigations [64]. From a medicinal chemistry perspective, compounds (**1**–**7** and **11**–**13**) were free of Pan-Assay Interference Structures (PAINS) alerts, which reduces the risk of false-positive results in biological assays. In addition, the first seven compounds (**1**–**7**) can be easily synthesized based on their synthetic accessibility scores ranging from 3.36 to 5.22. From this investigation, it clearly appears that compounds **1**, **2**, **12**, and **13** are good candidates for drug development. However, more efforts should be directed toward **1** and **2**, as they can be easily synthesized and/or isolated, while **12** and **13** are rarely isolated alone and not easy to prepare. In addition, **1** and **2** integrated promising docking scores, in vitro, and ADME data, making them the lead candidates for *α*-amylase and *α*-glucosidase inhibition.

### 3.3. Study Limitations

Several methodological constraints should be noted. First, the in vitro *α*-glucosidase assays employed *Saccharomyces cerevisiae* enzyme rather than human MGAM or SI, a common phytochemical screening model that does not fully replicate the intestinal environment; confirmation with human recombinant enzymes is therefore required before inferring antidiabetic activity. Second, molecular docking provides static representations that neglect protein flexibility, solvation, and kinetic factors; complementary molecular dynamics simulations could enhance binding predictions. Third, ADME outcomes are computational estimates and must be validated experimentally, particularly regarding intestinal permeability and metabolic stability. Fourth, standardized antioxidant assays assess chemical reactivity but not intracellular or in vivo oxidative stress modulation; animal studies in diabetic models are needed to establish pharmacokinetic–pharmacodynamic relationships and therapeutic efficacy. Overall, the bioactivity data derive from in vitro and in silico approaches, and while these limitations frame the scope of the findings, they highlight directions for future research rather than diminishing the study’s contributions.

## 4. Materials and Methods

### 4.1. General Experimental Procedures

The mass of extracts and compounds was measured using electronic balances (Mettler PT 22 and METLAR). The filtrate obtained from maceration was concentrated under vacuum utilizing a rotary evaporator (Heidolph Instruments GmbH & Co. KG, Schwabach, Germany). Liquid–liquid separation was conducted using a 1 L separating funnel, and purifications were executed via normal phase column chromatography (CC) employing silica gel KIESELGEL 60 (0.04–0.063 mm; Merck, Darmstadt, Germany) as the stationary phase. MeOH, EtOAc, DMSO, DCM and *n*-hexane, among other solvents, were used (deuterated for NMR or commercial as mobile phases for CC). Chromatographic monitoring was achieved by TLC on silica gel 60F_254_ plates (Merck), visualized under UV light (254/366 nm) and by heating in an oven (VENTTI-Line, ≈ 60 °C). Structural characterization was established by mass spectrometries (Varian MAT 731 for electron impact and LCQ TOF for electrospray ionization) as well as by 1D- and 2D-NMR spectroscopies (Bruker Avance 400, 500, 600, and 700 MHz). For NMR analyses, tetramethylsilane (TMS) was used as an internal reference, and chemical shifts (*δ*) and coupling constants (*J*) were calculated in ppm and Hertz (Hz), respectively. All solvents used for extraction and chromatography (*n*-hexane, ethyl acetate, dichloromethane, and methanol) were of analytical grade (≥99.5% purity) and obtained from Sigma-Aldrich (Johannesburg, South Africa) and were distilled before use.

### 4.2. Plant Material

The root bark of *Pithecellobium dulce* (Roxb.) Benth. was collected in March 2022 between 5:30 and 6:00 a.m. in the city of Maroua (collection point: 10°35′ N, 14°19′ E), Diamaré Division, Far North Region, Cameroon. The plant was authenticated by Dr. Ngansop Tchatchouang Éric, a botanist at the Cameroon National Herbarium in Yaoundé. Identification was confirmed by comparison of our samples with a specimen from the collection of the National Herbarium of Cameroon under voucher 2695 SRF/CAM.

### 4.3. Extraction and Isolation

The root barks of *P. dulce* were cut, shade-dried, and finely ground to yield 4500.0 g of powder. This powder was subjected to a triple-extraction maceration in dichloromethane (DCM)/methanol (MeOH) (1:1, *v*:*v*; 5 L) system for 72 h. The filtrate was evaporated with a rotary evaporator (≈ 60 °C) under reduced pressure, yielding 500.1 g of crude extract. A portion of 400.0 g of this extract was dissolved in 3 L of MeOH/H_2_O (1:9, *v*:*v*) and sequentially partitioned with *n*-hexane (5 L; 151.4 g) (PDEH) and ethyl acetate (5 L; 215.2 g) (PDAE). All organic solvents used were of analytical grade (≥99.5%), purchased from Sigma-Aldrich (South Africa), and were distilled before use.

In the first purification part, an open CC on silica gel was employed for the fractionation of 140.0 g of the *n*-hexane extract (PDEH). Elution was performed with solvents of increasing polarity: *n*-hexane, *n*-hexane/ethyl acetate (EtOAc), and EtOAc. A total of 364 fractions (~200 mL each) were collected and combined into seven major series (PDRF1–PDRF7) based on their TLC similarities. The purification of PDRF2 (Fractions 21–38, 2.3 g) by means of an open CC (SiO_2_, isocratic, *n*-hexane/EtOAc 7.5:2.5, *v*:*v*) yielded compound **3** (10.3 mg, white powder). The purification of PDRF3 (Fractions 50–80, 1.9 g) using an open CC (SiO_2_, isocratic, *n*-hexane/EtOAc 9:1: *v*:*v*) yielded a mixture of compounds **8** and **9** (9123.6 mg, white needle powder). The purification of PDRF4 (Fractions 122–165, 5.9 g) using open CC (SiO_2_, isocratic, *n*-hexane/EtOAc 8.5:1.5: *v*:*v*) led to compound **10** (125.6 mg, white needle powder). The purification of PDRF5 (Fractions 192–215, 7.9 g) using open CC (SiO_2_, isocratic, *n*-hexane/EtOAc 8.5:1.5: *v*:*v*) led to compound **14** (100.7 mg, white powder). And finally, the purification of PDRF6 (Fractions 216–2220, 10.9 g) using open CC (SiO_2_, isocratic, *n*-hexane/EtOAc 8.5:1.5: *v*:*v*) led to compound **14** (2730.2 mg, white powder).

In the second part, a total of 200.0 g of the EtOAc extract (PDAE) was fractionated with an open CC on silica gel eluted with *n*-hexane/EtOAc, EtOAc, and EtOAc/MeOH. A total of 385 fractions (~200 mL each) were collected and combined into 16 series (PDRAF1–PDRAF16) based on their TLC profiles. The purification of PDRAF2 (Fractions 22–32, 10.5 g) using open CC (SiO_2_, isocratic, *n*-hexane/EtOAc 8:2: *v*:*v*) yielded compounds **4** (109.6 mg, white powder) and **5** (129.1 mg, white powder). The purification of PDRAF3 (Fractions 56–60, 11.5 g) using open CC (SiO_2_, isocratic, *n*-hexane/EtOAc 7.5:2.5: *v*:*v*) yielded compounds **1** (9.6 mg, white powder) and **2** (7.1 mg, white powder). The purification of PDRAF4 (Fractions 66–77, 11.5 g) using open CC (SiO_2_, isocratic, *n*-hexane/EtOAc 8:2: *v*:*v*) yielded compound **15** (8.6 mg, white powder). The purification of PDRAF7 (Fractions 66–77, 13.6 g) using open CC (SiO_2_, isocratic, *n*-hexane/EtOAc 8:2: *v*:*v*) yielded compound **16** (20.2 mg, white powder). The purification of PDRAF8 (Fractions 95–100, 3.7 g) using open CC (SiO_2_, isocratic, EtOAc) yielded compound **17** (20.2 mg, pale yellow powder). The purification of PDRAF10 (Fractions 166–170, 13.6 g) using open CC (SiO_2_, isocratic, EtOAc/MeOH 9:1: *v*:*v*) yielded compounds **6** (2.2 mg, white powder) and **7** (3.2 mg, white powder). And finally, the purification of PDRAF11 (Fractions 226–230, 20.6 g) using open CC (SiO_2_, isocratic, EtOAc/MeOH 8:2: *v*:*v*) yielded a mixture of compounds **11**, **12**, and **13** (3223.1 mg, brown powder).

### 4.4. Spectroscopic and Spectrometric Data Compounds (***1**–**17***)

***1-((2′R)-2′,3′-dihydroxypropyl)-18-((2″S)-2″,3″-dihydroxypropyl)octadecanedioate or bis(2,3-dihydroxypropyl)octadecanedioate (1)***: ^1^H NMR (500 MHz, Methanol-d_4_) δ_H(ppm)_ 4.16 (m, 1H) (H1′a/1″a), 4.09 (m, 1H) (H1′b/1″b), 3.85 (m, 1H) (H2′/2″), 3.58 (t, *J* = 5.1 Hz, 2H) (H3′/3″), 2.37 (t, *J* = 7.6 Hz, 2H) (H2/17), 1.64 (p, *J* = 7.4 Hz, 2H) (H3/16), 1.29 (s, 24H) (H4–15). ^13^C NMR (126 MHz, Methanol-d_4_) δ_C(ppm)_ 174.4 (C1/18), 69.8 (C2′/2″), 65.2 (C1′/1″), 62.8 (C3′/3″), 33.8 (C2/17), 29.4–28.9 (C4–15), 24.7 (C3/16). HR-ESI (+ve) [M + 2H + Na]^3+^ *m*/*z* 487.3285, Calculated for *m*/*z* 487.3247, C_24_H_48_O_8_Na (Figures S1–S6).

***1-((2′R)-2′,3′-dihydroxypropyl)-10-((2″S)-2″,3″-dihydroxypropyl)decanedioate (2)***: ^1^H NMR (500 MHz, Methanol-*d*_4_) *δ*_H(ppm)_ 4.16 (dd, *J* = 11.4, 4.5 Hz, 1H) (H1′a/1″a), 4.09 (dd, *J* = 11.4, 6.2 Hz, 1H) (H1′b/1″b), 3.85 (p, *J* = 5.5 Hz, 1H) (H2′/2″), 3.58 (t, *J* = 5.1 Hz, 2H) (H3′/3″), 2.36 (t, *J* = 7.6 Hz, 2H) (H2/9), 1.63 (p, *J* = 7.5 Hz, 2H) (H3/8), 1.29 (s, 8H) (H4–7). ^13^C NMR (125 MHz, Methanol-*d*_4_) *δ*_C(ppm)_ 174.3 (C1/10), 69.7 (C2′/2″), 65.1 (C1′/1″), 62.7 (C3′/3″), 33.7 (C2/9), 29.3–28.1 (C4–7), 24.6 (C3/8). HR-ESI (+ve) [M + 3H]^3+^ *m*/*z* 353.2140, Calculated for *m*/*z* 353.2175, C_16_H_33_O_8_ (Figures S7–S9).

***Pentacosanoic acid (3)***: ^1^H NMR (400 MHz, CDCl_3_) *δ*_H(ppm)_ 10.03 (s, 1H), 2.33 (t, *J* = 7.5 Hz, 2H), 1.61 (t, *J* = 7.3 Hz, 2H), 1.23 (s, nH), 0.86 (t, *J* = 6.6 Hz, 3H). EI, M^+^ *m*/*z* 382, Calculated for *m*/*z* 382.38, C_25_H_50_O_2_ (Figures S10 and S11).

***1-heptadecanoylglycerol (4)***: ^1^H NMR (500 MHz, CDCl_3_) *δ*_H(ppm)_ 4.22–4.09 (m, 2H), 3.95–3.88 (m, 1H), 3.71–3.55 (m, 2H), 2.33 (td, *J* = 7.5, 4.1 Hz, 2H), 1.23 (s, 51H), 0.86 (t, *J* = 6.9 Hz, 3H). ^13^C NMR (125 MHz, CDCl_3_) *δ*_C(ppm)_ (Ordered C1-C17; C1′-C3′) 174.6, 34.4, 22.9, 29.9–29.3, 25.1, 32.1, 14.3; 65.4, 70.5, 63.5. HR-ESI, [M + NH_4_]^+^ *m*/*z* 362.3247, Calculated *m*/*z* 362.3265, C_20_H_44_NO_4_ (Figures S12–S14).

***Heptacosanoate glycerol (5)***: ^1^H NMR (500 MHz, CDCl_3_) *δ*_H(ppm)_ 4.24–4.11 (m, 2H), 3.97–3.90 (m, 1H), 3.73–3.57 (m, 2H), 2.35 (td, *J* = 7.5, 4.1 Hz, 2H), 1.25 (s, 59H), 0.88 (t, *J* = 6.9 Hz, 3H). ^13^C NMR (126 MHz, CDCl_3_) *δ*_C(ppm)_ (Ordered C1-C17; C1′-C3′) 173.9, 33.7, 29.2–28.6, 31.4, 24.4, 22.2, 13.7. 64.7, 69.8, 62.8. EI-MS, M^+^ *m*/*z* 484 Calculated *m*/*z* 484.49, C_30_H_60_O_4_ (Figures S15–S17).

***(3S,6E,10E,14E,18E,22S)-2,6,10,15,19,23-hexamethyltetracosa-6,10,14,18-tetraene-1,2,3, 22,23-pentaol (6)***: ^1^H NMR (400 MHz, Methanol-*d*_4_) *δ*_H(ppm)_ 5.22–5.10 (m, 2H), 3.54–3.41 (m, 1H), 3.23 (dd, *J* = 10.6, 1.8 Hz, 1H), 2.25 (td, *J* = 10.1, 4.0 Hz, 1H), 2.14–1.94 (m, 14H), 1.71–1.57 (m, 1H), 1.61 (d, *J* = 1.3 Hz, 3H), 1.60 (d, *J* = 1.4 Hz, 3H), 1.49–1.26 (m, 3H), 1.12 (s, 3H), 1.10 (s, 3H). ^13^C NMR (100 MHz, Methanol-*d*_4_) *δ*_C(ppm)_ (Ordered C1-C30) 66.7, 73.7, 74.0, 29.3, 36.4, 135.0, 123.9, 26.2, 36.6, 135.0, 123.9, 27.7, 27.7, 123.8, 134.5, 36.4, 26.1, 123.8, 134.4, 36.4, 29.5, 77.1, 71.6, 25.9, 20.8, 15.9, 15.9, 15.8, 15.8, 24.7. HR-ESI, [M + H]^+^ *m*/*z* 495.4042, Calculated *m*/*z* 495.4049, [M + Na]^+^ *m*/*z* 517,3864, Calculated *m*/*z* 517,3869, C_30_H_54_O_5_Na (Figures S18–S20).

***(3S,6E,10E,14E,18E,22S)-2,6,10,15,19,23-hexamethyltetracosa-6,10,14,18-tetraene-2,3,22, 23-tetraol (7)***: ^1^H NMR (400 MHz, Methanol-*d*_4_) *δ*_H(ppm)_ 5.21–5.10 (m, 2H), 3.23 (dd, *J* = 10.6, 1.8 Hz, 1H), 2.30–2.19 (m, 1H), 2.14–2.04 (m, 1H), 2.05–1.94 (m, 4H), 1.75–1.62 (m, 1H), 1.62–1.61 (m, 3H), 1.60 (s, 3H), 1.41–1.26 (m, 1H), 1.15 (s, 3H), 1.12 (s, 3H). ^13^C NMR (100 MHz, Methanol-*d*_4_) *δ*_C(ppm)_ (Ordered C1-C30) 24.6, 71.5, 77.1, 29.5, 36.6, 134.5, 123.9, 39.3, 135.1, 123.3, 27.7, 27.7, 123.3, 134.5, 39.2, 26.1, 123.9, 135.1, 36.6, 29.5, 77.1, 71.5, 26.1, 15.9, 15.8, 15.8, 15.9, 26.1. HR-ESI, [M + H]^+^ *m*/*z* 479.4087, Calculated *m*/*z* 479,4100, C_30_H_55_O_4_, [M + Na]^+^ *m*/*z* 501.3909, Calculated *m*/*z* 501,3920, C_30_H_54_O_4_Na (Figures S21–S23).

***Mixture in the ratio 1:1 of β-sitosterol (8) and stigmasterol (9)***: ^1^H NMR (400 MHz, Chloroform-*d*) *δ*_H(ppm)_ 5.37–5.30 (m, 2H), 5.13 (dd, *J* = 15.1, 8.5 Hz, 1H), 5.00 (dd, *J* = 15.2, 8.7 Hz, 1H), 3.50 (brd, *J* = 4.6 Hz, 2H), 2.32–2.15 (m, 4H), 1.98 (s, 4H), 2.00–1.91 (m, 2H), 1.88–1.78 (m, 6H), 1.64 (td, *J* = 6.8, 4.8 Hz, 2H), 1.53 (s, 14H), 1.48 (d, *J* = 11.9 Hz, 3H), 1.41 (t, *J* = 5.4 Hz, 2H), 1.30–1.19 (m, 2H), 1.17–1.03 (m, 3H), 0.99 (s, 3H), 0.90 (d, *J* = 6.6 Hz, 3H), 0.87–0.72 (m, 9H), 0.67 (d, *J* = 7.3 Hz, 3H). ^13^C NMR (100 MHz, CDCl_3_) *δ*_C(ppm)_ (***Stigmasterol***, Ordered C1-C29) 37.7, 29.7, 71.8, 34.4, 140.7, 121.7, 31.6, 30.0, 50.1, 34.4, 23.7, 39.2, 41.3, 56.9, 23.0, 28.7, 56.7, 11.9, 18.3, 40.5, 21.5, 138.3, 129.3, 50.1, 32.8, 21.4, 19.3, 25.4, 11.9. *δ*_C(ppm)_ (***β-sitosterol***, Ordered C1-C29) 37.7, 29.7, 71.8, 34.4, 140.7, 121.7, 31.6, 30.0, 50.1, 34.4, 23.7, 39.2, 41.3, 56.9, 23.0, 28.7, 56.7, 11.9, 18.3, 40.5, 21.5, 47.0, 27.0, 50.1, 32.8, 21.4, 19.3, 25.4, 11.9 (Figures S24 and S25).

***Spinasterol (10)***: *δ*_H(ppm)_ 5.21 (dd, *J* = 15.1, 8.8 Hz, 1H), 5.19–5.15 (m, 1H), 5.09 (dd, *J* = 15.1, 8.8 Hz, 1H), 3.52–3.46 (m, 2H), 2.11–2.05 (m, 1H), 1.86 (d, *J* = 11.2 Hz, 1H), 1.83–1.71 (m, 4H), 1.69–1.61 (m, 2H), 1.60–1.48 (m, 2H), 1.43 (td, *J* = 12.1, 6.2 Hz, 1H), 1.37 (d, *J* = 12.1 Hz, 1H), 1.36–1.31 (m, 1H), 1.31 (s, 1H), 1.31–1.26 (m, 1H), 1.26–1.15 (m, 1H), 1.06 (d, *J* = 6.7 Hz, 2H), 0.87 (d, *J* = 6.3 Hz, 3H), 0.84–0.80 (m, 7H), 0.59 (s, 3H). ^13^C NMR (175 MHz, Acetone-*d*_6_) *δ*_C(ppm)_ (Ordered C1-C29) 38.0, 30.5, 70.7, 34.4, 40.8, 29.5, 118.4, 140.2, 50.4, 32.7, 21.7, 39.0, 41.1, 55.9, 23.7, 29.7, 56.6, 12.6, 13.3, 40.5, 21.5, 139.2, 130.2, 51.1, 31.8, 21.4, 19.3, 25.4, 12.4 (Figures S26 and S27).

**Mixture in the ratio 2:1:1 of glucoside of β-sitosterol (11), glucoside of stigmasterol (12), and glucoside of spinasterol (13)**: ^1^H NMR (600 MHz, DMSO-d_6_) δ_H(ppm)_ 5.35–5.26 (m, 3H), 5.15–5.07 (m, 1H), 5.01–4.94 (m, 1H), 4.84 (d, *J* = 4.7 Hz, 1H), 4.82–4.72 (m, 2H), 4.32–4.28 (m, 1H), 4.28–4.21 (m, 1H), 3.70–3.64 (m, 1H), 3.61–3.46 (m, 1H), 3.21–3.05 (m, 2H), 3.00–2.93 (m, 1H), 2.02–1.92 (m, 1H), 1.81–1.74 (m, 2H), 1.76–1.62 (m, 1H), 1.50 (pt, *J* = 7.5, 6.2, 4.7 Hz, 2H), 1.45–1.35 (m, 1H), 1.38–1.18 (m, 11H), 1.18–1.08 (m, 1H), 1.06–0.96 (m, 6H), 0.95 (s, 3H), 0.91–0.72 (m, 11H), 0.68–0.59 (m, 3H), 0.50 (s, 3H). ^13^C NMR (150 MHz, DMSO-d_6_) δ_C(ppm)_ (**glucoside of spinasterol**, Ordered C1-C29; C1′-C6′) 37.0, 29.7, 77.2, 34.4, 40.8, 29.5, 117.5, 139.2, 49.1, 34.4, 21.7, 39.2, 43.3, 54.9, 23.0, 28.7, 55.6, 12.4, 13.3, 40.5, 21.5, 138.9, 129.4, 51.1, 31.8, 21.4, 19.3, 25.4, 12.6; 101.2, 73.9, 76.9, 70.5, 76.7, 61.5. δ_C(ppm)_ (**glucoside of stigmasterol**, Ordered C1-C29; C1′-C6′) 37.1, 29.7, 77.2, 34.4, 140.6, 121.6, 31.6, 30.1, 49.1, 34.4, 23.7, 39.2, 41.3, 55.9, 23.1, 28.7, 55.6, 12.4, 18.3, 40.5, 21.5, 138.3, 129.3, 51.1, 32.8, 21.4, 19.3, 25.4, 12.6; 101.2, 73.9, 76.9, 70.5, 76.7, 61.5. δ_C(ppm)_ (**glucoside of β-sitosterol**, Ordered C1-C29; C1′-C6′) 37.0, 29.7, 76.2, 43.4, 140.6, 121.6, 31.6, 30.0, 49.1, 34.4, 23.7, 39.2, 41.3, 55.9, 23.0, 28.7, 55.6, 12.4, 18.3, 40.5, 21.5, 47.0, 27.0, 12.9, 30.8, 21.4, 20.3, 20.4, 12.6; 101.2, 73.9, 76.9, 70.5, 76.7, 61.5 (Figures S28 and S29).

***Lupenone (14)***: ^1^H NMR (600 MHz, CDCl_3_) *δ*_H(ppm)_ 4.74–4.68 (m, 2H), 2.53 (dddd, *J* = 15.5, 10.2, 7.4, 1.2 Hz, 1H), 2.42 (dddd, *J* = 15.7, 7.5, 4.3, 1.2 Hz, 1H), 2.27 (td, *J* = 11.1, 7.2 Hz, 1H), 1.99–1.82 (m, 2H), 1.70 (s, 1H), 1.58–1.43 (m, 3H), 1.45–1.36 (m, 1H), 1.36–1.30 (m, 1H), 1.29–1.16 (m, 3H), 1.10 (d, *J* = 1.3 Hz, 3H), 1.09–1.03 (m, 6H), 0.97 (d, *J* = 8.8 Hz, 6H), 0.71 (s, 3H). ^13^C NMR (150 MHz, CDCl_3_) *δ*_C(ppm)_ (Ordered C1-C30) 39.6, 34.2, 218.2, 47.9, 54.9, 19.8, 32.7, 40.2, 48.8, 36.9, 21.6, 26.6, 41.8, 42.3, 27.4, 32.7, 44.2, 53.9, 47.4, 148.2, 28.1, 40.7, 23.9, 20.9, 15.8, 16.5, 15.2, 16.5, 109.5, 19.7 (Figures S30 and S31).

***Taraxerol (15)***: ^1^H NMR (500 MHz, CDCl_3_) *δ*_H(ppm)_ 5.57 (dd, *J* = 8.2, 3.2 Hz, 1H), 3.23 (dd, *J* = 11.5, 4.6 Hz, 1H), 2.07 (dt, *J* = 12.8, 3.2 Hz, 1H), 1.95 (dd, *J* = 14.7, 3.2 Hz, 1H), 1.73–1.62 (m, 5H), 1.62–1.43 (m, 3H), 1.46–1.23 (m, 5H), 1.12 (s, 3H), 1.07–1.03 (m, 1H), 1.01 (s, 3H), 0.98 (s, 3H), 0.96 (s, 3H), 0.94 (d, *J* = 1.9 Hz, 6H), 0.85 (s, 3H), 0.84 (s, 3H), 0.81 (d, *J* = 2.4 Hz, 1H). ^13^C NMR (125 MHz, CDCl_3_) *δ*_C(ppm)_ (Ordered C1-C30) 37.7, 27.1, 79.0, 38.9, 55.5, 18.8, 35.1, 38.7, 48.7, 35.8, 17.5, 37.7, 37.5, 158.1, 116.8, 36.6, 37.9, 49.2, 41.3, 28.8, 33.6, 33.0, 29.8, 15.4, 15.4, 29.9, 25.9, 28.0, 33.3, 21.3 (Figures S32 and S33).

***Friedelane-2α,3α,28α-triol or pristriol (16)***: ^1^H NMR (600 MHz, Pyridine-*d*_5_) *δ*_H(ppm)_ 6.06 (2-OH, s, 1H), 5.93 (3-OH, s, 1H), 5.91 (2-OH, s, 1H), 4.53 (q, *J* = 2.9 Hz, 1H), 4.11 (s, 1H), 3.92 (dd, *J* = 10.9, 4.5 Hz, 1H), 3.86 (dd, *J* = 10.9, 4.6 Hz, 1H), 2.30 (ddd, *J* = 14.4, 9.3, 5.8 Hz, 1H), 2.23 (tt, *J* = 13.1, 2.5 Hz, 1H), 2.10 (d, *J* = 7.5 Hz, 1H), 2.01 (d, *J* = 12.8 Hz, 1H), 1.97 (d, *J* = 2.0 Hz, 1H), 1.91 (d, *J* = 13.5 Hz, 1H), 1.87–1.78 (m, 2H), 1.71 (ddd, *J* = 16.6, 9.3, 3.1 Hz, 1H), 1.53–1.37 (m, 11H), 1.31 (s, 3H), 1.22 (d, *J* = 7.1 Hz, 3H), 1.19 (s, 3H), 1.06 (s, 3H), 1.02 (s, 3H), 0.99 (s, 3H), 0.96 (s, 3H). ^13^C NMR (150 MHz, Pyridine-*d*_5_) *δ*_C(ppm)_ (Ordered C1-C30) 24.5, 71.8, 76.4, 35.6, 44.2, 39.4, 17.8, 52.5, 38.3, 52.9, 34.6, 30.3, 42.1, 39.6, 31.5, 28.0, 35.4, 38.3, 36.8, 29.7, 32.1, 33.6, 11.8, 16.5, 18.6, 19.2, 32.9, 67.3, 18.8, 34.1 (Figures S34 and S35).

***7,4′-dimethyl-3′-hydroxygenistein (17)***: ^1^H NMR (600 MHz, Methanol-*d*_4_) *δ*_H(ppm)_ 8.00 (s, 3H), 7.15 (d, *J* = 2.0 Hz, 1H), 6.93 (dd, *J* = 8.1, 2.0 Hz, 1H), 6.84 (d, *J* = 8.1 Hz, 1H), 6.46–6.42 (m, 2H), 3.90 (s, 3H), 3.89 (s, 3H). ^13^C NMR (150 MHz, Methanol-*d*_4_) *δ*_C(ppm)_ (Ordered C2-C10; C1′-C6′), 151.3, 125.5, 176.4, 107.9, 161.6, 96.1, 163.5, 94.8, 160.0; 123.6, 112.6, 146.3, 147.2, 114.9, 121.6, 55.0 (7-OMe), 55.0 (4′-OMe) (Figures S36 and S37).

### 4.5. Antidiabetic Assays

#### 4.5.1. In Vitro α-Amylase Inhibitory Assay

The *α*-amylase inhibitory effect of the crude extracts and isolated compounds was assessed using the 3,5-dinitrosalicylic acid (DNS) method, following established procedures [65,66]. Briefly, 500 µL of the test samples at different concentrations (100–500 µg/mL) were mixed with 500 µL of α-amylase solution (0.5 mg/mL; porcine pancreatic α-amylase, Type VI-B, Sigma-Aldrich, A3176). The mixture was supplemented with 100 µL of phosphate buffer (pH 6.9; 0.2 mM) and 1% starch solution as the substrate. The reaction mixture was incubated at 37 °C for 5 min. The reaction was then terminated by adding 2 mL of DNS reagent. The tubes were immersed in a boiling water bath (100 °C) for 15 min to develop the color. After cooling in an ice bath, the mixture was diluted with 10 mL of distilled water. The absorbance was measured at 540 nm using a spectrophotometer. Acarbose was used as the standard reference drug.

#### 4.5.2. In Vitro α-Glucosidase Inhibitory Assay

The *α*-glucosidase inhibition was evaluated following the method described by Vennila and Pavithra [67]. A volume of 1 mL of starch solution (2% *w*/*v* maltose) was incubated with Tris buffer (pH 8; 0.2 M) and 1 mL of test samples at concentrations (100–500 µg/mL). The mixture was pre-incubated at 37 °C for 10 min. The enzymatic reaction was initiated upon the addition of 1 mL of α-glucosidase (1 U/mL; from Saccharomyces cerevisiae, Type I, Sigma-Aldrich, G5003) and was subsequently incubated at 35 °C for 40 min. The reaction was stopped by adding 2 mL of HCl (6 N). The absorbance was measured at 540 nm. Acarbose was used as the standard reference drug.

### 4.6. In Vitro Antioxidant Activities

The antioxidant potential of the *P. dulce* extract and its isolated compounds was evaluated using 1,1-diphenyl-2-picrylhydrazyl (DPPH), 2,2′-azino-bis-3-ethylbenzylthiazoline-6-sulfonic acid (ABTS), and Ferric Reducing Antioxidant Power (FRAP) assays by determining their half-maximal inhibitory concentration (IC_50_) values.

#### 4.6.1. Determination of the Antiradical Activity DPPH

The antioxidant activity of the sample (25, 50, 100, 150 and 200 μg/mL) prepared in methanol solution was measured in terms of radical-scavenging capacity, according to the DPPH method [68]. A volume of 50 µL of extract was added to 1.95 mL of a methanolic DPPH solution (0.3 mM) and maintained in the dark for 30 min. Control experiments without the sample but containing equivalent quantities of methanol were carried out in the same conditions. The absorbance was subsequently measured spectrophotometrically at 517 nm. Ascorbic acid was used as the reference.

#### 4.6.2. Determination of the Antiradical Activity ABTS

The ABTS solution was prepared by mixing ABTS (8 mM) with potassium persulfate (3 mM) in 25 mL of distilled water. The solution was maintained at room temperature in the dark for 16 h before use [69] with slight modifications. The ABTS solution was diluted 10 times with 95% ethanol. A volume of 500 µL of the sample (25–200 µg/mL) was added to 1 mL of diluted ABTS solution and maintained at room temperature for 30 min. Control experiments without the sample, but containing equivalent quantities of distilled water, were carried out in the same conditions. The absorbance was subsequently measured spectrophotometrically at 734 nm after 30 min. Ascorbic acid was used as the reference. The percentage inhibition of these assays was calculated according to the following formula:

Inhibition (%) = [(Abs control − Abs sample)/Abs control] × 100

Inhibition Concentration 50 (IC_50_ parameter was used for the interpretation of the results [49]. The discoloration of the sample was plotted against its concentration in order to calculate the IC_50_ value.

#### 4.6.3. Evaluation of the Ferric-Reducing Antioxidant Power (FRAP)

The ferric reducing antioxidant power was assessed using the method by Benzie and Strain [4,70] with adjustments. Briefly, 100 µL of sample solution at different concentrations [25–400 µM] was added to 3 mL of the pre-warmed FRAP reagent. The mixture was vortexed thoroughly and then incubated in a water bath at 37 °C for 30 min in the dark. The absorbance was measured spectrophotometrically at 593 nm. The Effective Concentration (EC_50_) was measured, utilizing ascorbic acid as a reference. The EC_50_ value represents the concentration of the sample (in µM) necessary to attain 50% of its maximum reducing capability.

### 4.7. Statistical Analysis

Data were entered and calculations performed using Excel 2013 during this study. IC_50_ and EC_50_ values of the various compounds were compared to the standard (ascorbic acid/acarbose) utilizing one-way ANOVA, followed by Tukey’s multiple comparison test. Differences were deemed significant at *p* < 0.05. Graphs and analyses were conducted utilizing GraphPad Prism 8.0.1.

### 4.8. Computational Investigations

Compounds from *P. dulce* (Fabaceae) were analyzed theoretically for their ability to inhibit α glucosidase and α amylase (Table 1, Table 2, Table 3 and Tables S1–S4), with the aim of advancing knowledge on the antidiabetic properties of this plant species. The online server Swiss ADME (https://www.swissadme.ch// accessed on 27 December 2025 at 13:42 p.m.) performed computational Absorption, Distribution, Metabolism, and Excretion (ADME) profiling to supplement these studies. ADME criteria such physicochemical qualities, water solubility, pharmacokinetics, drug resemblance, lipophilicity, and medicinal chemistry descriptors, were analyzed for compounds **1–7** and **11–13** (Table 4, Tables S5 and S6).

Furthermore, molecular docking simulations were carried out for all isolated compounds (**1**–**17**) to explore their interactions with human pancreatic *α*-amylase and human maltase-glucoamylase. From the Protein Data Bank (PDB), the investigated protein structures were retrieved: the crystal structure of the N-terminal subunit of human maltase-glucoamylase, with PDB ID: 2QLY, and the human pancreatic *α*-amylase in complex with myricetin, with PDB ID: 4GQR. Protein preparation included removal of water molecules and co-crystallized ligands, addition of hydrogen atoms, and energy minimization using the Amber14:EHT force field with a gradient of 0.01 kcal·mol^−1^·Å^−1^. Protein preparations were achieved using MOE-2019 (Molecular Operating Environment), following established protocols [71,72], and saved in the *.pdb* format. Ligands were generated in ChemDraw Ultra (v12.0.2.1076, CambridgeSoft), saved in *.sdf* format, and subsequently prepared in MOE-2019 by energy minimization (MMFF94x force field) to obtain optimized three-dimensional (3D) structures. Docking was performed using the Triangular Matcher placement method combined with an induced-fit protocol (side chain flexibility within 5 Å of the binding site). The GBVI/WSA dG scoring function was used for binding energy estimation. To validate the docking protocol, acarbose was re-docked into both targets; the root-mean-square deviation (RMSD) between the predicted and crystallographic poses was 1.42 Å for 4GQR and 1.58 Å for 2QLY, confirming acceptable reproducibility. Binding energies are reported in kcal/mol (Table 1). A threshold of ≤ –7.00 kcal/mol was considered indicative of strong binding [7,29,30]. Post-docking analyses included the generation of 2D and 3D interaction maps, enabling detailed visualization of ligand–protein interactions and the involvement of specific amino-acid residues (Table 2, Table 3, Tables S2 and S4).

## 5. Conclusions

This study demonstrates that root extracts of *P. dulce*, along with the isolated compounds (**1**–**17**), possess notable antidiabetic and antioxidant activities. The *n*-hexane extract (PDEH) and the triterpenoid pristriol (**16**) exhibited the strongest free radical-scavenging capacity and ferric-reducing power. In contrast, taraxerol (**15**), together with the two newly reported compounds (**1**–**2**), showed superior inhibitory activity against α-amylase and α-glucosidase, in some cases exceeding that of the reference drug acarbose. Importantly, molecular docking analyses targeting human α-glucosidase (2QLY) and human α-amylase (4GQR) suggest that compounds **1**, **2**, **6**, and **7** may interact favorably with these therapeutic targets, exhibiting predicted binding affinities comparable to those of acarbose and supporting their potential as enzyme inhibitors. These promising in vitro antidiabetic findings were further supported by molecular docking analyses, where compounds **1**–**2** emerged as the most favorable candidates for α-amylase and α-glucosidase inhibition, highlighting their potential as lead structures for future drug development. At this stage of investigation, these results provide scientific support for the traditional use of *P. dulce* root bark as an antidiabetic remedy. However, validation using human recombinant α-glucosidase, in vivo efficacy studies in diabetic animal models, and experimental ADME profiling are essential prerequisites before clinical translation. Further studies are required to validate these effects in vivo, elucidate the underlying molecular mechanisms, and develop standardized phytopharmaceutical formulations derived from this plant.

## Figures and Tables

**Table 1:** Binding affinity of compounds (**1**–**17**) with 2QLY and 4GQR.

Ligands	Binding Affinity (kcal/mol)		Ligands	Binding Affinity (kcal/mol)	
	*α*-Glucosidase (2QLY)	*α*-Amylase (4GQR)		*α*-Glucosidase (2QLY)	*α*-Amylase (4GQR)
**1**	−10.35	−9.08	**10**	−6.33	−7.86
**2**	−9.79	−8.34	**11**	−7.33	−9.63
**3**	−10.58	−8.52	**12**	−8.08	−9.15
**4**	−10.58	−8.06	**13**	−6.85	−9.35
**5**	−10.20	−9.34	**14**	−2.31	−7.45
**6**	−10.75	−9.64	**15**	+1.00	−7.22
**7**	−9.93	−9.50	**16**	−0.99	−7.86
**8**	−6.98	−7.73	**17**	−7.78	−6.41
**9**	−5.75	−7.98	**Acarbose**	−9.42	−9.70

**Table 2:** 2D and 3D interactions with 2QLY for *α*-glucosidase.

Ligands	2D	3D
**1**	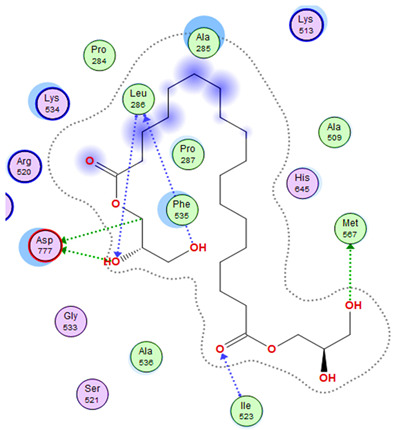	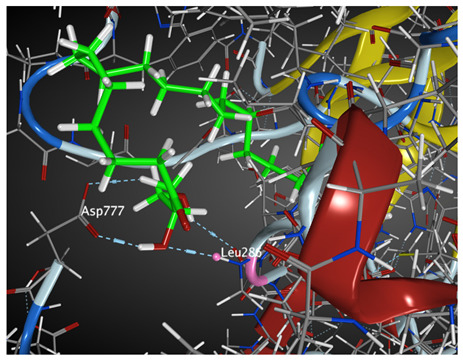
**2**	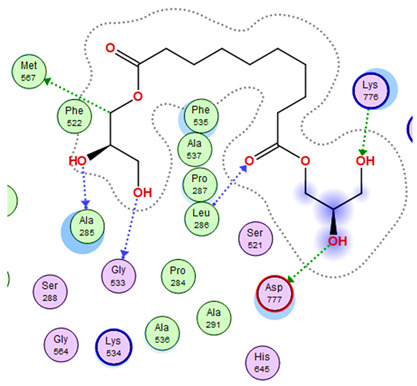	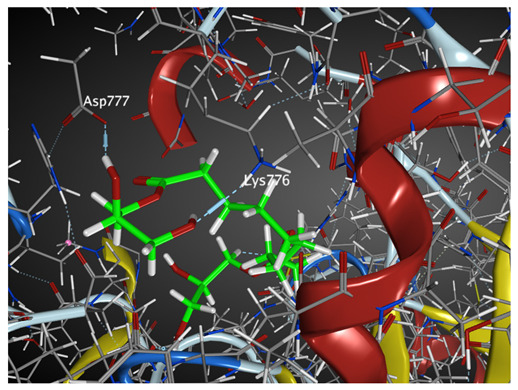
**4**	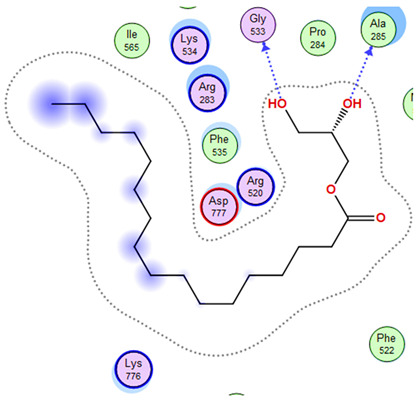	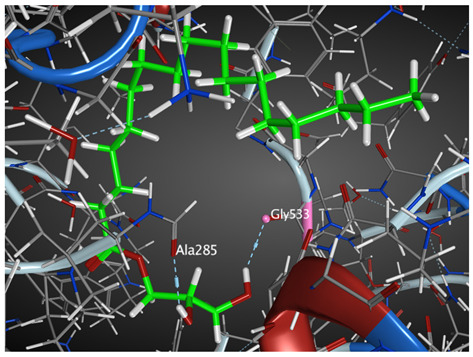

**Table 3:** 2D and 3D interactions with 4GQR for *α*-amylase.

Ligands	2D	3D
**1**	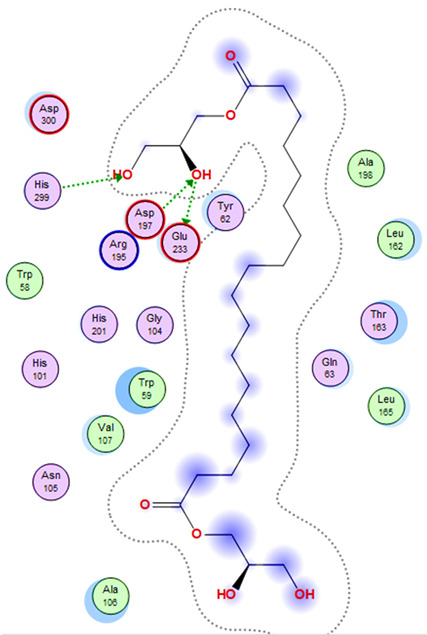	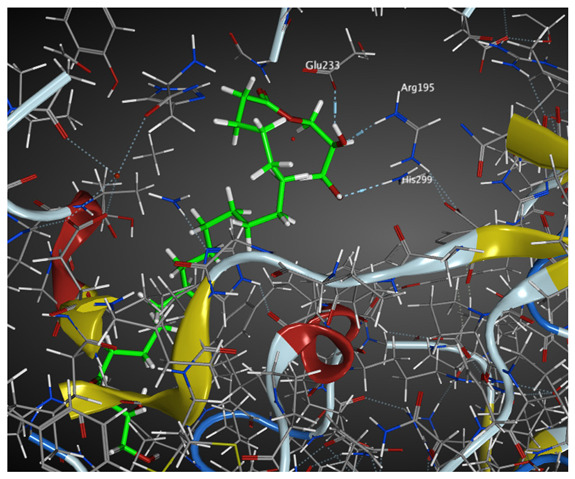
**2**	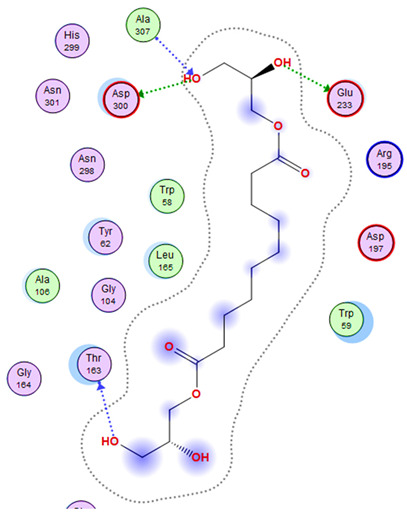	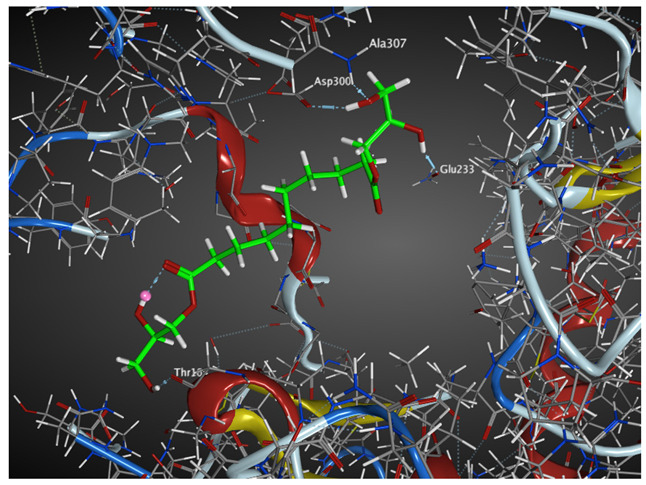
**4**	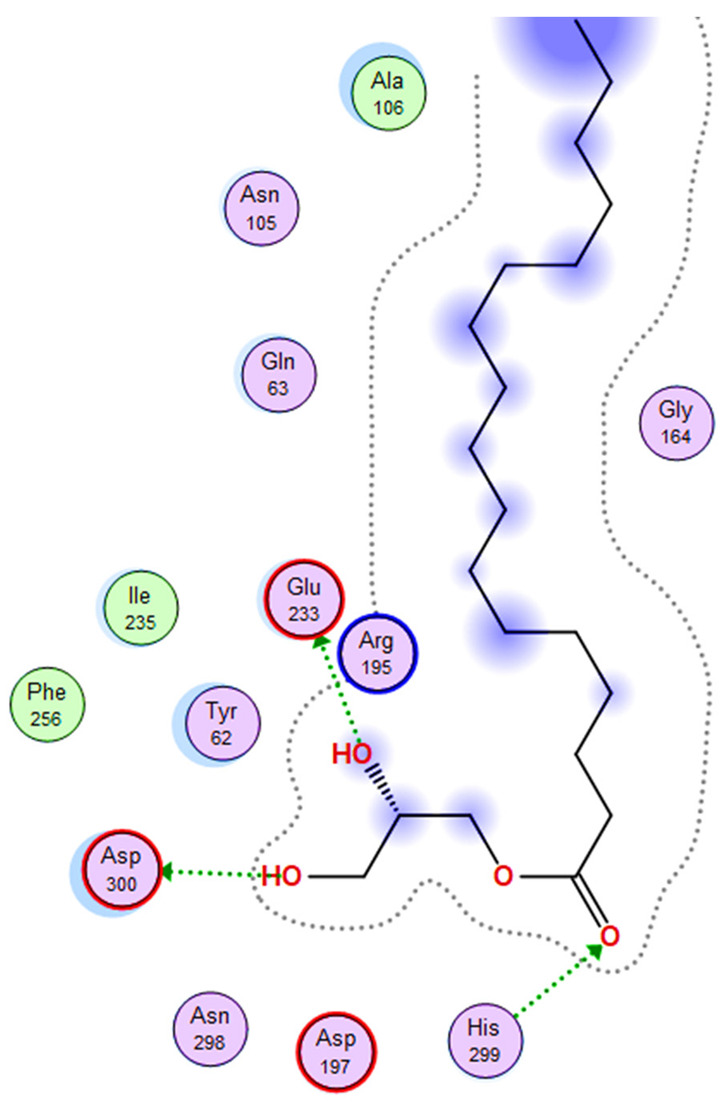	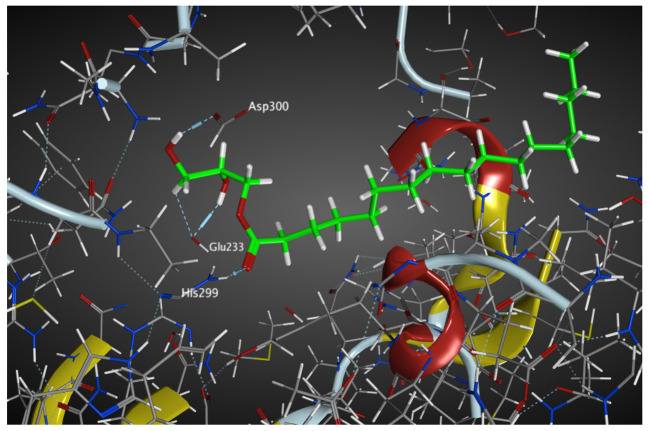

**Table 4:** ADME main properties of compounds **1**–**7** and **11**–**13**.

**Pharmacokinetics**										
Compounds	**1**	**2**	**3**	**4**	**5**	**6**	**7**	**11**	**12**	**13**
GI absorption	High	High	Low	High	Low	Low	Low	Low	High	High
BBB permeant	No	No	No	Yes	No	No	No	No	No	No
P-gp substrate	Yes	No	No	Yes	No	No	No	No	Yes	Yes
CYP1A2 inhibitor	No	No	Yes	Yes	No	No	No	No	No	No
CYP2C19 inhibitor	Yes	No	No	No	No	No	No	No	No	No
CYP2C9 inhibitor	No	No	No	No	No	No	No	No	No	No
CYP2D6 inhibitor	No	No	No	Yes	No	No	No	No	No	No
CYP3A4 inhibitor	No	No	No	No	No	No	No	No	No	No
Log *K*_p_ (skin permeation)	−5.94 cm/s	−8.33 cm/s	−0.11 cm/s	−3.52 cm/s	−0.53 cm/s	−0.53 cm/s	−0.53 cm/s	−4.32 cm/s	−4.86 cm/s	−5.04 cm/s
**Druglikeness**										
Lipinski	Yes; 0 violations	Yes; 0 violations	Yes; 1 violation: MLOGP > 4.15	Yes; 0 violations	Yes; 1 violation: MLOGP > 4.15	Yes; 1 violation: MLOGP > 4.15	Yes; 1 violation: MLOGP > 4.15	Yes; 1 violation: MW > 500	Yes; 1 violation: MW > 500	Yes; 1 violation: MW > 500
Bioavailability Score	0.55	0.55	0.85	0.55	0.55	0.55	0.55	0.55	0.55	0.55
**Medicinal Chemistry**										
**PAINS**	0 alert	0 alert	0 alert	0 alert	0 alert	0 alert	0 alert	0 alert	0 alert	0 alert
**Synthetic accessibility**	4.82	3.88	3.36	4.00	5.22	5.22	5.22	8.02	7.93	7.81
**Physicochemical Properties**										
TPSA	133.52 Å^2^	133.52 Å^2^	37.30 Å^2^	66.76 Å^2^	66.76 Å^2^	80.92 Å^2^	101.15 Å^2^	99.38 Å^2^	99.38 Å^2^	99.38 Å^2^
**Lipophilicity**										
Consensus Log *P*_o/w_	3.75	1.01	8.43	5.16	8.68	8.68	8.68	5.56	5.36	5.29

Gastrointestinal (GI), blood–brain barrier (BBB), cytochrome P450 (CYP), P-glycoprotein (P-gp), pan assay interference structures (PAINS), topological polar surface area (TPSA).

## Data Availability

Research data are available in the Supplementary Materials.

## Supplementary Materials

The following supporting information can be downloaded at: https://www.mdpi.com/article/10.3390/molecules31122166/s1, Figure S1: HR-ESI (+ve) spectrum of compound **1**; Figure S2: ^1^H NMR (500 MHz, Methanol-d4) spectrum of compound **1**; Figure S3: ^13^C NMR and DEPT-135 (125 MHz, Methanol-*d*_4_) spectra of compound **1**; Figure S4: COSY spectrum of compound **1**; Figure S5: HSQC spectrum of compound **1**; Figure S6: HMBC spectrum of compound **1**; Figure S7: HR-ESI (+ve) spectrum of compound **2**; Figure S8: ^1^H NMR (500 MHz, Methanol-*d*_4_) spectrum of compound **2**; Figure S9: ^13^C NMR (125 MHz, Methanol-*d*_4_) spectrum of compound **2**; Figure S10: IE-MS spectrum of compound **3**; Figure S11: ^1^H NMR (400 MHz, CDCl_3_) spectrum of compound **3**; Figure S12: HR-ESI (+ve) spectrum of compound **4**; Figure S13: ^1^H NMR (600 MHz, CDCl_3_) spectrum of compound **4**; Figure S14: ^13^C NMR (150 MHz, CDCl_3_) spectrum of compound **4**; Figure S15: LR-EI-MS spectrum of compound **5**; Figure S16: ^1^H NMR (600 MHz, CDCl_3_) spectrum of compound **5**; Figure S17: ^13^C NMR (150 MHz, CDCl_3_) spectrum of compound **5**; Figure S18: HR-ESI (+ve) spectrum of compound **6**; Figure S19: ^1^H NMR (400 MHz, Methanol-*d*_4_) spectrum of compound **6**; Figure S20: ^13^C NMR and DEPT-135 (100 MHz, Methanol-*d*_4_) spectra of compound **6**; Figure S21: HR-ESI (+ve) spectrum of compound **7**; Figure S22: ^1^H NMR (400 MHz, Methanol-*d*_4_) spectrum of compound **7**; Figure S23: ^13^C NMR (100 MHz, DMSO-*d*_6_) spectrum of compound 7; Figure S24: ^1^H NMR (400 MHz, CDCl_3_) spectrum of the mixture of compounds **8** and **9**; Figure S25: ^13^C NMR (100 MHz, CDCl_3_) spectrum of the mixture of compounds **8** and **9**; Figure S26: ^1^H NMR (700 MHz, Acetone-d6) spectrum compound **10**; Figure S27: ^13^C NMR (175 MHz, Acetone-*d*_6_) spectrum of compound **10**; Figure S28: ^1^H NMR (600 MHz, DMSO-d6) spectra of compounds **11**, **12**, and **13**; Figure S29: ^13^C NMR (150 MHz, DMSO-*d*_6_) spectra of compounds **11**, **12**, and **13**; Figure S30: ^1^H NMR (600 MHz, CDCl_3_) spectrum of compound **14**; Figure S31: ^13^C NMR (150 MHz, CDCl_3_) spectrum of compound **14**; Figure S32**:** ^1^H NMR (500 MHz, CDCl_3_) spectrum of compound **15**; Figure S33: ^13^C NMR (126 MHz, CDCl_3_) spectrum of compound **15**; Figure S34: ^1^H NMR (600 MHz, Pyridine-*d*_5_) spectrum of compound **16**; Figure S35: ^13^C NMR and DEPT-135 (150 MHz, Pyridine-*d*_5_) spectra of compound **16**; Figure S36: ^1^H NMR (600 MHz, Methanol-*d*_4_) spectrum of compound **17**; Figure S37: ^13^C NMR (150 MHz, Methanol-*d*_4_) spectrum of compound **17**. Table S1: Docking energies with **4GQR** for α-amylase and types of interaction involved; Table S2: 2D interactions with **4GQR** for α-amylase; Table S3: Docking energies with **2QLY** for α-glucosidase and types of interaction involved; Table S4: 2D interactions with **2QLY** for α-glucosidase; Table S5: Adsorption, Distribution, Metabolism and Excretion (ADME) properties of compounds **1**–**7** and **11**–**13**; Table S6: ADME Radar representations of compounds **1**–**7** and **11**–**13**.

## Abbreviations

The following abbreviations are used in this manuscript:

1D-NMR	1 Dimension Nuclear Magnetic Resonance
2D-NMR	2 Dimension Nuclear Magnetic Resonance
ABTS	2,2′-azinobis (3-ethylbenzothiazoline-6-sulfonic acid)
ADME	Absorption, Distribution, Metabolism, Excretion
BE	Binding energy
CC	Column Chromatography
DCM	Dichloromethane
DPPH	2,2-diphenyl-1-picrylhydrazyl
EC_50_	Effective Concentration 50%
EI-MS	Electronic Impact Mass Spectrometry
EtOAc	Ethyl acetate
FRAP	Ferric-reducing antioxidant power
HR-ESI	High Resolution ElectroSpray Ionization
IC_50_	Inhibitory concentration at 50%
MeOH	Methanol
MOE	Molecular Operating Environment
*P. dulce*	*Pithecellobium dulce*
SiO_2_	Silica
T2DM	Type 2 diabetes mellitus
TLC	Thin Layer Chromatography
TMS	Tetramethylsilane
UV	Ultraviolet
*α*-Amylase, PDB, Id: 4GQR	Human pancreatic alpha-amylase
*α*-Glucosidase, PDB, Id: 2QLY	Human maltase-glucoamylase
