# Supplementary material for: Antidiabetic and Antioxidant Potential of a New Bisglyceride Derivative Together with Other Compounds from the Root Bark of Pithecellobium dulce: In Vitro and In Silico Studies
Source: Molecules. 2026 Jun 19;31(12):2166. doi: 10.3390/molecules31122166 (PMC13304693; doi:10.3390/molecules31122166)
Supplement: Supplementary file 1 [file molecules-31-02166-s001.zip › molecules-4295200-supplementary.pdf]

Supplementary material

Antidiabetic and Antioxidant Potential of a New Bisglyceride Derivative Together With Other Compounds From The Root Bark Of *Pithecellobium dulce*: *In Vitro* and *In Silico* Studies

Gertrude Nembot Messah[1], Peron Bosco Leutcha[1,2,*], Gabrielle Ange Amang à Ngnoung[3], Guy Roussel Takuissu Nguemto[4], Brice Junior Edie Enang II[5], Hamadou Mamoudou[6,7], Soh Désiré[8], William Feudjou Fouatio[9], Alembert Tiabou Tchinda[9], Bienvenu Tsakem[10], Madan Poka[10], Patrick Hulisani Demana[10] , Mehmet Öztürk[2], Xavier Siwe Noundou[10,*], and Yves Oscar Nganso Ditchou[1,*]

Abstract

Type 2 diabetes mellitus (T2DM) is a global health challenge characterized by chronic hyperglycemia and oxidative stress. The stem *Pithecellobium dulce* has long been recognized for its antidiabetic potential; however, its specific bioactive constituents and mechanisms of action remain poorly defined. The DCM/MeOH extract of *P. dulce* stem bark was subjected to chromatographic purification and spectroscopic characterization, leading to seventeen compounds (**1**–**17**), including two bisglycerol derivatives: one unprecedented (**1**) and one reported for the first time from a natural source (**2**). Antioxidant evaluation revealed notable radical scavenging activity against DPPH (IC_{50} = 15.30 µg/mL) and ABTS (IC_{50} = 12.80 µg/mL) from the hexane extract, along with significant ferric reducing power (EC_{50} = 4200 µM $FeSO_4$/g) from pristriol (**16**). *In vitro* assays demonstrated inhibition of α-amylase (IC_{50} 53.88–112.24 µg/mL; Acarbose, IC_{50} 91.20 µg/mL) and α-glucosidase (IC_{50} 18.38–136.88 µg/mL; Acarbose, IC_{50} 11.31 µg/mL). Compounds **15**, **1**, and **2** exhibited greater effectiveness than the reference for α-amylase (IC_{50} 53.88, 88.15, and 92.62 µg/mL, respectively) and α-glucosidase (IC_{50} 18.38, 39.25, and 36.40 µg/mL, respectively). Docking studies supported these findings, revealing binding energies of -9.08, -8.34, and -7.22 kcal/mol for **1**, **2**, and **15** with α-amylase, and -10.35 and -9.79 kcal/mol for **1** and **2** with α-glucosidase. Furthermore, ADME profiling identified **1** and **2** as promising lead candidates for dual enzyme inhibition. Overall, *P. dulce* stem bark represents a potent source of bioactive molecules with both antioxidant and dual enzyme-inhibitory properties, thereby validating its traditional use and highlighting its potential in developing multi-target therapies for T2DM management.

Keywords: *Pithecellobium dulce*, Glycerol derivatives, Antidiabetic activity, Antioxidant activity, Molecular docking.

Contents

Figure S1: HR-ESI (+ve) spectrum of compound 1................ **Error! Bookmark not defined.**
Figure S2: ^{1}H NMR (500 MHz, Methanol-*d*4) spectrum of compound 1**Error! Bookmark not defined.**
Figure S3: ^{13}C NMR and DEPT-135 (125 MHz, Methanol-*d*4) spectra of compound 1 .**Error! Bookmark not defined.**
Figure S4: COSY spectrum of compound 1............................ **Error! Bookmark not defined.**
Figure S5: HSQC spectrum of compound 1............................ **Error! Bookmark not defined.**
Figure S6: HMBC spectrum of compound 1........................... **Error! Bookmark not defined.**
Figure S7: HR-ESI (+ve) spectrum of compound 2................ **Error! Bookmark not defined.**
Figure S8: ^{1}H NMR (500 MHz, Methanol-*d*4) spectrum of compound 2**Error! Bookmark not defined.**
Figure S9: ^{13}C NMR (125 MHz, Methanol-*d*4) spectrum of compound 2**Error! Bookmark not defined.**
Figure S10: IE-MS spectrum of compound 3.......................... **Error! Bookmark not defined.**
Figure S11: ^{1}H NMR (400 MHz, $CDCl_3$) spectrum of compound 3**Error! Bookmark not defined.**
Figure S12: HR-ESI (+ve) spectrum of compound 4............. **Error! Bookmark not defined.**
Figure S13: ^{1}H NMR (600 MHz, $CDCl_3$) spectrum of compound 4**Error! Bookmark not defined.**
Figure S14: ^{13}C NMR (150 MHz, $CDCl_3$) spectrum of compound 4**Error! Bookmark not defined.**
Figure S15: LR-EI-MS spectrum of compound 5.................. **Error! Bookmark not defined.**
Figure S16: ^{1}H NMR (600 MHz, $CDCl_3$) spectrum of compound 5**Error! Bookmark not defined.**
Figure S17: ^{13}C NMR (150 MHz, $CDCl_3$) spectrum of compound 5**Error! Bookmark not defined.**
Figure S18: HR-ESI (+ve) spectrum of compound 6............. **Error! Bookmark not defined.**
Figure S19: ^{1}H NMR (400 MHz, Methanol-*d*4) spectrum of compound 6**Error! Bookmark not defined.**
Figure S20: ^{13}C NMR and DEPT-135 (100 MHz, Methanol-*d*4) spectra of compound 6**Error! Bookmark not defined.**
Figure S21: HR-ESI (+ve) spectrum of compound 7............. **Error! Bookmark not defined.**
Figure S22: ^{1}H NMR (400 MHz, Methanol-*d*4) spectrum of compound 7**Error! Bookmark not defined.**
Figure S23: ^{13}C NMR (100 MHz, DMSO-*d*6) spectrum of compound 7**Error! Bookmark not defined.**
Figure S24: ^{1}H NMR (400 MHz, $CDCl_3$) spectrum of the mixture of compounds 8 and 9 .... **Error! Bookmark not defined.**
Figure S25: ^{13}C NMR (100 MHz, $CDCl_3$) spectrum of the mixture of compounds 8 and 9 .... **Error! Bookmark not defined.**
Figure S26: ^{1}H NMR (700 MHz, Acetone-*d*6) spectrum compound 10**Error! Bookmark not defined.**
Figure S27: ^{13}C NMR (175 MHz, Acetone-*d*6) spectrum of compound 10**Error! Bookmark not defined.**
Figure 28: ^{1}H NMR (600 MHz, DMSO-*d*6) spectra of compounds 11, 12, and 13 ....**Error! Bookmark not defined.**

Figure 29: ^{13}C NMR (150 MHz, DMSO-d_6) spectra of compounds 11, 12, and 13 .... **Error! Bookmark not defined.**
Figure S30: ^{1}H NMR (600 MHz, $CDCl_3$) spectrum of compound 14**Error! Bookmark not defined.**
Figure S31: ^{13}C NMR (150 MHz, $CDCl_3$) spectrum of compound 14**Error! Bookmark not defined.**
Figure S32: ^{1}H NMR (500 MHz, $CDCl_3$) spectrum of compound 15**Error! Bookmark not defined.**
Figure S33: ^{13}C NMR (126 MHz, $CDCl_3$) spectrum of compound 15**Error! Bookmark not defined.**
Figure S34: ^{1}H NMR (600 MHz, Pyridine-d_5) spectrum of compound 16**Error! Bookmark not defined.**
Figure S35: ^{13}C NMR and DEPT-135 (150 MHz, Pyridine-d_5) spectra of compound 16**Error! Bookmark not defined.**
Figure S36: ^{1}H NMR (600 MHz, Methanol-d_4) spectrum of compound 17**Error! Bookmark not defined.**
Figure S37: ^{13}C NMR (150 MHz, Methanol-d_4) spectrum of compound 17**Error! Bookmark not defined.**
Table S1: Docking energies with 4GQR for α-amylase and types of interaction involved .... **Error! Bookmark not defined.**
Table S2: 2D interactions with 4GQR for α-amylase .... **Error! Bookmark not defined.**
Table S3: Docking energies with 2QLY for α-glucosidase and types of interaction involved .... **Error! Bookmark not defined.**
Table S4: 2D interactions with 2QLY for α-glucosidase .... **Error! Bookmark not defined.**
Table S5: Adsorption, Distribution, Metabolism and Excretion (ADME) properties of compounds 1–7 and 11–13 .... **Error! Bookmark not defined.**
Table S6: ADME Radar representations of compounds 1–7 and 11–13**Error! Bookmark not defined.**

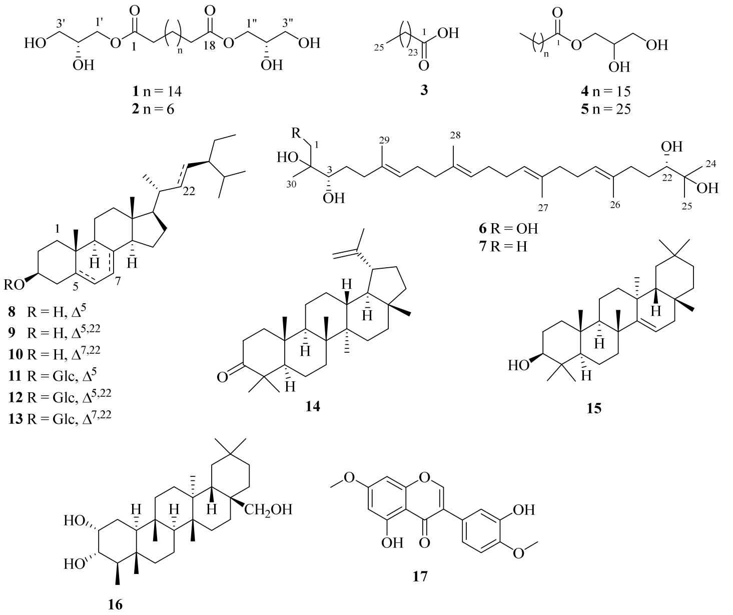

1-((2'*R*)-2',3'-dihydroxypropyl)-18-((2"*S*)-2",3"-dihydroxypropyl)octadecanedioate. (**1**), 1-((2'*R*)-2',3'-dihydroxypropyl)-10-((2"*S*)-2",3"-dihydroxypropyl)decanedioate (**2**), pentacosanoic acid (**3**), 1-heptadecanoylglycerol (**4**), heptacosanoate glycerol (**5**), (3*S*,6*E*,10*E*,14*E*,18*E*,22*S*)-2,6,10,15,19,23-hexamethyltetracosa-6,10,14,18-tetraene-1,2,3,22,23-pentaol (**6**), (3*S*,6*E*,10*E*,14*E*,18*E*,22*S*)-2,6,10,15,19,23-hexamethyltetracosa-6,10,14,18-tetraene-2,3,22,23-tetraol (**7**), β-sitosterol (**8**), stigmasterol (**9**), spinasterol (**10**), glucoside of β-sitosterol (**11**), glucoside of stigmasterol (**12**), glucoside of spinasterol (**13**), lupenone (**14**), taraxerol (**15**), friedelane-2α,3α,28α-triol ou pristriol (**16**), 7,4'-dimethyl-3'-hydroxygenistein (**17**)

Compounds isolated and characterized (**1–17**)

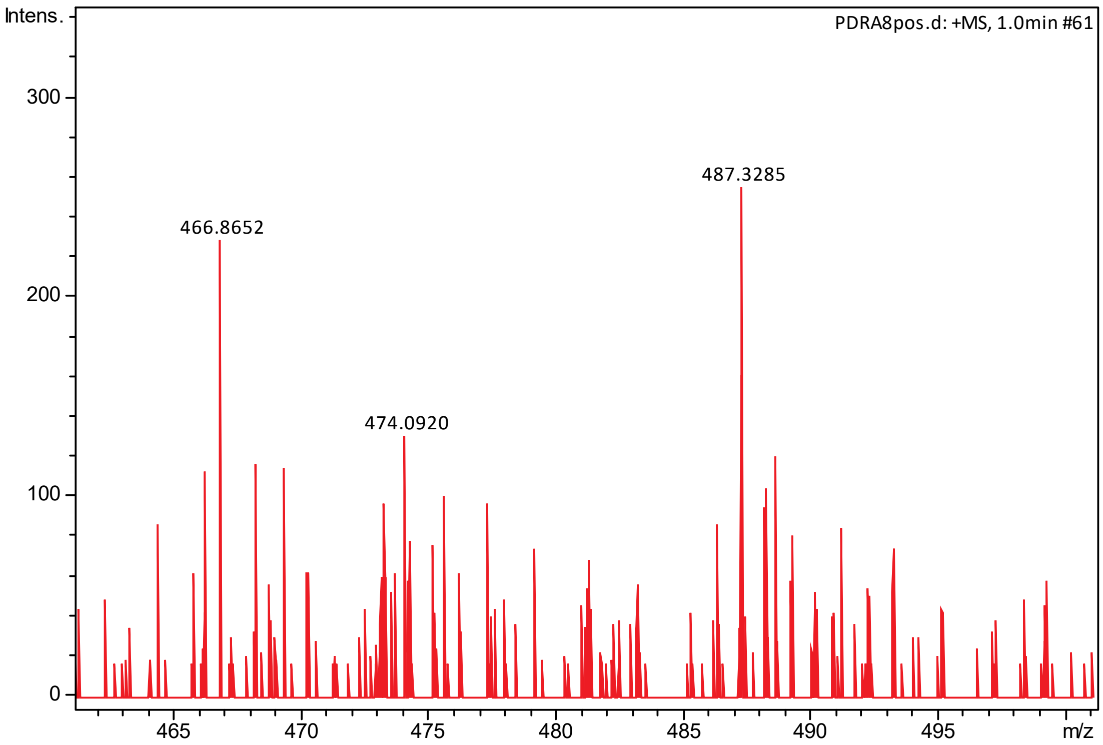

Figure S1: HR-ESI (+ve) spectrum of compound **1**

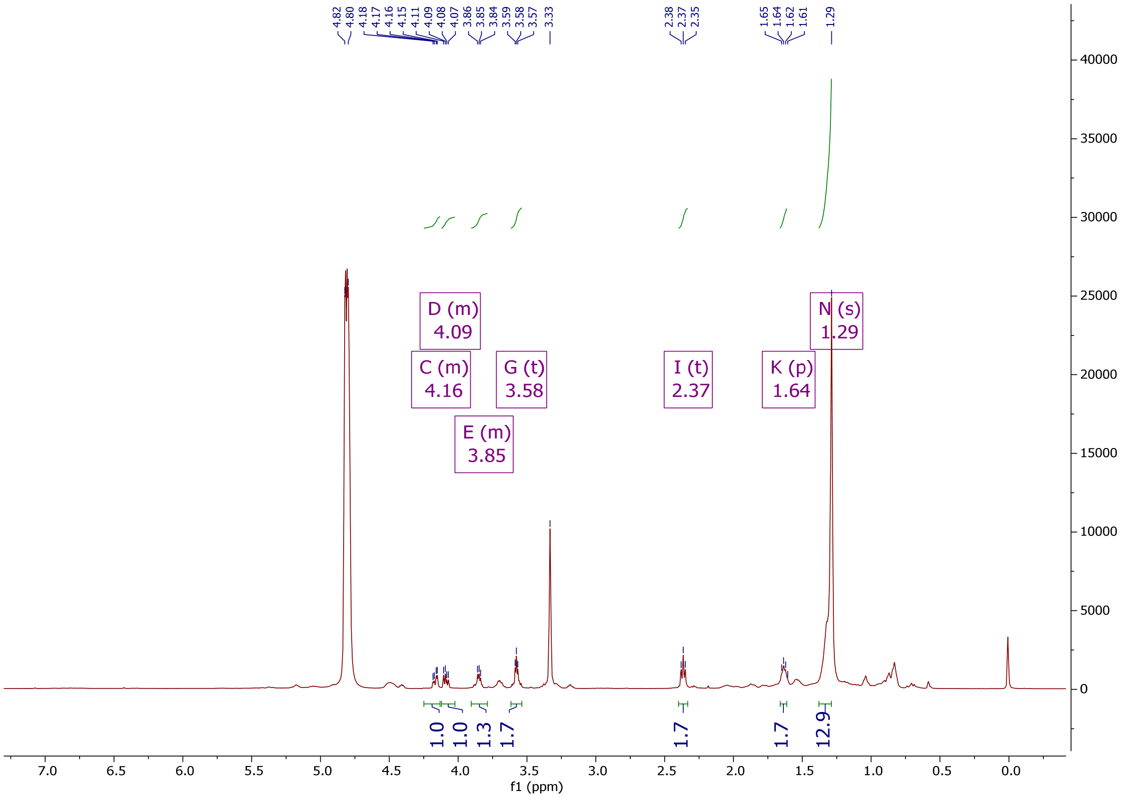

Figure S2: ^{1}H NMR (500 MHz, Methanol-d_4) spectrum of compound **1**

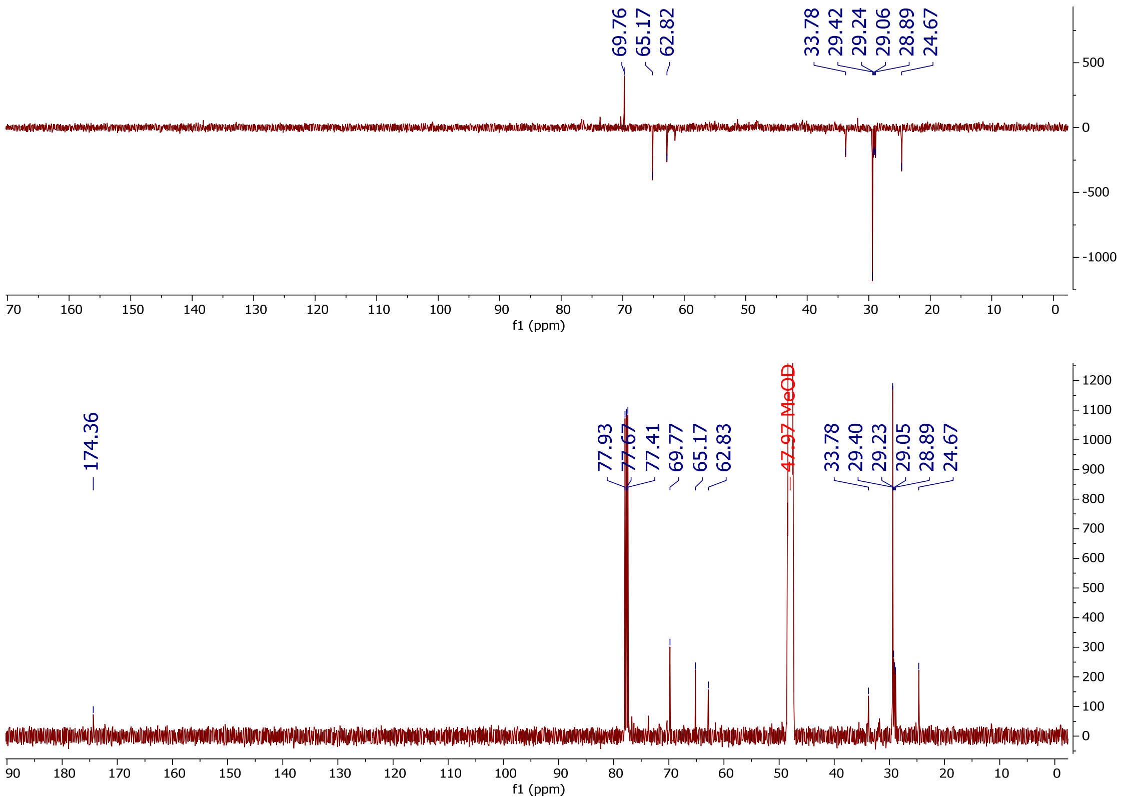

Figure S3: ^{13}C NMR and DEPT-135 (125 MHz, Methanol-d_4) spectra of compound **1**

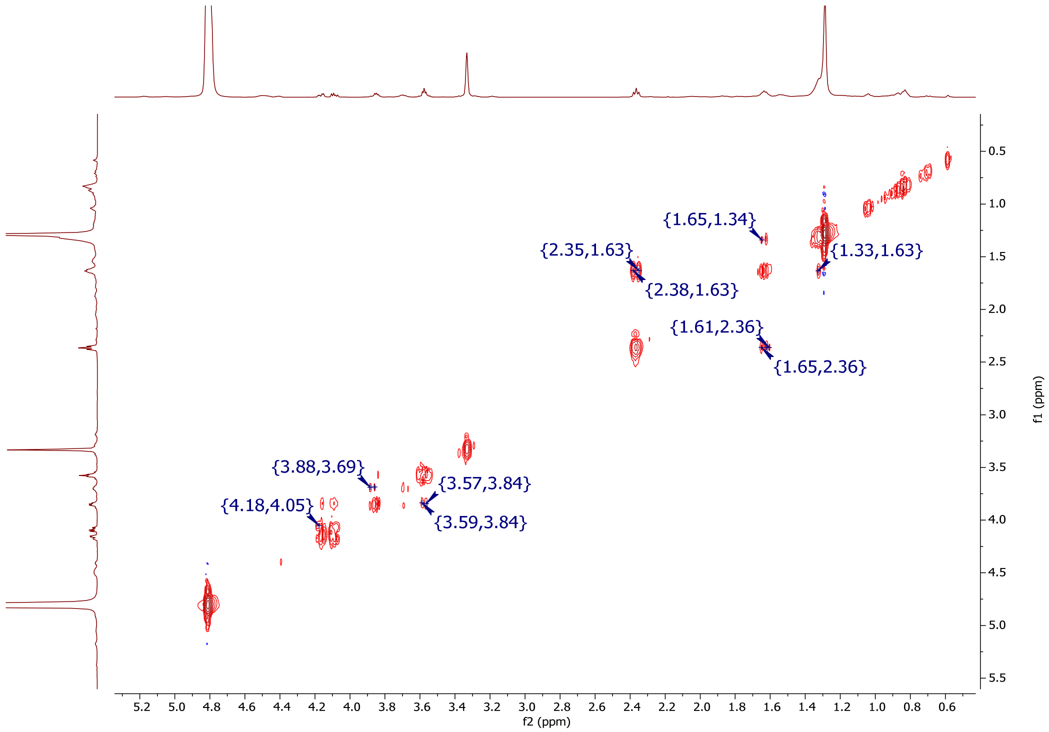

Figure S4: COSY spectrum of compound **1**

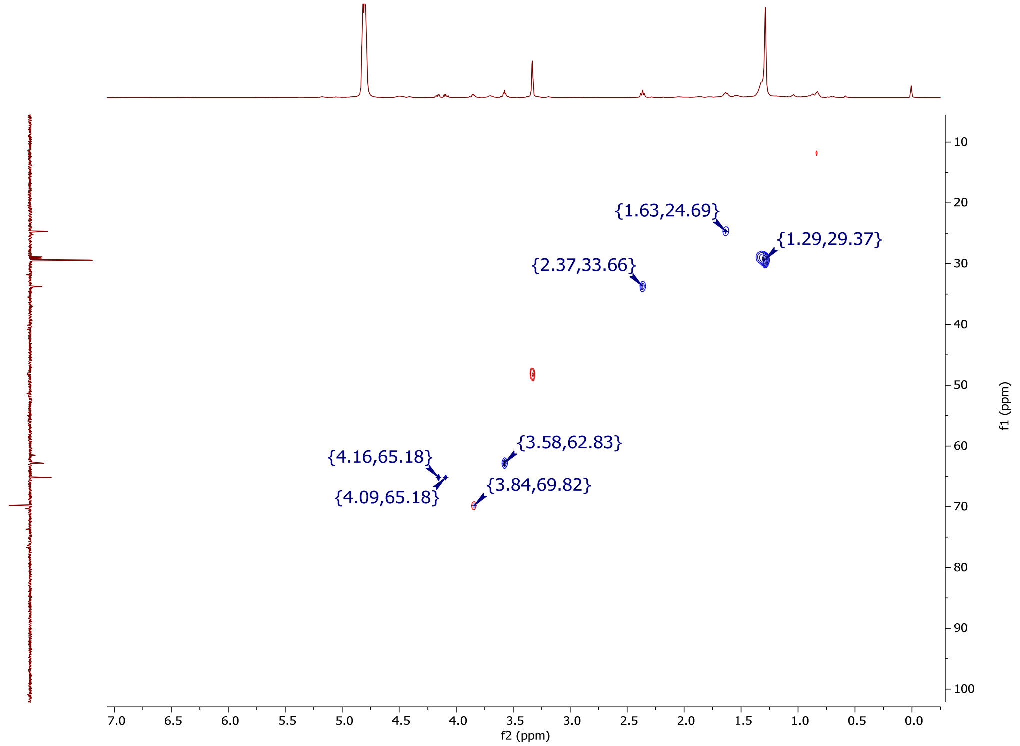

Figure S5: HSQC spectrum of compound **1**

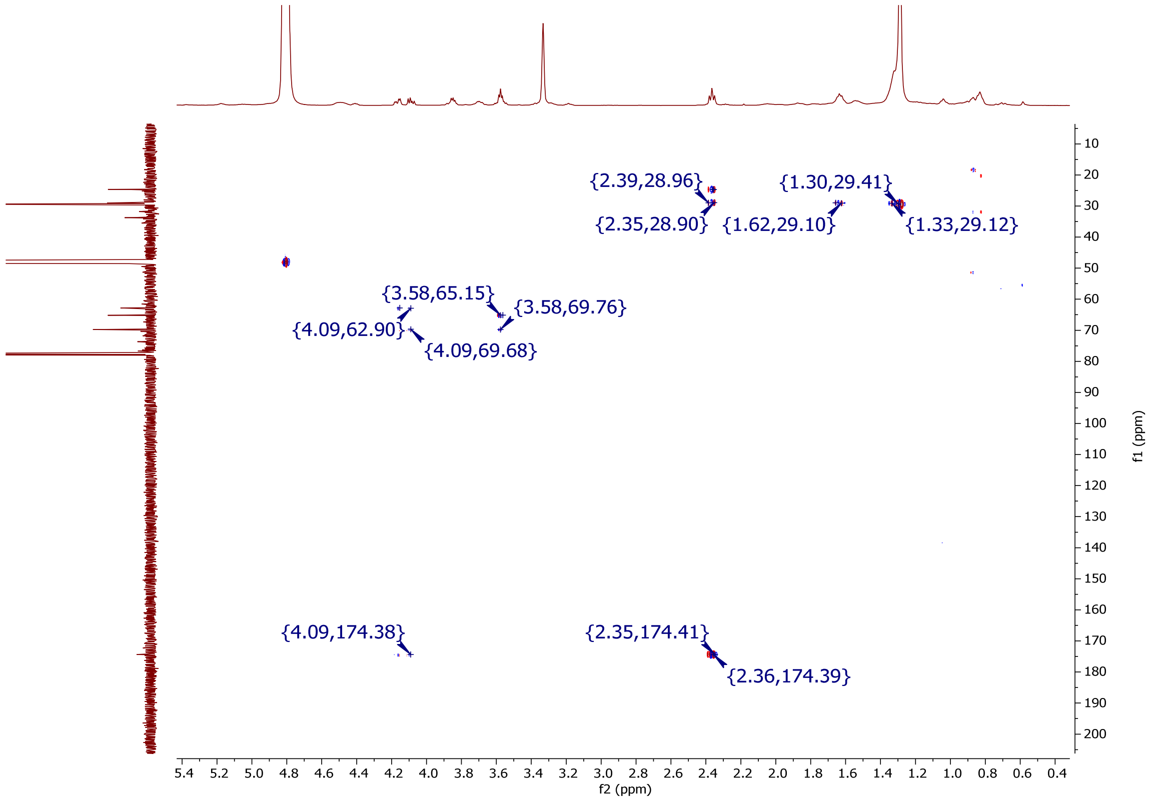

Figure S6: HMBC spectrum of compound **1**

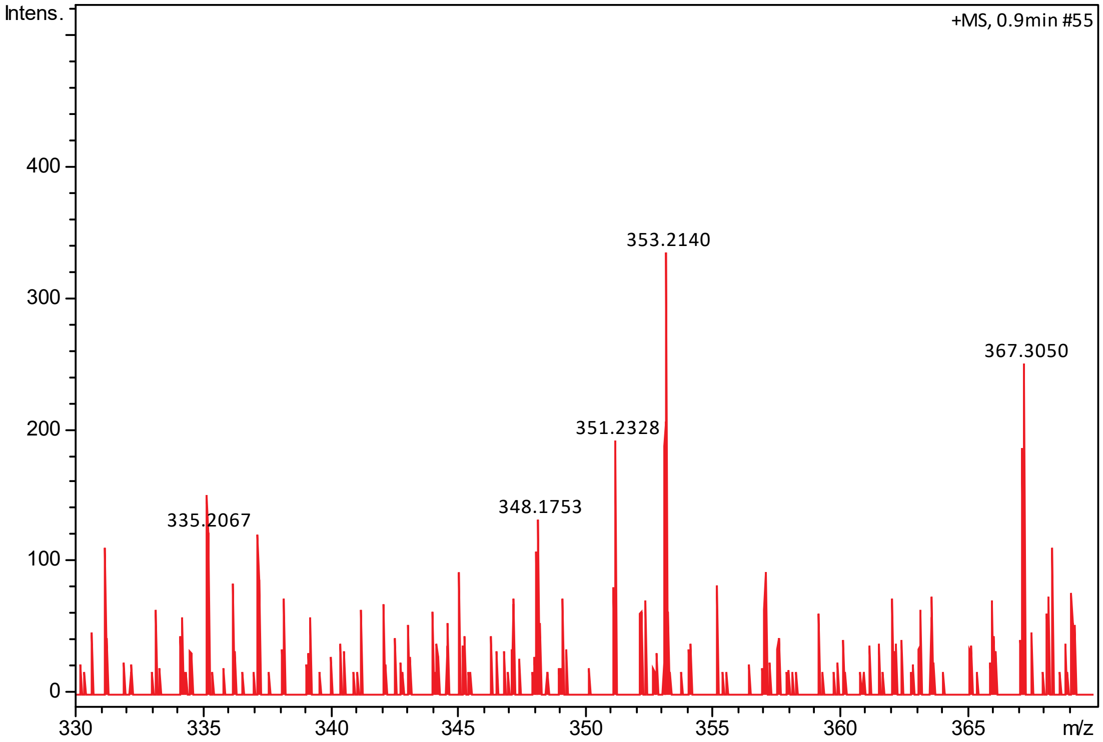

Figure S7: HR-ESI (+ve) spectrum of compound **2**

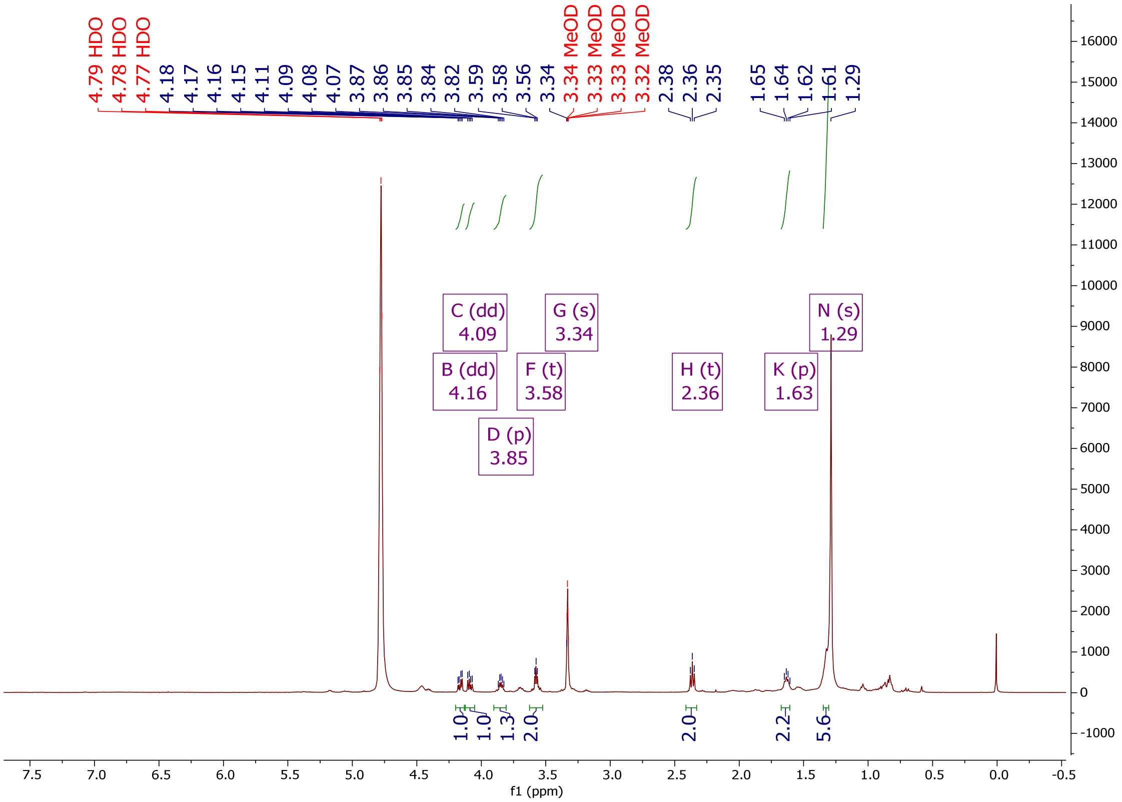

Figure S8: ^{1}H NMR (500 MHz, Methanol-d_4) spectrum of compound **2**

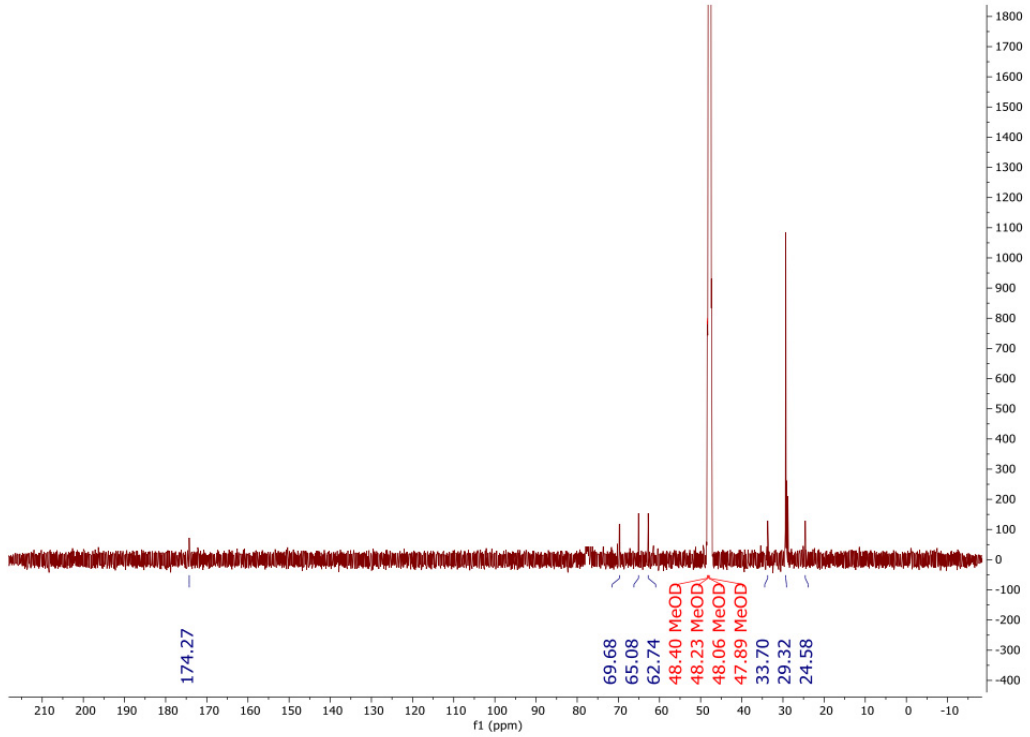

Figure S9: ^{13}C NMR (125 MHz, Methanol-d_4) spectrum of compound **2**

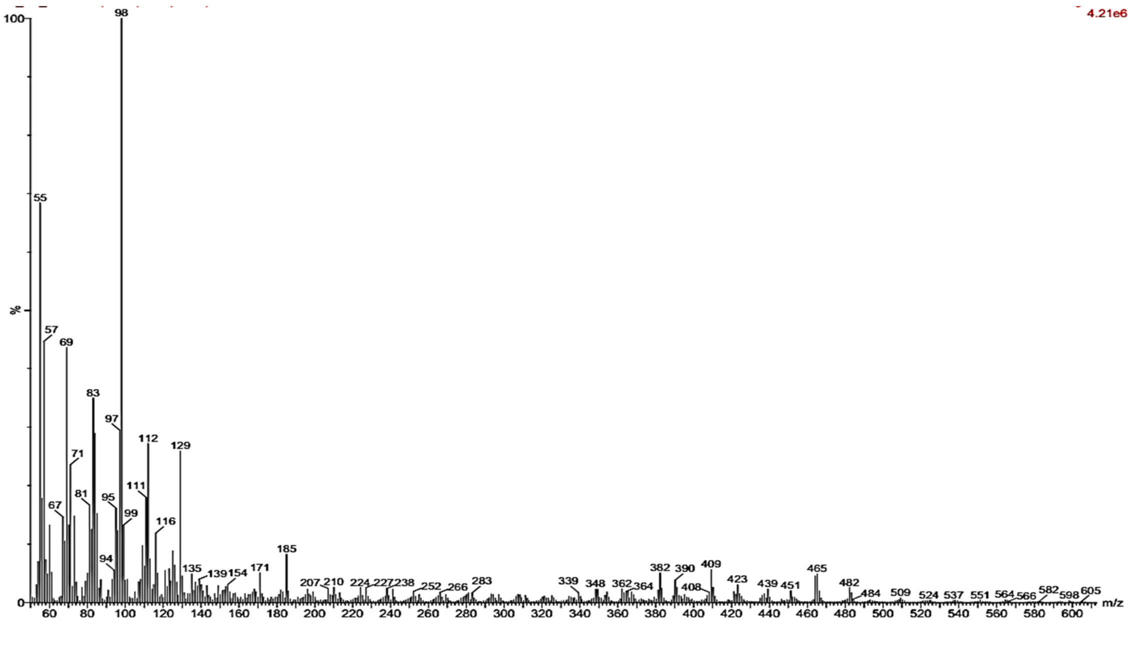

Figure S10: IE-MS spectrum of compound **3**

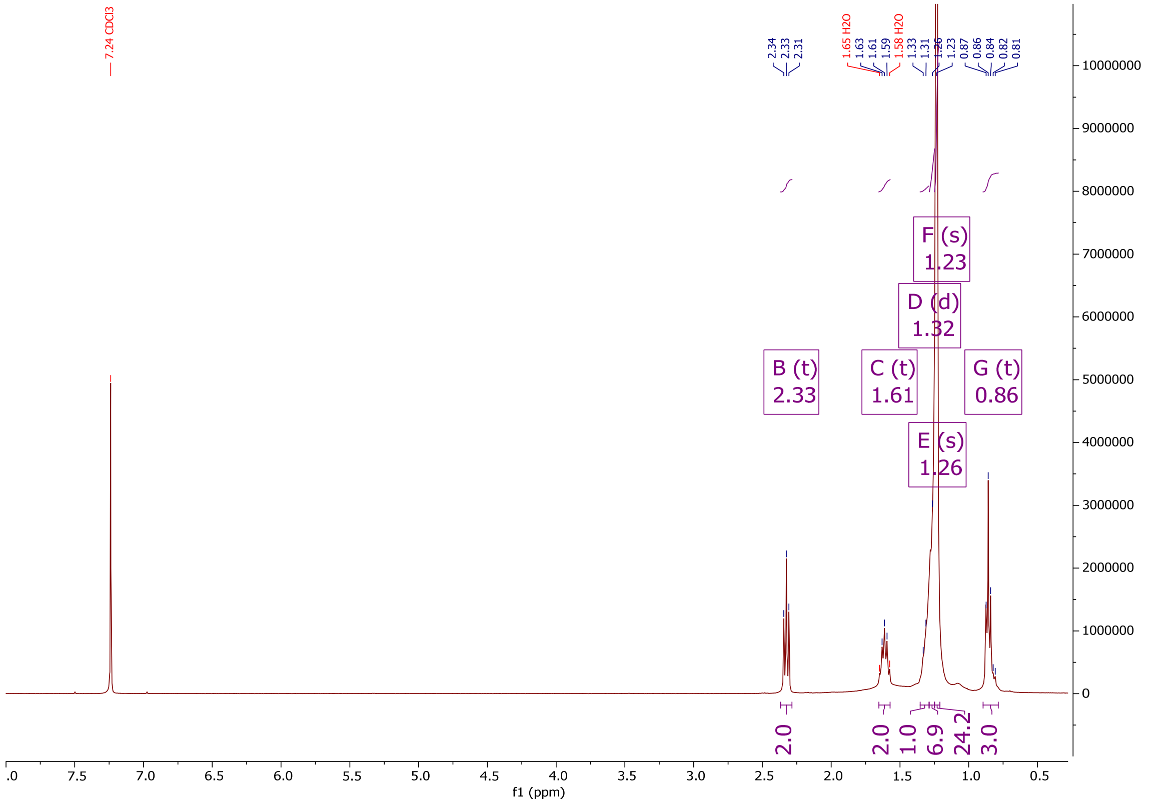

Figure S11: ^{1}H NMR (400 MHz, $CDCl_3$) spectrum of compound **3**

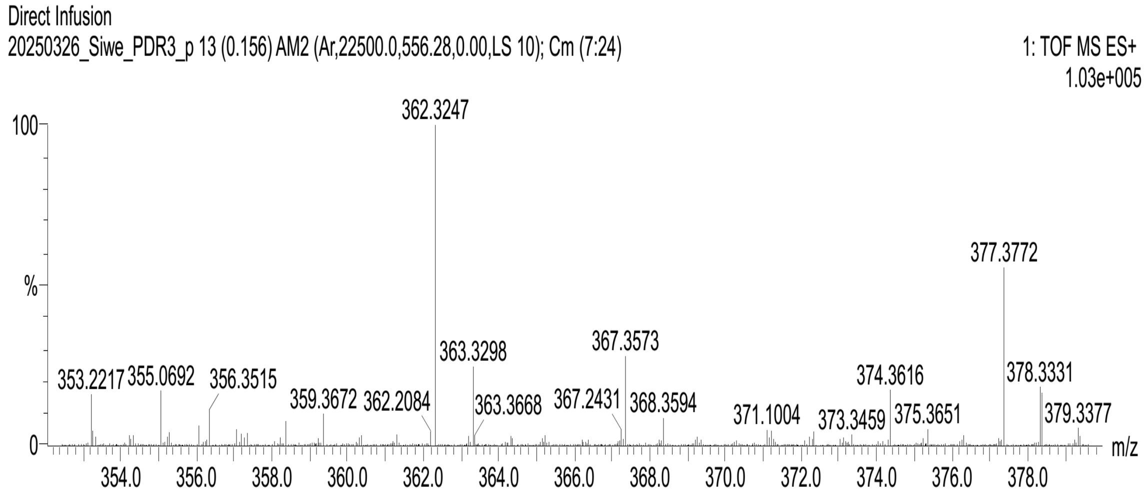

Figure S12: HR-ESI (+ve) spectrum of compound **4**

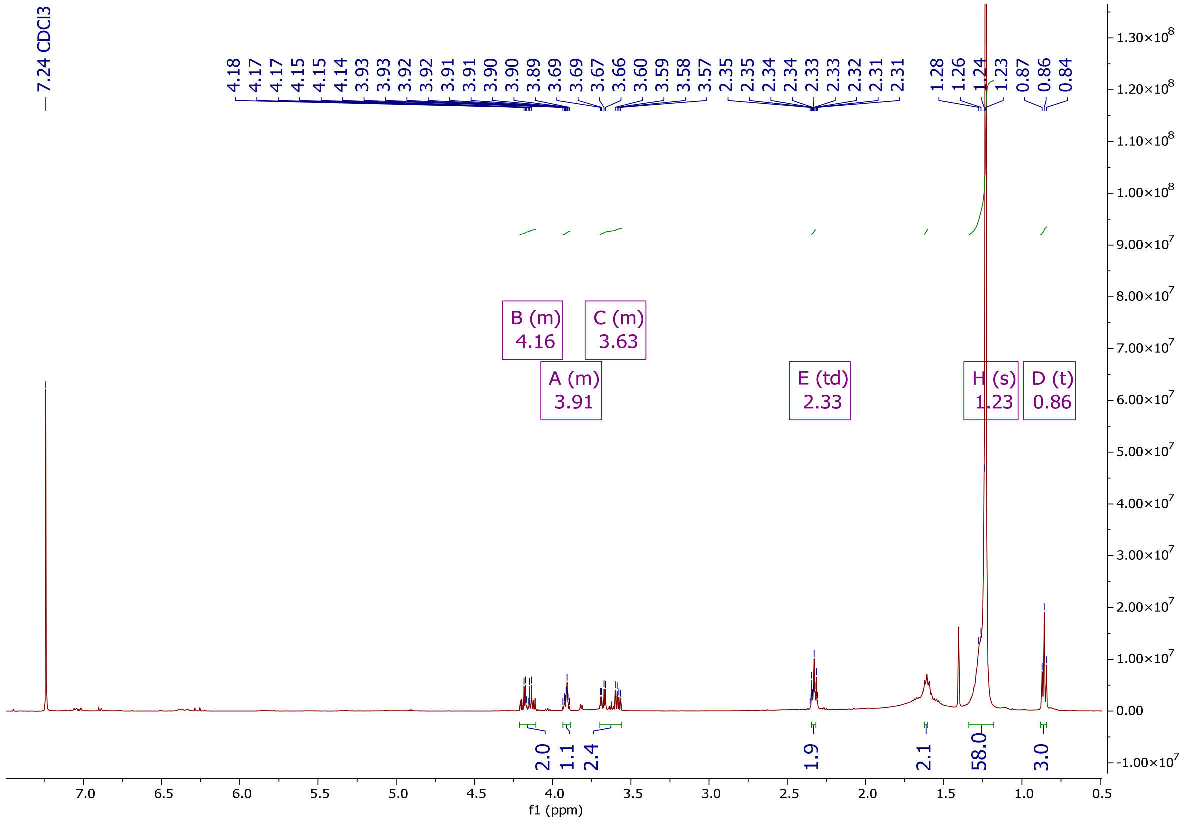

Figure S13: ^{1}H NMR (600 MHz, $CDCl_3$) spectrum of compound **4**

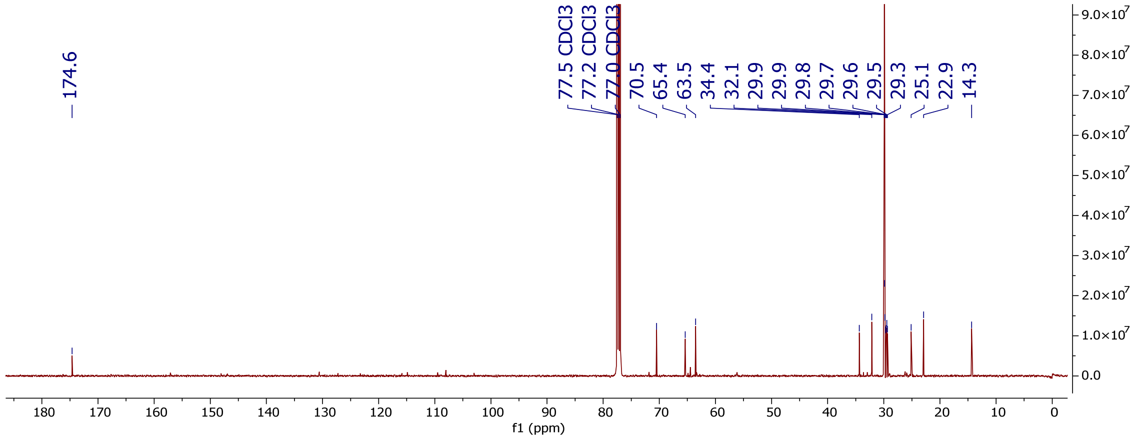

Figure S14: ^{13}C NMR (150 MHz, $CDCl_3$) spectrum of compound **4**

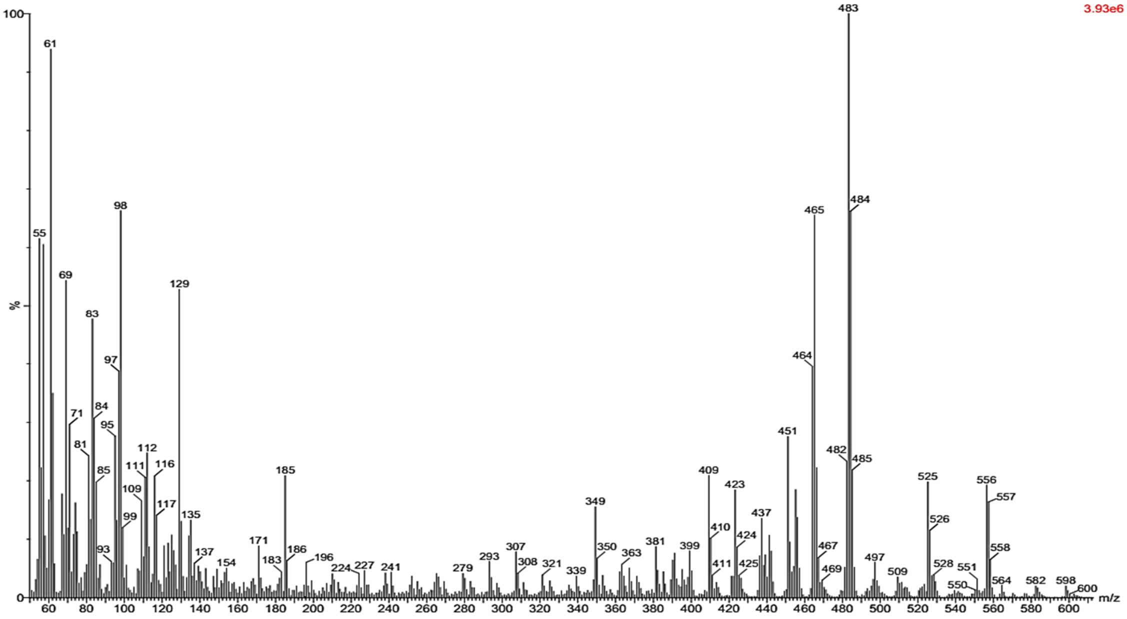

Figure S15: LR-EI-MS spectrum of compound **5**

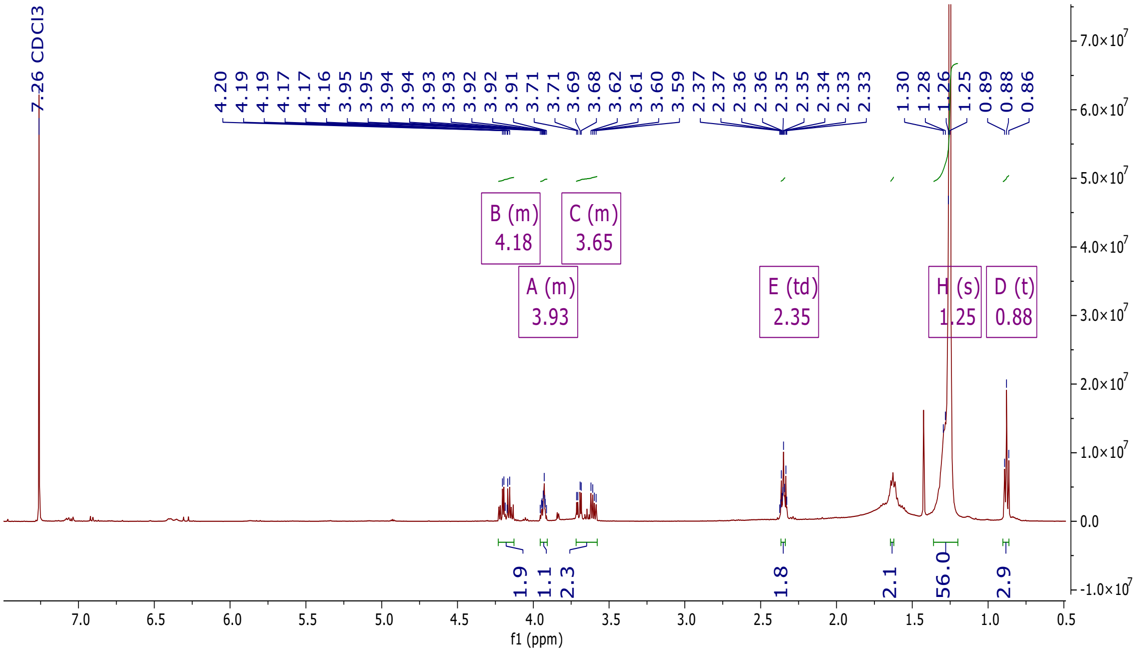

Figure S16: ^{1}H NMR (600 MHz, $CDCl_3$) spectrum of compound **5**

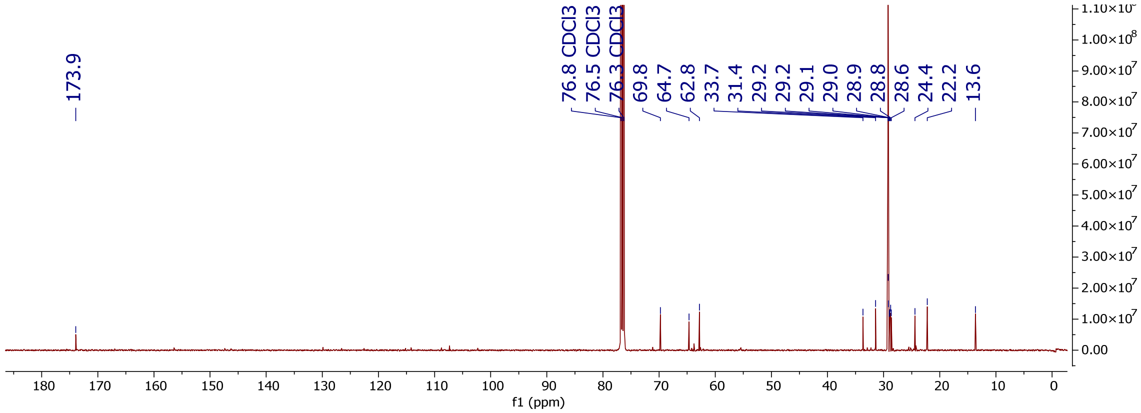

Figure S17: ^{13}C NMR (150 MHz, $CDCl_3$) spectrum of compound **5**

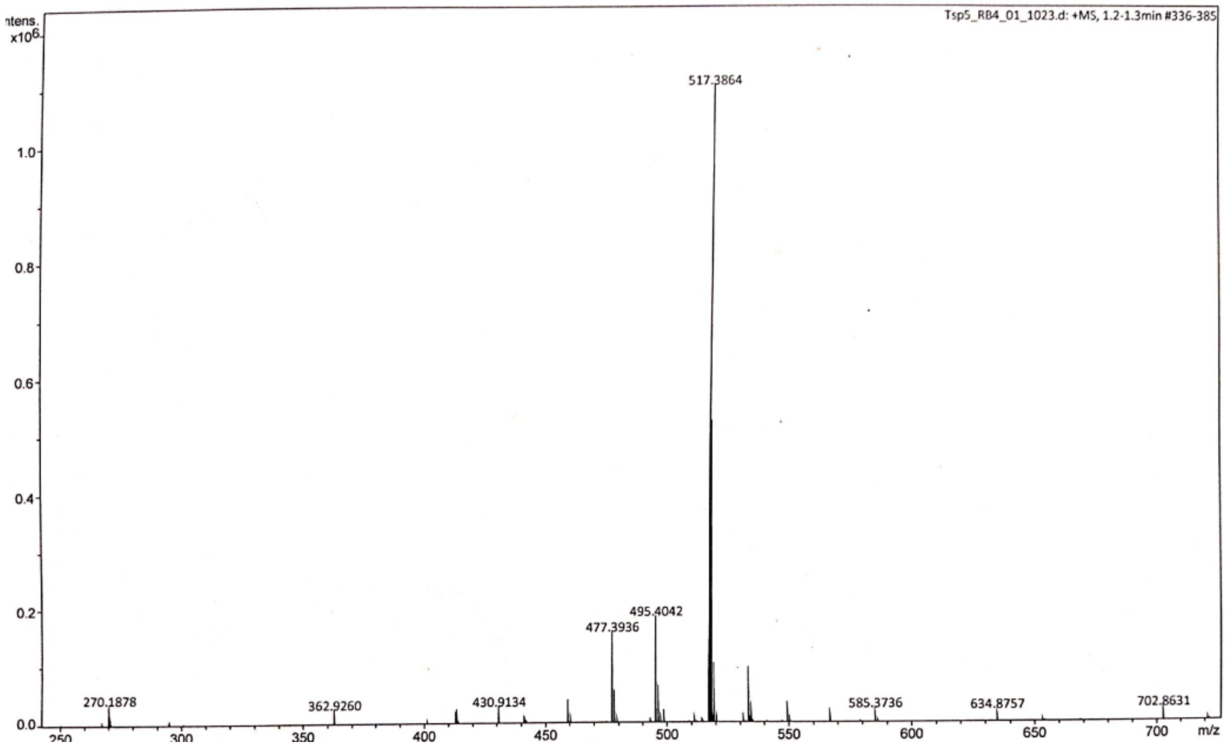

Figure S18: HR-ESI (+ve) spectrum of compound **6**

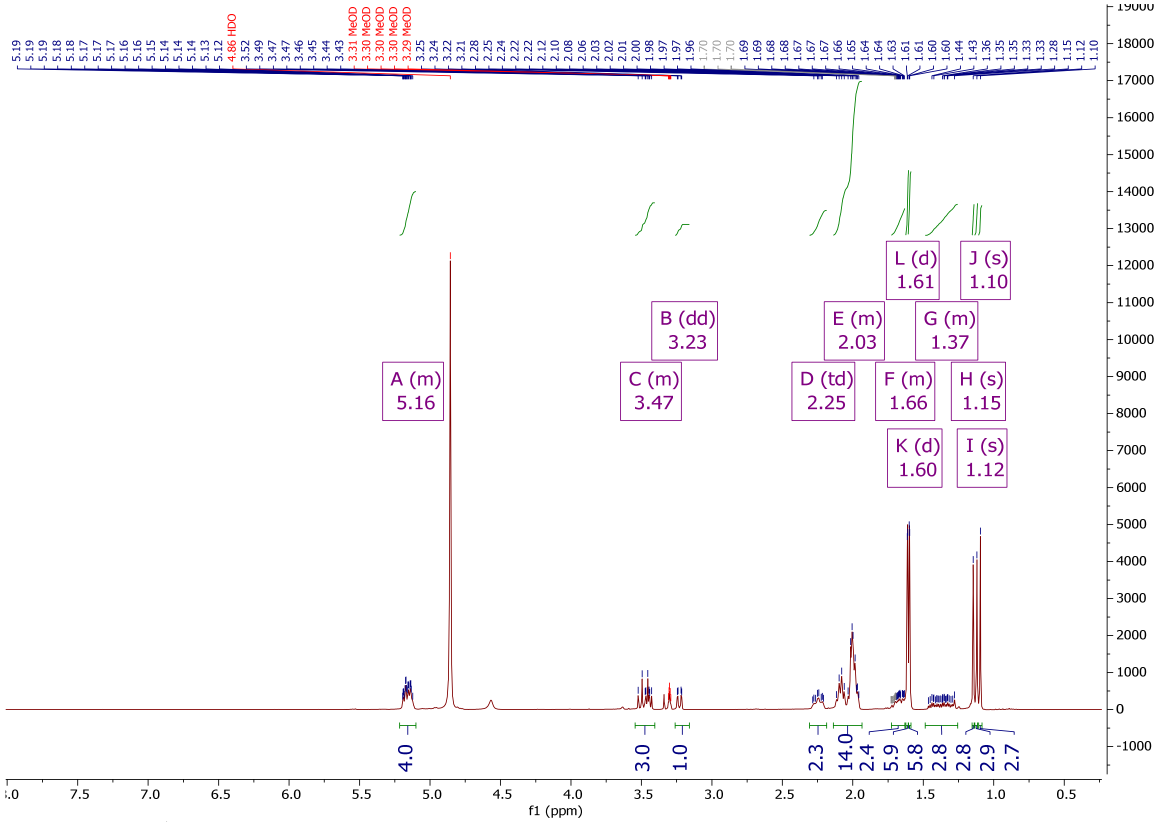

Figure S19: ^{1}H NMR (400 MHz, Methanol-d_4) spectrum of compound **6**

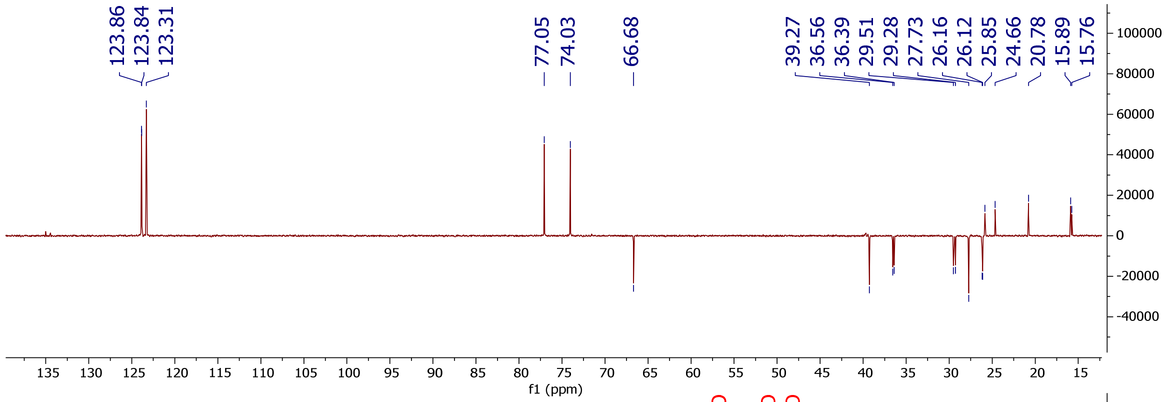

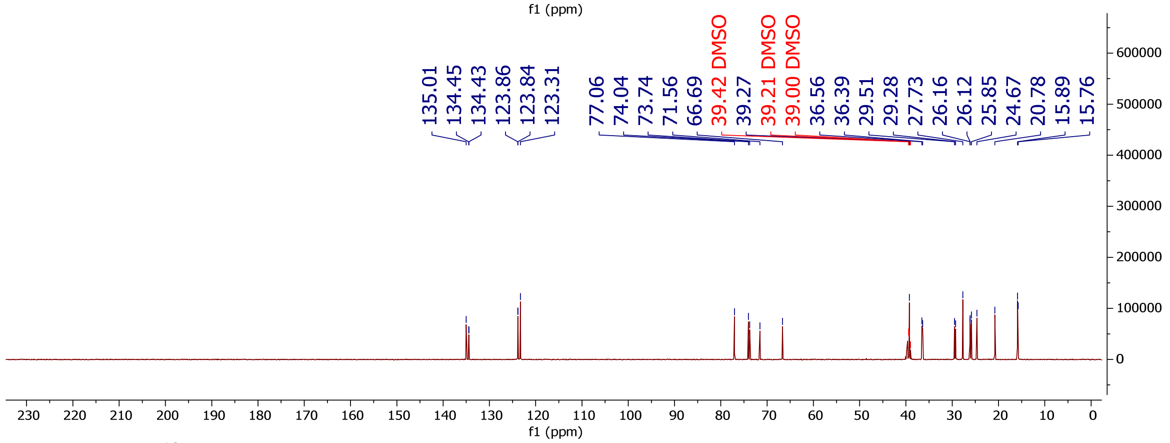

Figure S20: ^{13}C NMR and DEPT-135 (100 MHz, Methanol-d_4) spectra of compound **6**

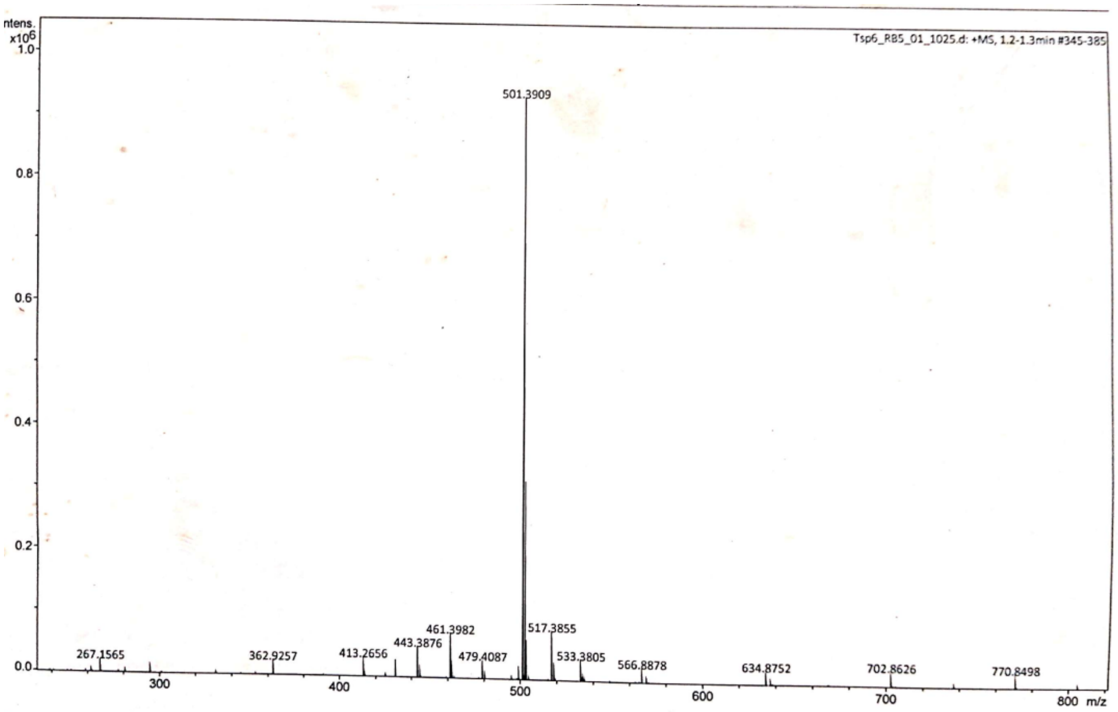

Figure S21: HR-ESI (+ve) spectrum of compound **7**

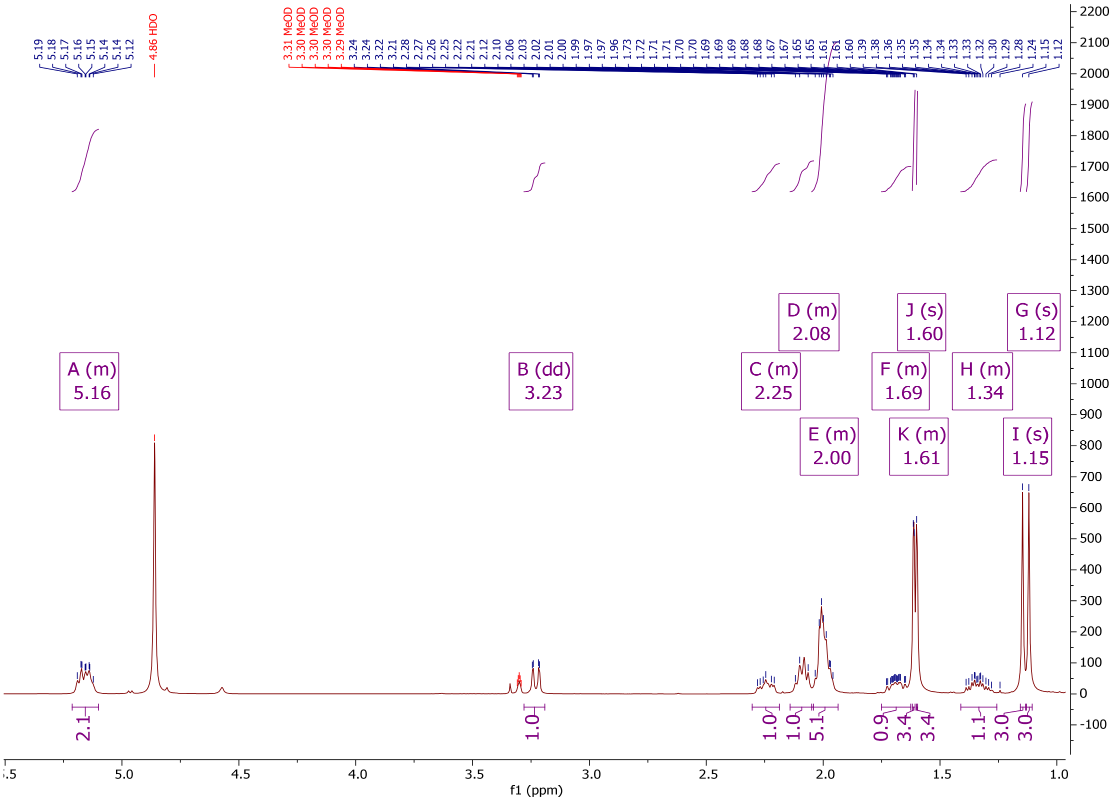

Figure S22: ^{1}H NMR (400 MHz, Methanol-d_4) spectrum of compound **7**

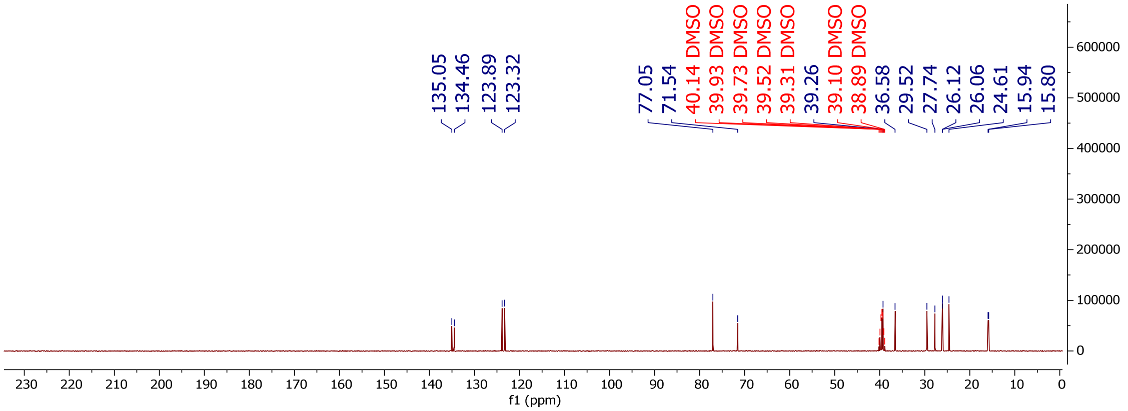

Figure S23: ^{13}C NMR (100 MHz, DMSO-d_6) spectrum of compound **7**

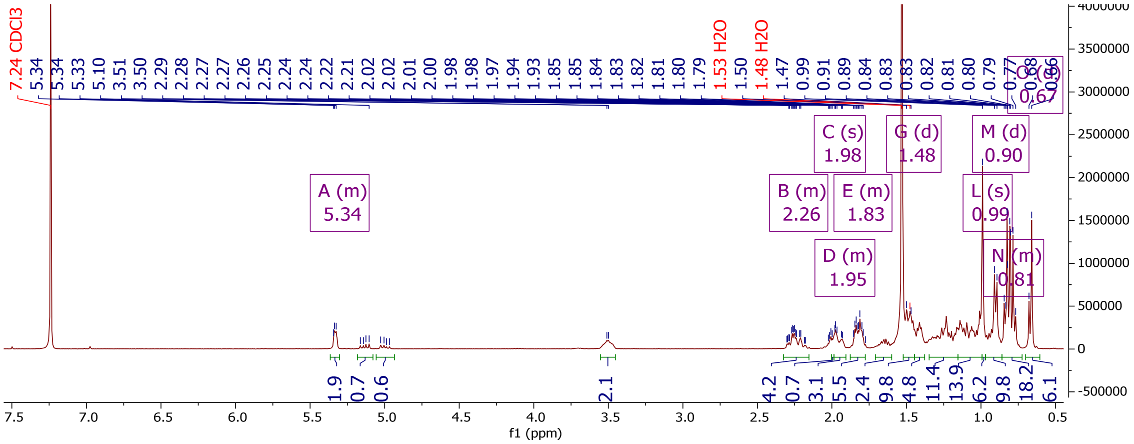

Figure S24: ^{1}H NMR (400 MHz, CDCl$_3$) spectrum of the mixture of compounds **8** and **9**

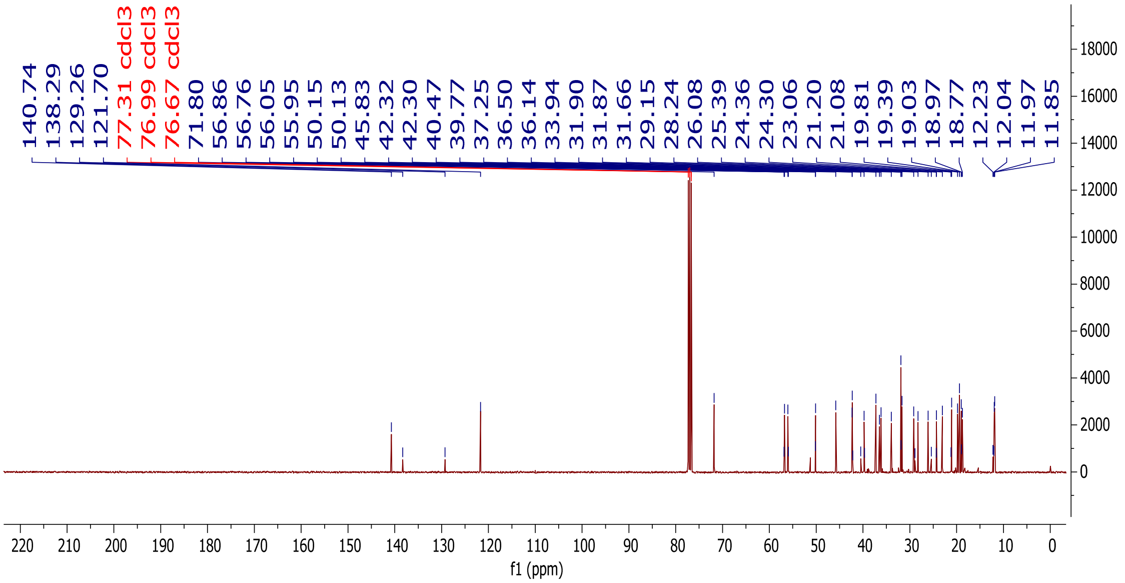

Figure S25: ^{13}C NMR (100 MHz, CDCl$_3$) spectrum of the mixture of compounds **8** and **9**

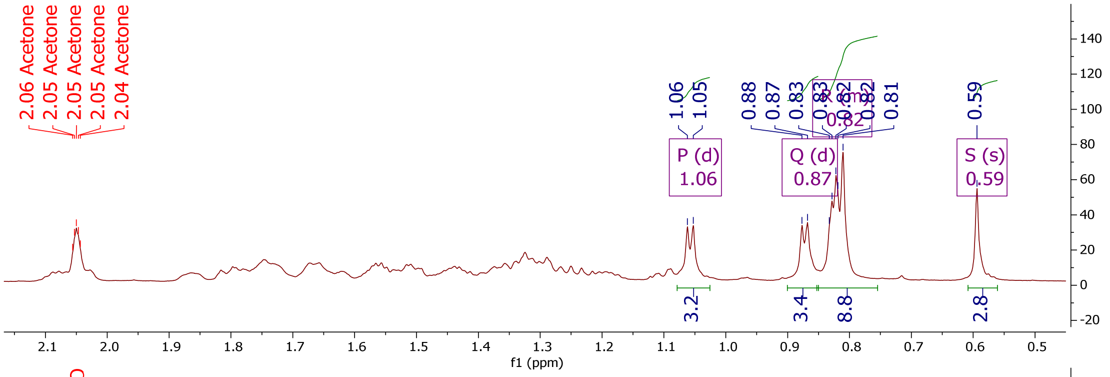

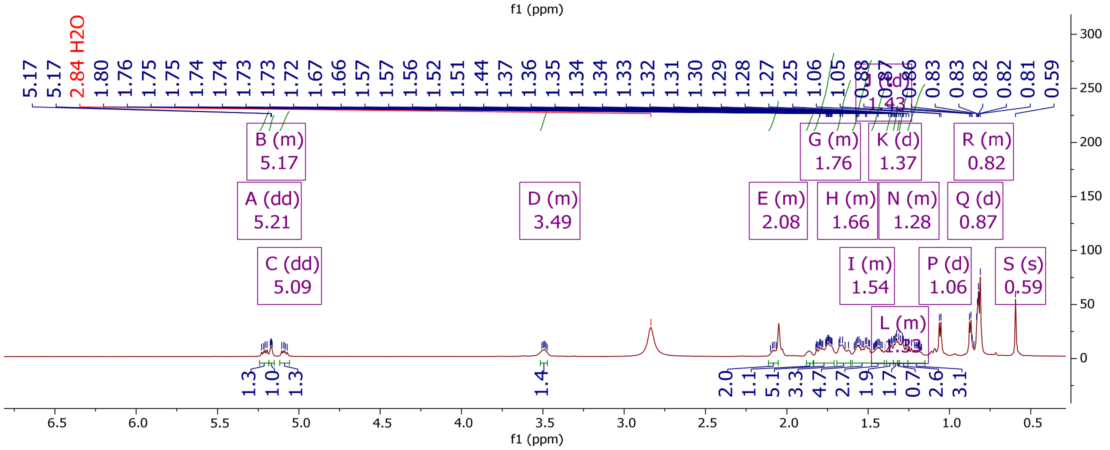

Figure S26: ^{1}H NMR (700 MHz, Acetone-d_6) spectrum compound **10**

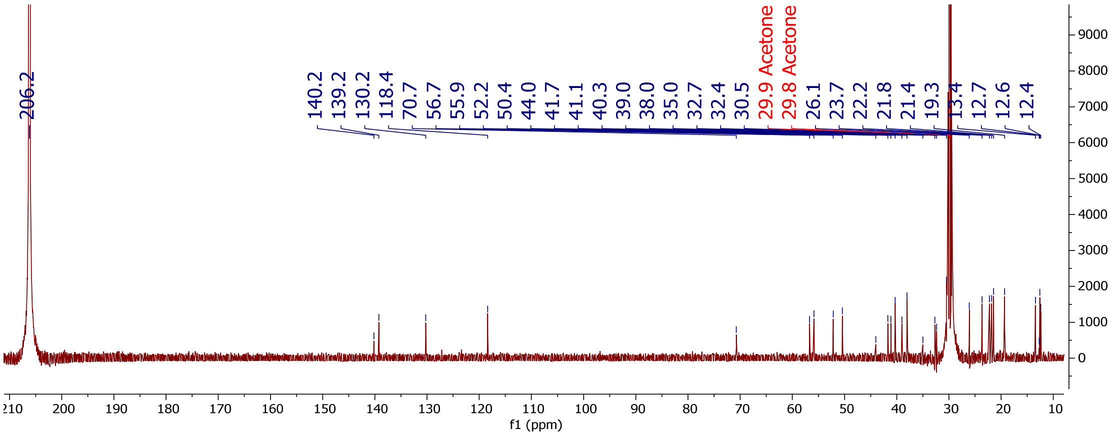

Figure S27: ^{13}C NMR (175 MHz, Acetone-d_6) spectrum of compound **10**

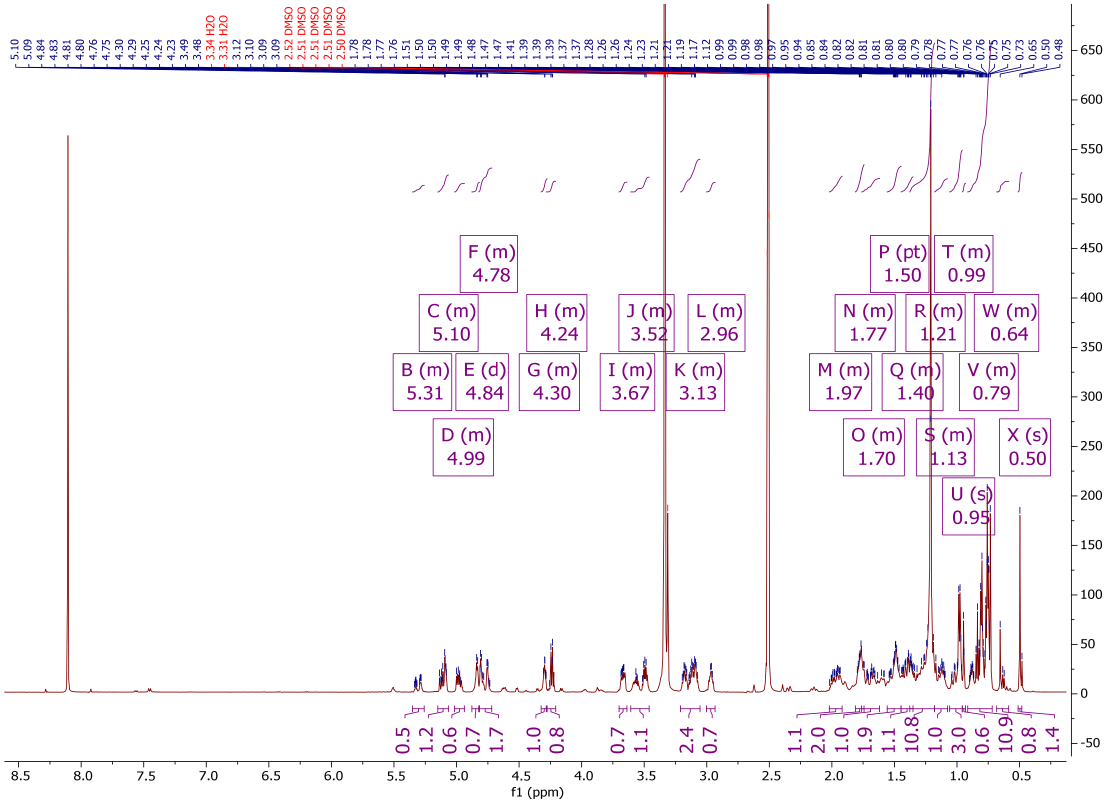

Figure 28: ^{1}H NMR (600 MHz, DMSO-d_6) spectra of compounds **11**, **12**, and **13**

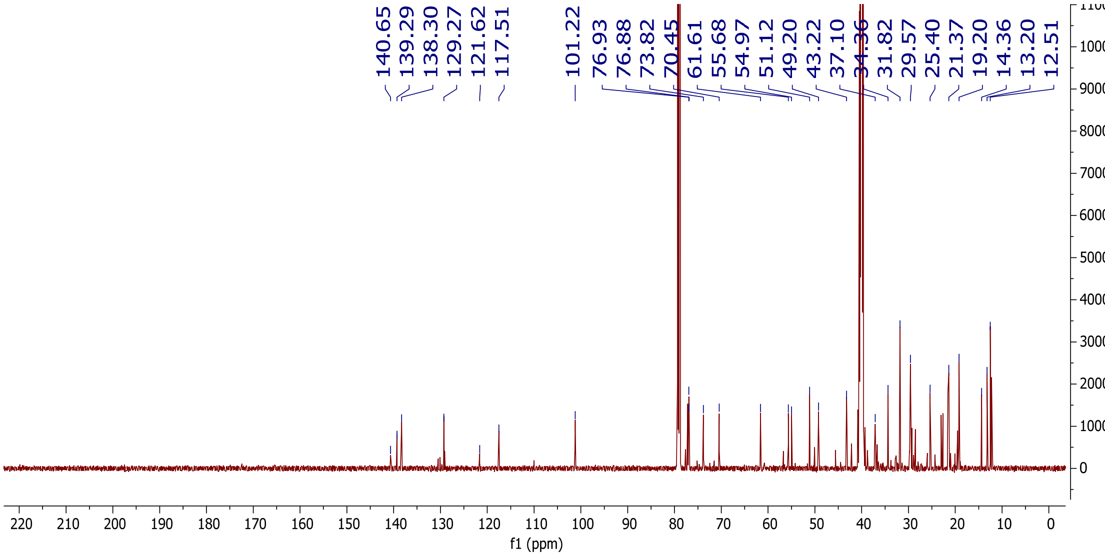

Figure 29: ^{13}C NMR (150 MHz, DMSO-d_6) spectra of compounds **11**, **12**, and **13**

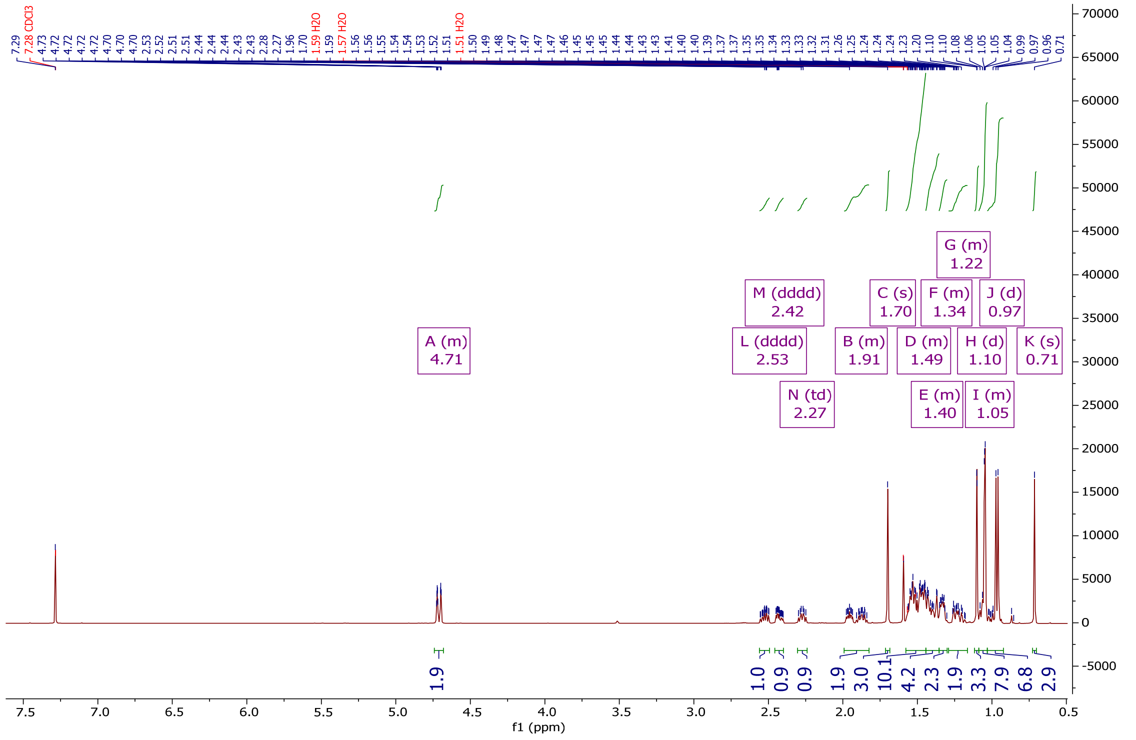

Figure **S30**: ^{1}H NMR (600 MHz, $CDCl_3$) spectrum of compound **14**

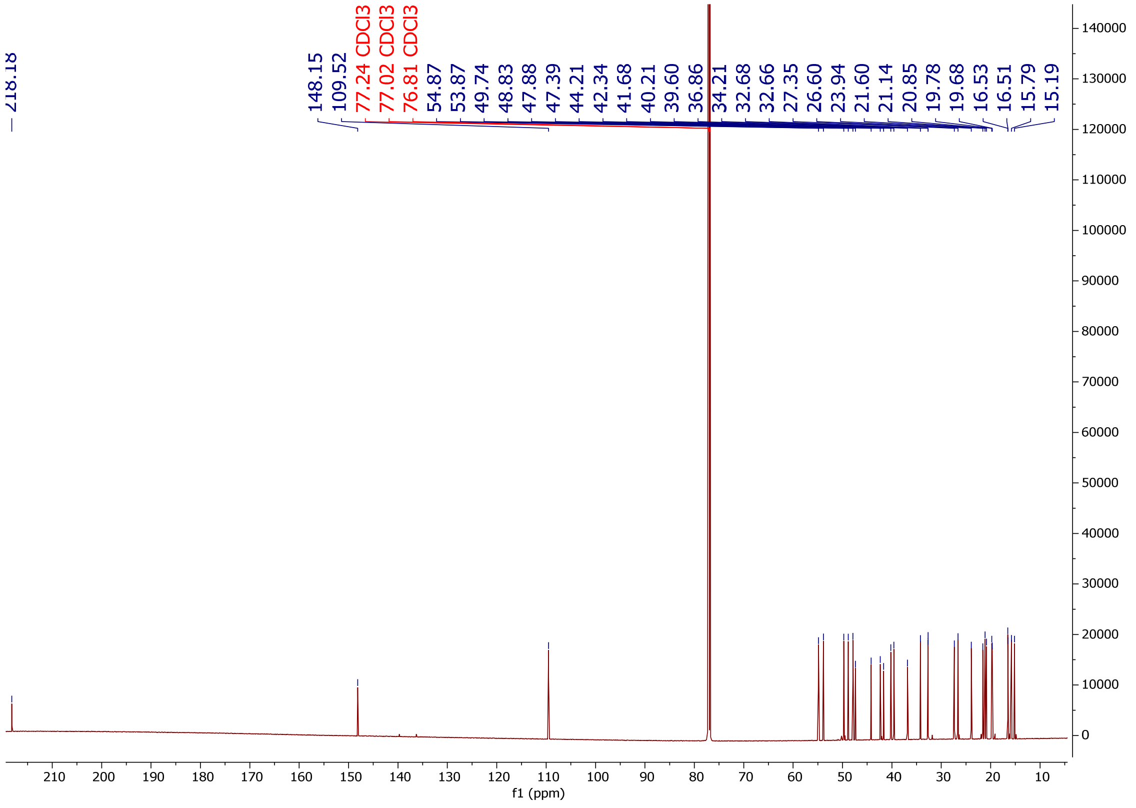

Figure **S31**: ^{13}C NMR (150 MHz, $CDCl_3$) spectrum of compound **14**

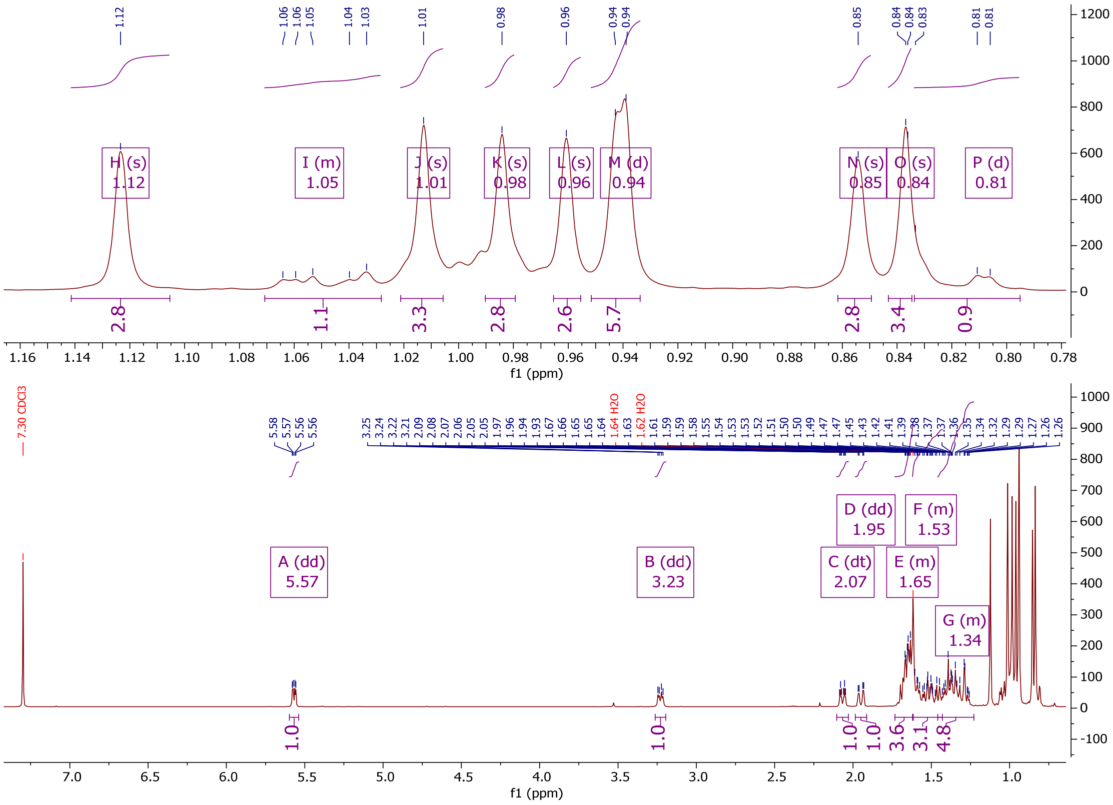

Figure S32: ^{1}H NMR (500 MHz, $CDCl_3$) spectrum of compound **15**

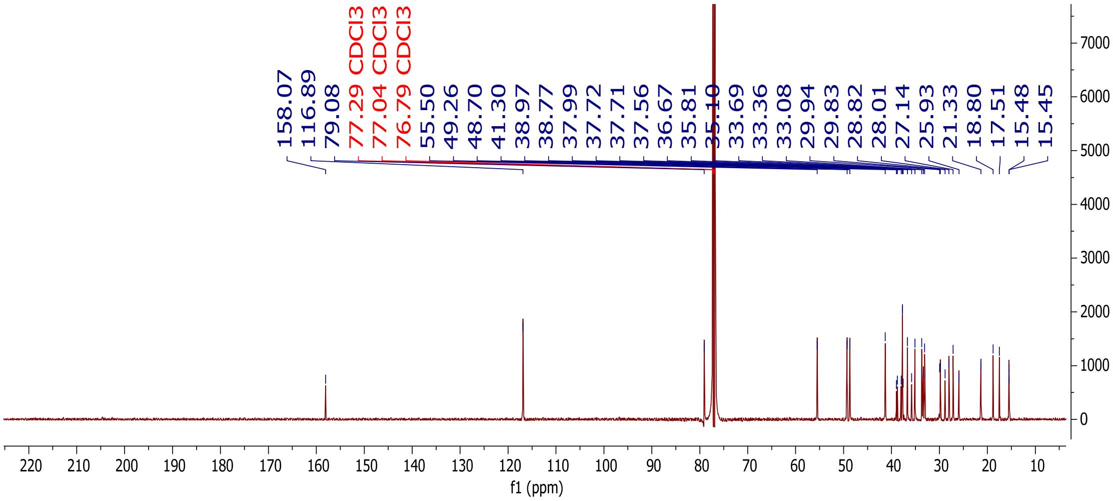

Figure S33: ^{13}C NMR (126 MHz, $CDCl_3$) spectrum of compound **15**

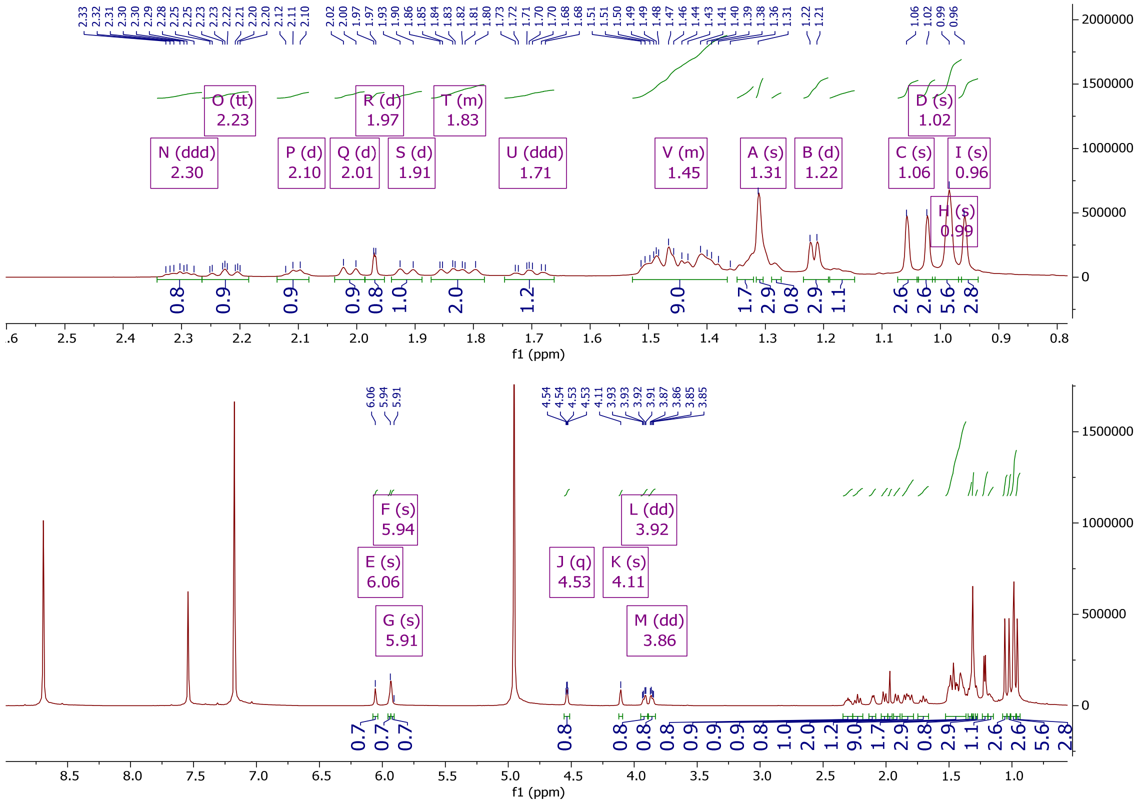

Figure S34: ^{1}H NMR (600 MHz, Pyridine-d_5) spectrum of compound **16**

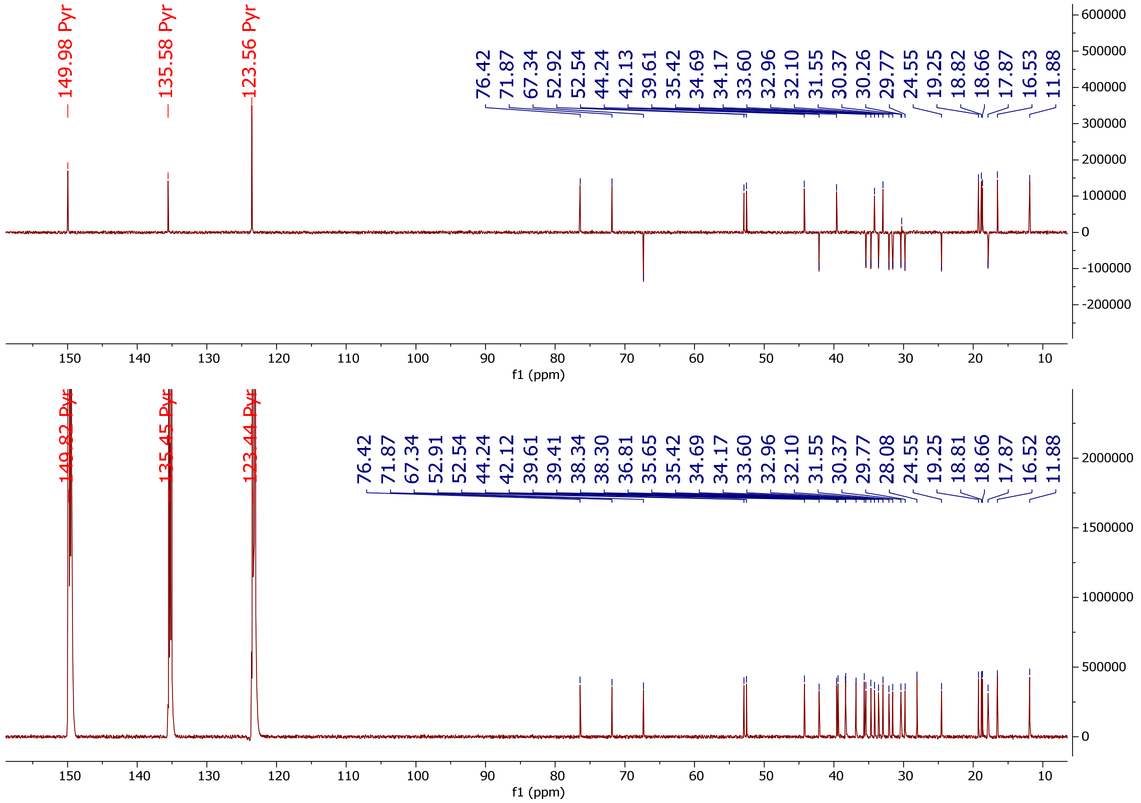

Figure S35: ^{13}C NMR and DEPT-135 (150 MHz, Pyridine-d_5) spectra of compound **16**

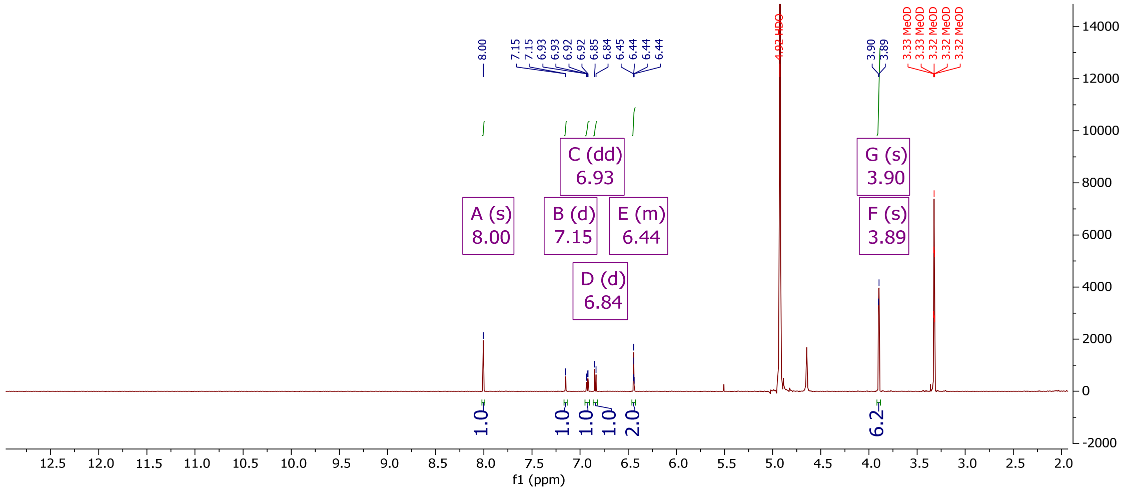

Figure S36: ^{1}H NMR (600 MHz, Methanol-d_4) spectrum of compound **17**

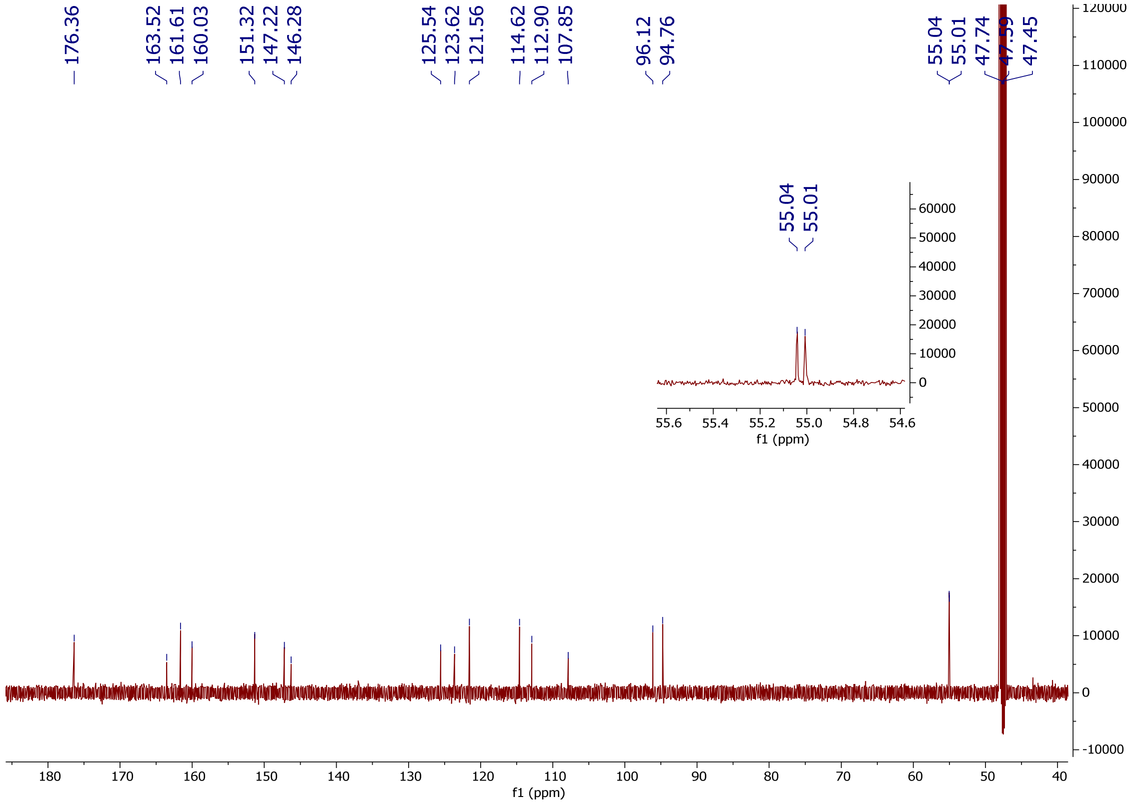

Figure S37: ^{13}C NMR (150 MHz, Methanol-d_4) spectrum of compound **17**

Table S1: Docking energies with 4GQR for *α*-amylase and types of interaction involved

Ligands	Docking score (kcal/mol)	Amino acid, interaction type, distance and energy involved				
		Ligand	Receptor	Interaction	Distance (Å)	E (kcal/mol)
**1**	-9.08	O 68	OE2 GLU 233 (A)	H-donor	2.76	-3.2
		O 1	NE2 HIS 299 (A)	H-acceptor	3.01	-1.9
		O 68	NH2 ARG 195 (A)	H-acceptor	3.09	-1.5
**2**	-8.34	O 1	OD1 ASP 300 (A)	H-donor	2.88	-2.9
		O 44	OE2 GLU 233 (A)	H-donor	2.71	-1.2
		O 50	O THR 163 (A)	H-donor	3.17	-1.3
		O 1	N ALA 307 (A)	H-acceptor	2.87	-2.0
**3**	-8.52	O 75	OE2 GLU 233 (A)	H-donor	2.70	-2.9
**4**	-8.06	O 60	OD1 ASP 300 (A)	H-donor	2.89	-3.3
		O 62	OE2 GLU 233 (A)	H-donor	2.86	-2.8
		O 64	NE2 HIS 299 (A)	H-acceptor	2.93	-3.9
**5**	-9.34	O 63	CE LYS 200 (A)	H-acceptor	3.33	-0.8
**7**	-9.50	O 81	OE2 GLU 233 (A)	H-donor	3.00	-2.6
		O 87	OD1 ASP 197 (A)	H-donor	3.03	-2.2
		O 38	ND2 ASN 53 (A)	H-acceptor	3.10	-1.8
		O 87	NE2 HIS 299 (A)	H-acceptor	3.22	-1.7
**6**	-9.64	O 88	OD1 ASP 300 (A)	H-donor	2.82	-2.0
		O 80	N SER 108 (A)	H-acceptor	3.03	-2.4
		O 80	OG SER 108 (A)	H-acceptor	3.04	-0.9
**16**	-7.86	O 73	O THR 163 (A)	H-donor	2.88	-1.4
**15**	-7.22	O 70	OD1 ASP 197 (A)	H-donor	2.69	-1.8
		C 35	6-ring TRP 59 (A)	H-pi	4.38	-0.5
**8**	-7.73	O 3	OE2 GLU 233 (A)	H-donor	2.88	-2.2
		C 25	5-ring TRP 59 (A)	H-pi	3.72	-0.6
**9**	-7.98	O 3	OD1 ASP 197 (A)	H-donor	2.83	-3.0
**10**	-.786	O 3	OD1 ASP 197 (A)	H-donor	2.81	-3.0
		O 3	NH2 ARG 195 (A)	H-acceptor	3.32	-0.5
**11**	-9.63	O 93	OE1 GLU 233 (A)	H-donor	3.21	-1.2
		O 95	OD1 ASP 300 (A)	H-donor	2.77	-3.8
		O 99	NE2 HIS 299 (A)	H-acceptor	3.05	-2.4
**12**	-9.15	O 93	OD2 ASP 197 (A)	H-donor	2.90	-3.2
		O 95	OE1 GLU 233 (A)	H-donor	3.01	-3.3
		O 91	NE2 HIS 299 (A)	H-acceptor	3.11	-1.6
**13**	-9.35	C 86	OE2 GLU 233 (A)	H-donor	3.17	-0.6
		O 93	OD1 ASP 300 (A)	H-donor	2.76	-3.8
		O 95	OE2 GLU 233 (A)	H-donor	2.79	-2.9
		O 97	OD1 ASP 197 (A)	H-donor	2.91	-1.5
		O 97	NE2 HIS 299 (A)	H-acceptor	2.96	-2.9
**14**	-7.45	C 30	6-ring TRP 59 (A)	H-pi	3.64	-0.6
**17**	-6.41	O 36	OG1 THR 163 (A)	H-donor	2.78	-1.2
		6-ring	CD1 LEU 165 (A)	pi-H	4.30	-0.6
**Acarbose**	-9.70	O 82	OD1 ASP 300 (A)	H-donor	2.81	-1.2
		O 84	OE2 GLU 233 (A)	H-donor	2.89	-4.3
		O 86	OD1 ASP 197 (A)	H-donor	2.96	-0.7
		O 59	N ALA 106 (A)	H-acceptor	3.20	-1.2
		O 86	NE2 HIS 299 (A)	H-acceptor	2.95	-3.1
		N 12	6-ring TYR 62 (A)	H-pi	4.50	-0.5

NI = No visible interaction, their docking score may be due to their hydrophobic interactions

Table S2: 2D interactions with 4GQR for *α*-amylase

**Ligands**	**2D**
**1**	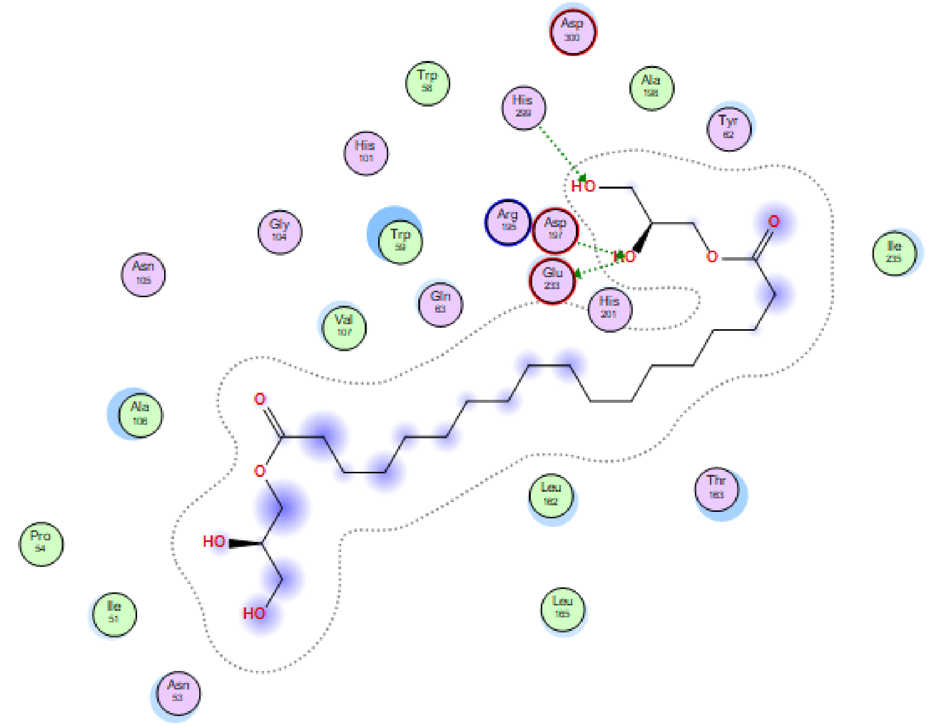
**2**	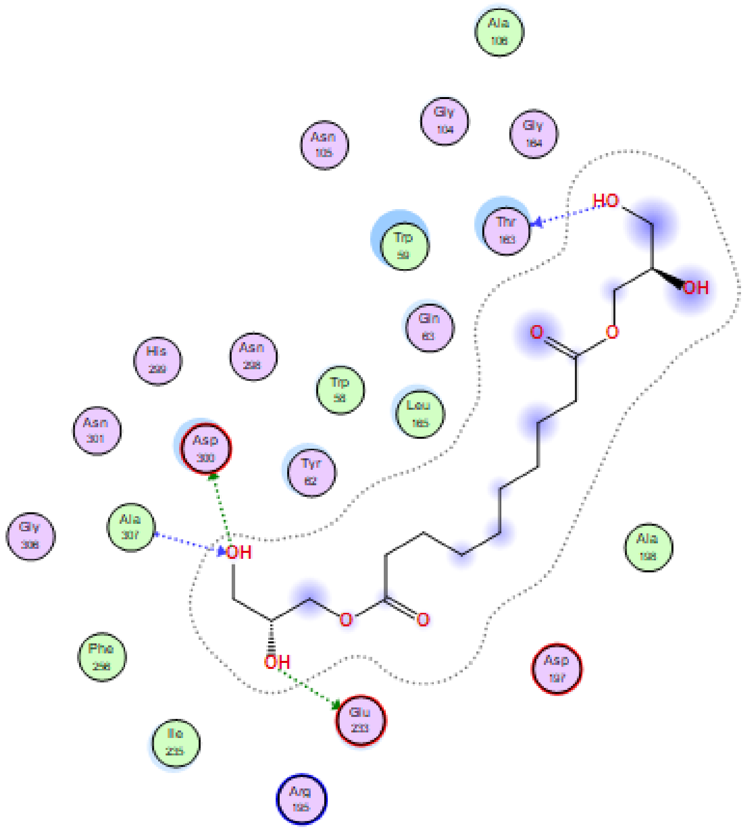

**3**	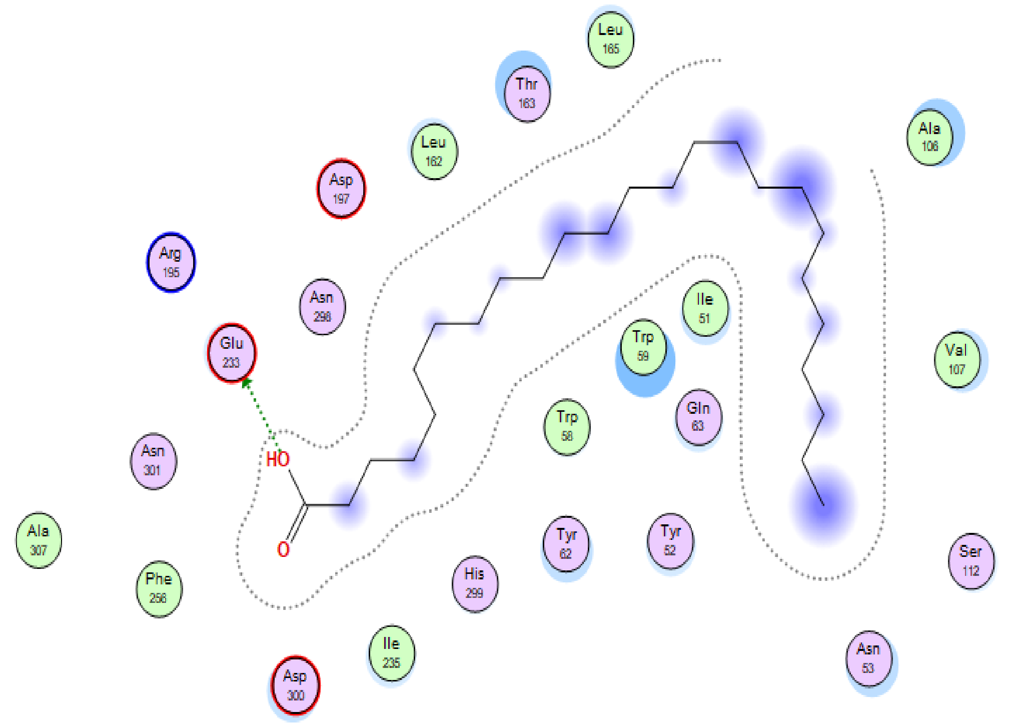 Leu 165<br>Thr 163<br>Leu 162<br>Asp 197<br>Ala 106<br>Arg 195<br>Asn 298<br>Ile 51<br>Trp 59<br>Glu 233<br>Val 107<br>Gln 63<br>Trp 58<br>Asn 301<br>HO<br>O<br>Ala 307<br>Tyr 62<br>Tyr 52<br>Ser 112<br>Phe 256<br>His 299<br>Ile 235<br>Asn 53<br>Asp 300
**4**	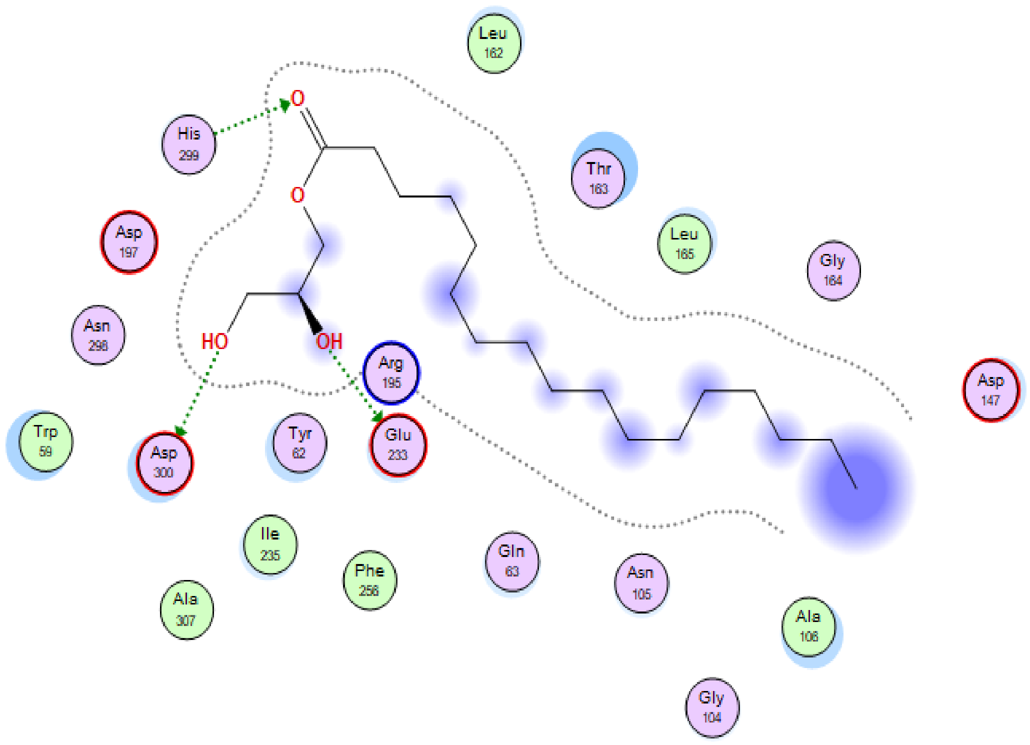 Leu 162<br>O<br>His 299<br>Thr 163<br>O<br>Asp 197<br>Leu 165<br>Gly 164<br>Asn 298<br>HO<br>OH<br>Arg 195<br>Asp 147<br>Trp 59<br>Asp 300<br>Tyr 62<br>Glu 233<br>Ile 235<br>Phe 256<br>Gln 63<br>Asn 105<br>Ala 106<br>Ala 307<br>Gly 104

5

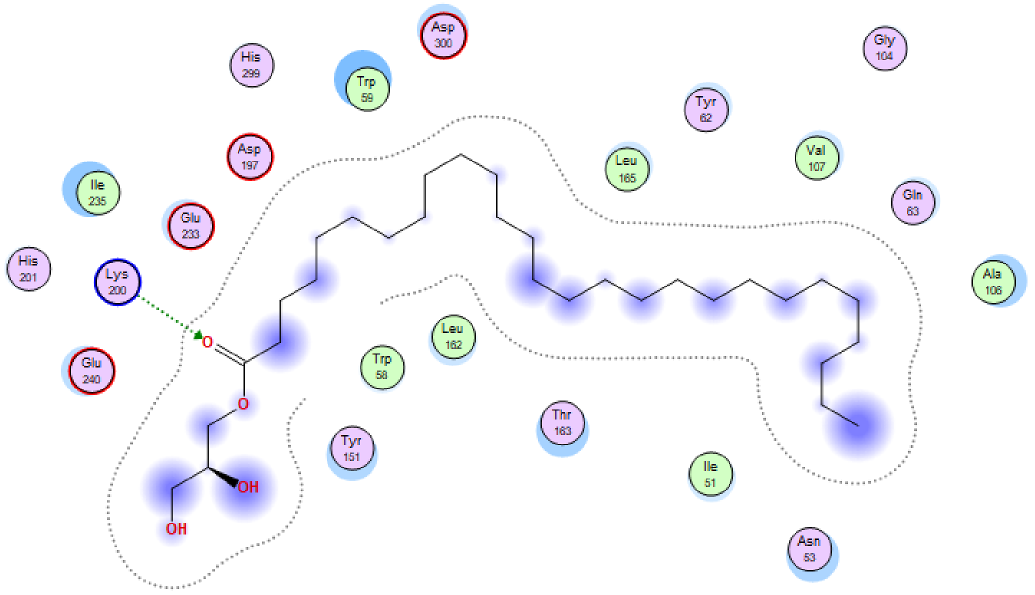
Asp 300
His 299
Trp 59
Gly 104
Tyr 62
Asp 197
Leu 165
Val 107
Ile 235
Glu 233
Gln 63
His 201
Lys 200
Ala 108
O
Leu 162
Glu 240
Trp 58
O
Thr 163
Tyr 151
OH
Ile 51
OH
Asn 53

6

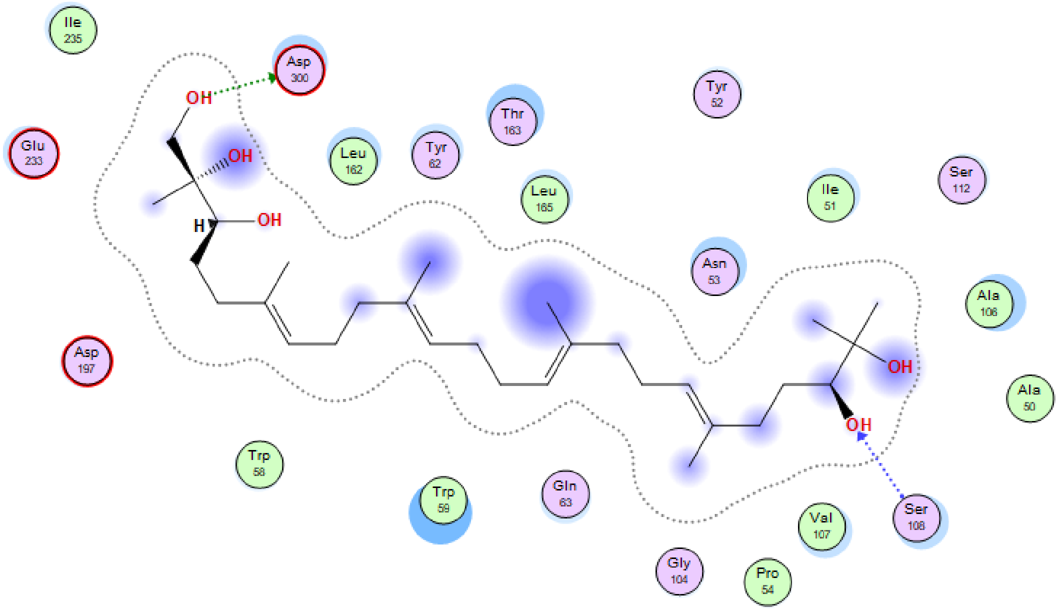
Ile 235
Asp 300
OH
Thr 163
Tyr 52
Glu 233
OH
Leu 162
Tyr 62
Leu 165
Ser 112
Ile 51
H
OH
Asn 53
Ala 106
OH
Asp 197
Ala 50
OH
Trp 58
Trp 59
Gln 63
Val 107
Ser 108
Gly 104
Pro 54

<table>
<tr><td>7</td><td>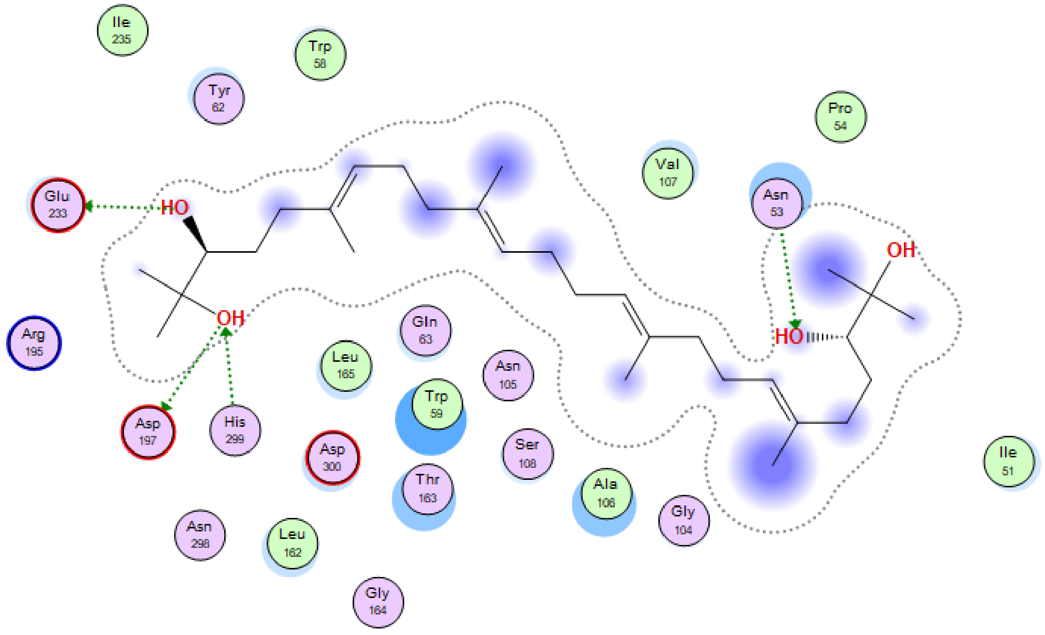
</td></tr>
<tr><td>8</td><td>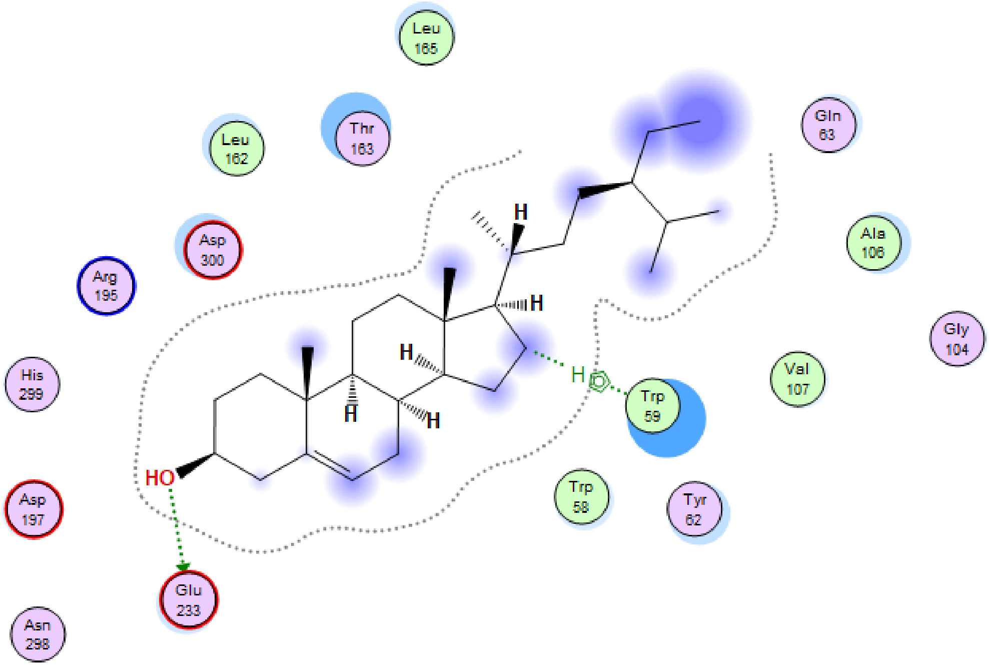
</td></tr>
</table>

<table>
<tr><td>9</td><td>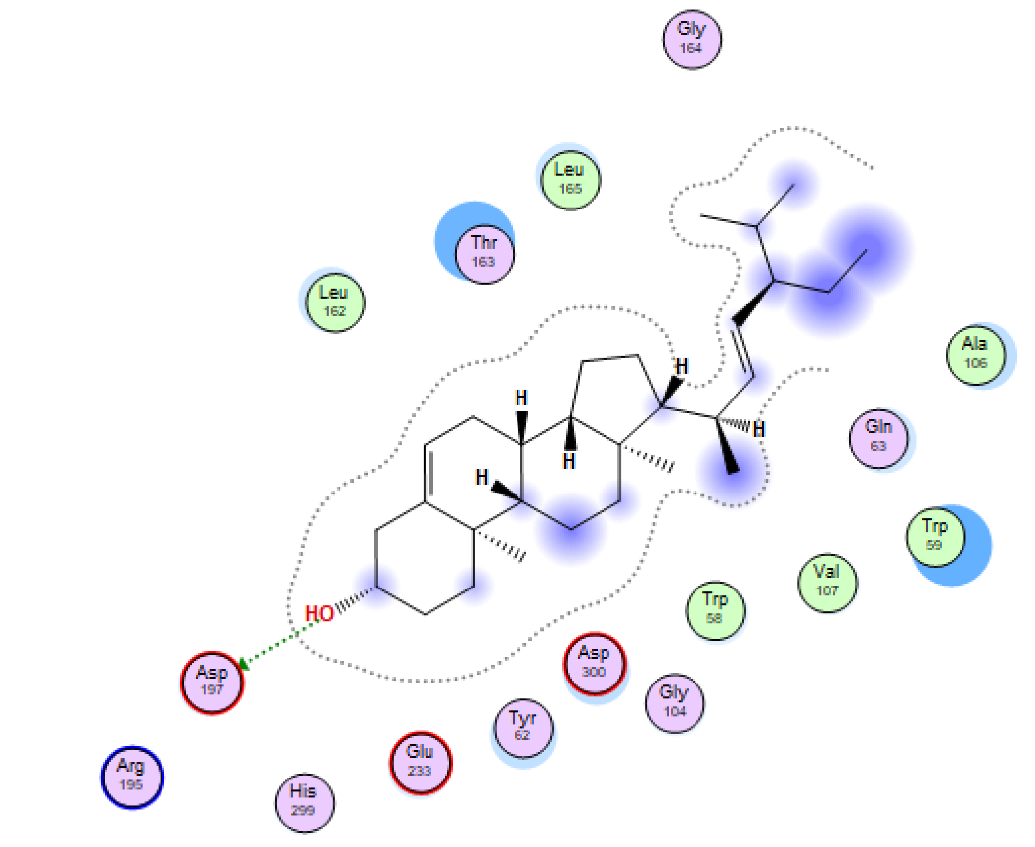
</td></tr>
<tr><td>10</td><td>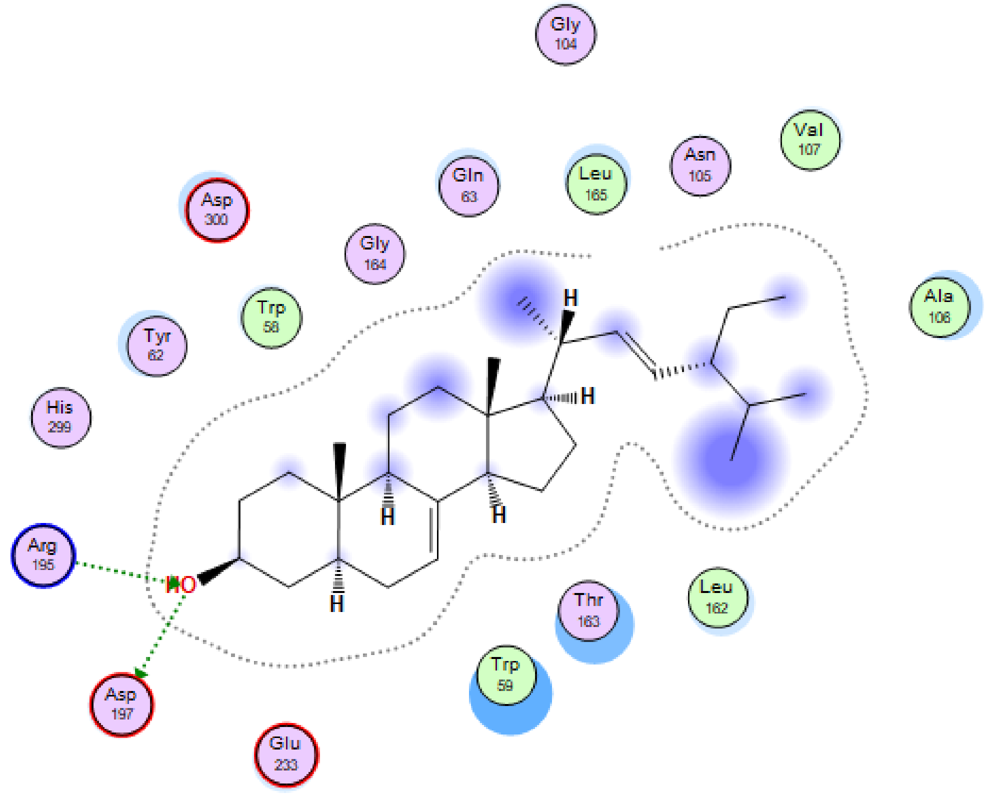
</td></tr>
</table>

<table>
<tr><td></td><td></td></tr>
<tr><td>11</td><td>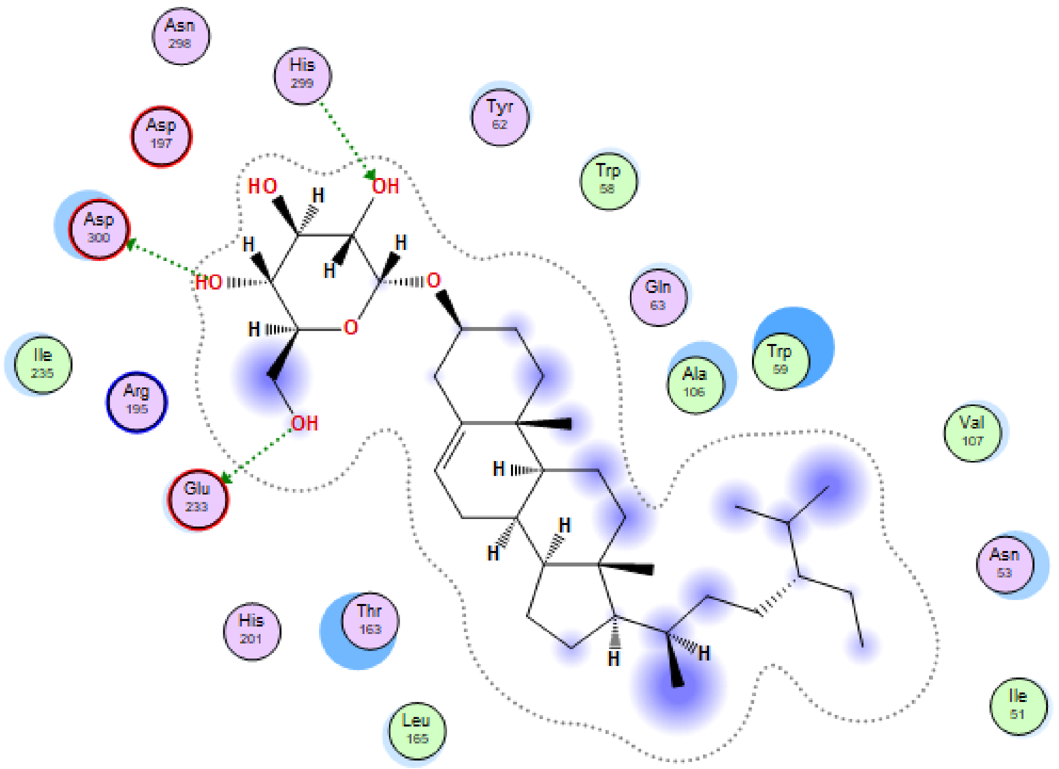
</td></tr>
<tr><td>12</td><td>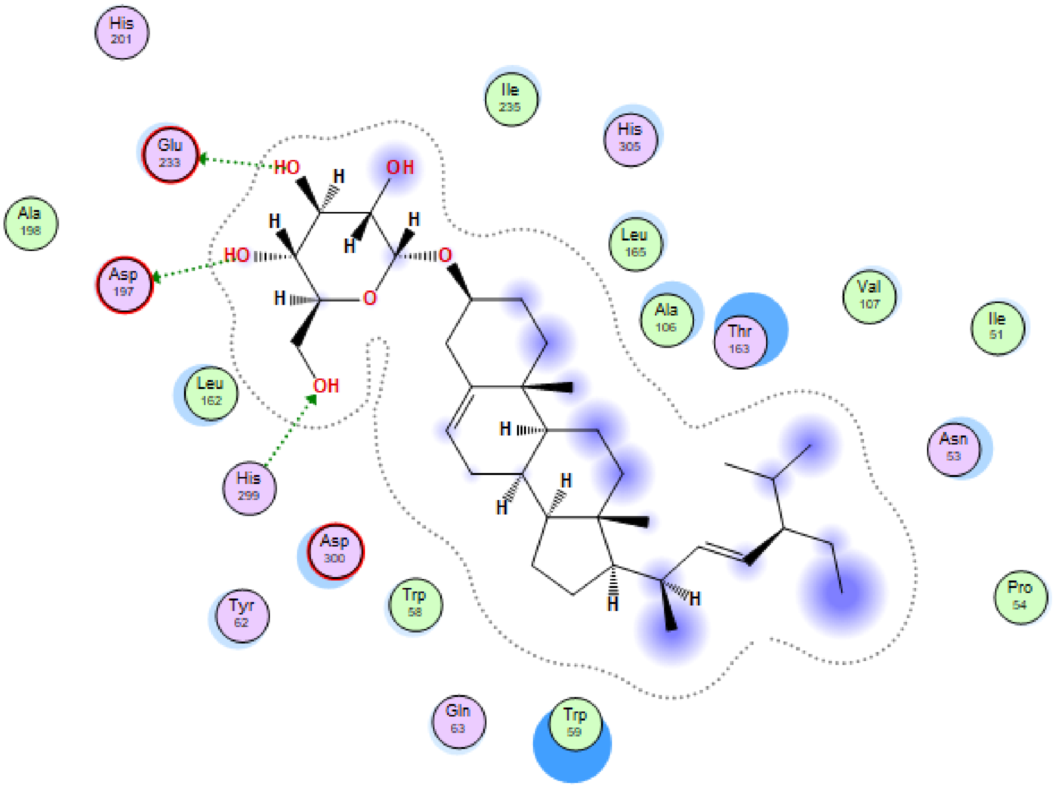
</td></tr>
</table>

**13**	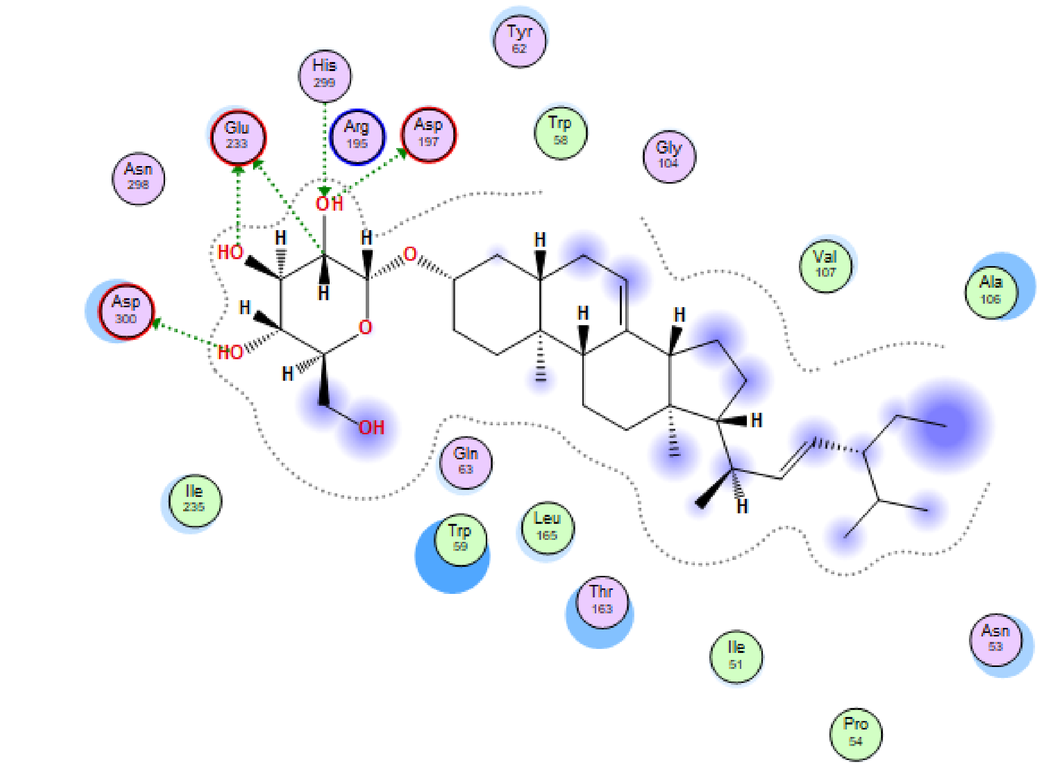Tyr 62<br>His 299<br>Glu 233<br>Arg 195<br>Asp 197<br>Trp 58<br>Gly 104<br>Asn 298<br>Val 107<br>Ala 106<br>Asp 300<br>Gln 63<br>Ile 235<br>Trp 59<br>Leu 165<br>Thr 163<br>Ile 51<br>Asn 53<br>Pro 54
**14**	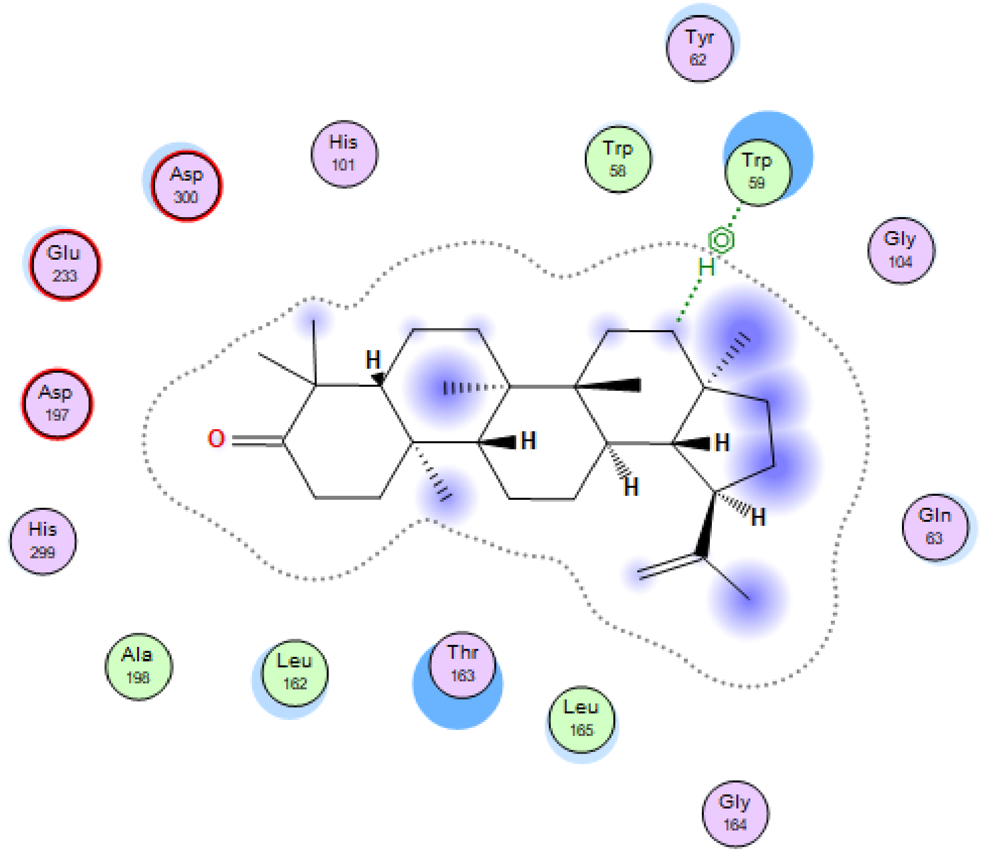Tyr 62<br>His 101<br>Asp 300<br>Trp 58<br>Trp 59<br>Glu 233<br>Gly 104<br>Asp 197<br>His 299<br>Gln 63<br>Ala 198<br>Leu 162<br>Thr 163<br>Leu 165<br>Gly 164

**15**	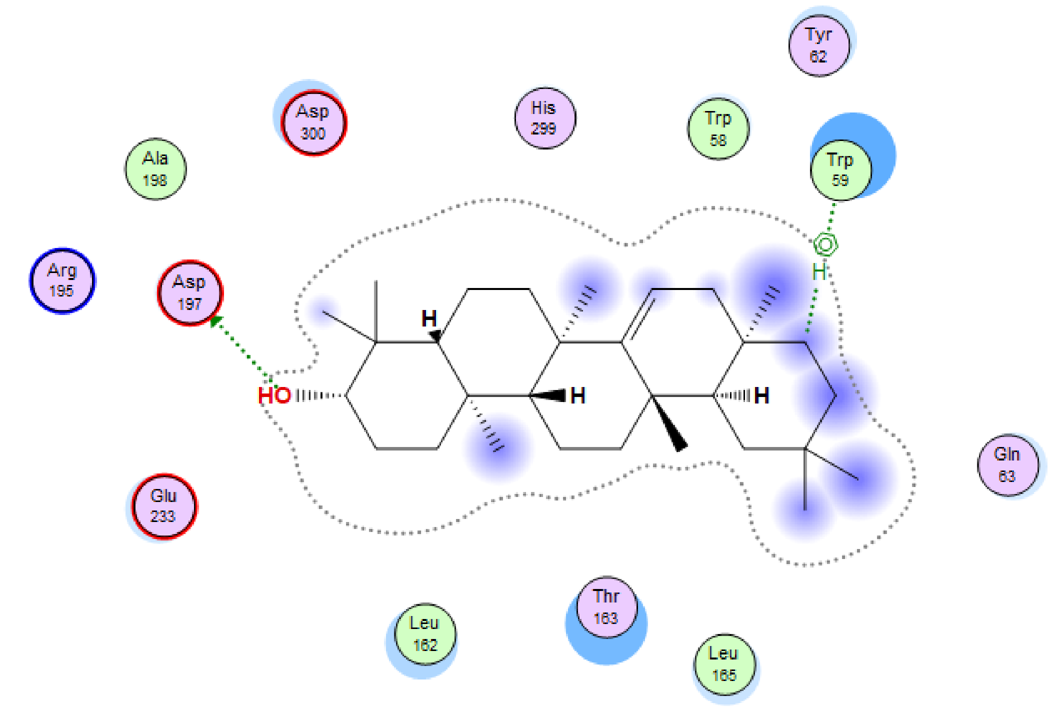
**16**	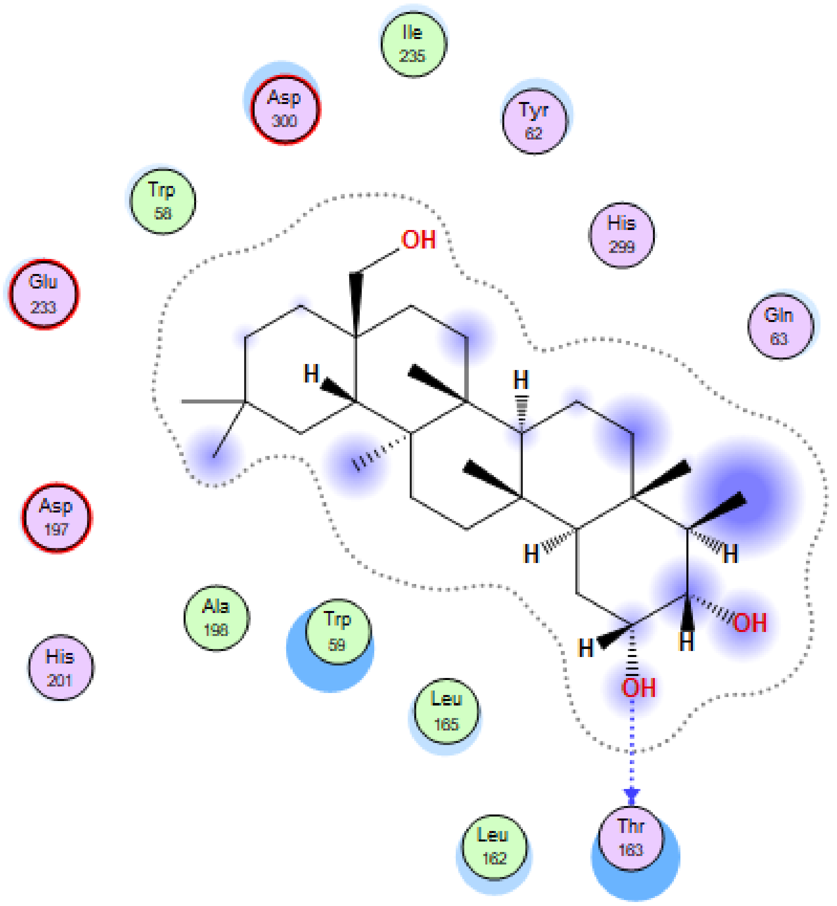

Tyr 62
Asp 300
His 299
Trp 58
Trp 59
Ala 198
Arg 195
Asp 197
HO
H
Gln 63
Glu 233
Leu 162
Thr 163
Leu 165
Ile 235
Tyr 62
Asp 300
Trp 58
His 299
OH
Glu 233
Gln 63
Asp 197
Ala 198
Trp 59
His 201
Leu 165
Leu 162
Thr 163

**17**	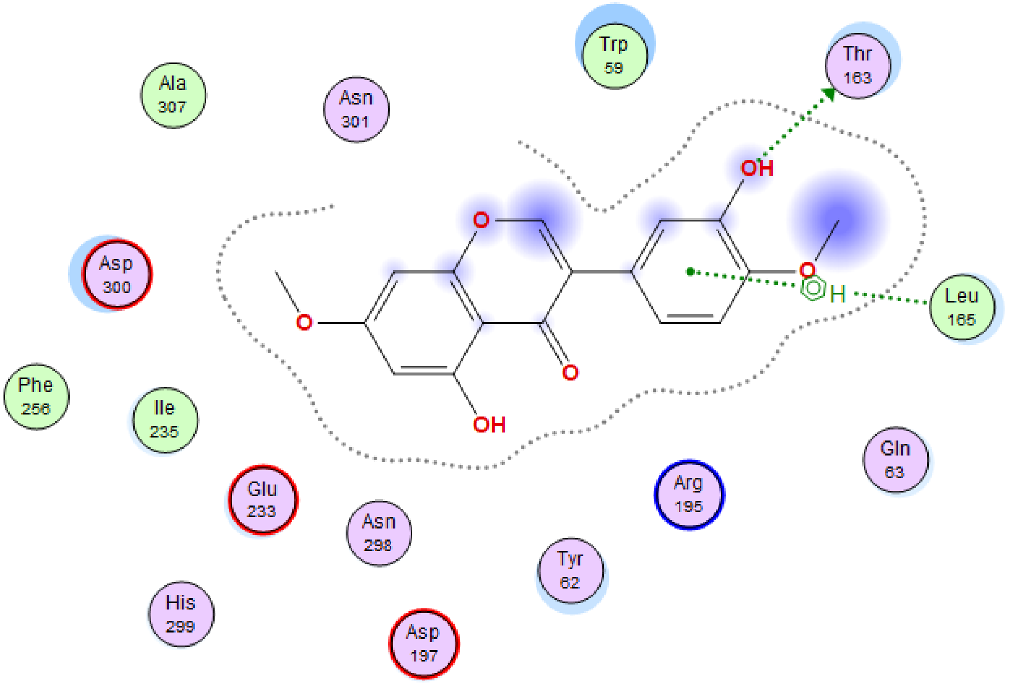Trp 59<br>Thr 163<br>Ala 307<br>Asn 301<br>Asp 300<br>Leu 165<br>Phe 256<br>Ile 235<br>Gln 63<br>Glu 233<br>Arg 195<br>Asn 298<br>Tyr 62<br>His 299<br>Asp 197
**Acarbose**	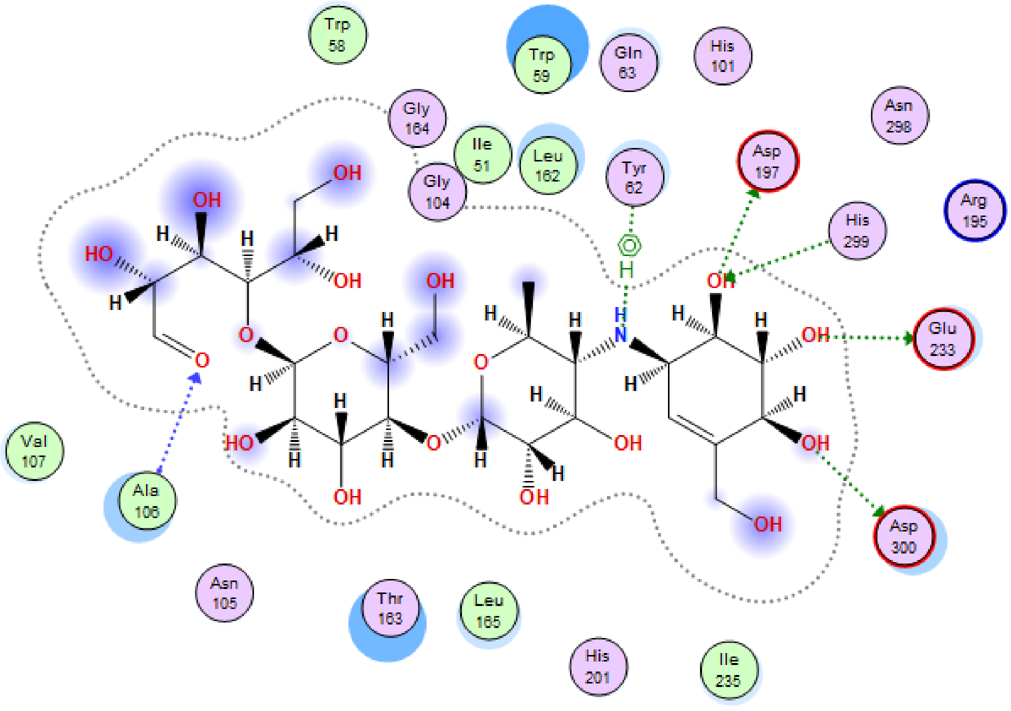Trp 58<br>Trp 59<br>Gln 63<br>His 101<br>Asn 298<br>Gly 164<br>Ile 51<br>Leu 162<br>Gly 104<br>Tyr 62<br>Asp 197<br>His 299<br>Arg 195<br>Glu 233<br>Val 107<br>Ala 106<br>Asp 300<br>Asn 105<br>Thr 163<br>Leu 165<br>His 201<br>Ile 235

Table S3: Docking energies with 2QLY for *α*-glucosidase and types of interaction involved

Ligands	Docking score (kcal/mol)	Amino acid, interaction type, distance and energy involved				
		Ligand	Receptor	Interaction	Distance (Å)	E (kcal/mol)
**1**	-10.35	O 1	O LEU 286 (A)	H-donor	3.12	-1.0
		C 8	OD2 ASP 777 (A)	H-donor	3.23	-0.9
		O 68	OD1 ASP 777 (A)	H-donor	2.76	-1.7
		O 74	SD MET 567 (A)	H-donor	3.33	-1.4
		O 68	N LEU 286 (A)	H-acceptor	3.04	-2.0
		O 78	N ILE 523 (A)	H-acceptor	2.94	-2.6
**2**	-9.79	C 39	SD MET 567 (A)	H-donor	3.55	-0.5
		O 44	OD2 ASP 777 (A)	H-donor	2.86	-2.6
		O 50	O GLY 533 (A)	H-donor	2.96	-1.1
		O 52	O ALA 285 (A)	H-donor	2.91	-2.3
		O 1	NZ LYS 776 (A)	H-acceptor	3.13	-3.9
		O 46	N LEU 286 (A)	H-acceptor	3.39	-0.8
**3**	-10.58	O 77	N MET 567 (A)	H-acceptor	2.89	-1.8
**4**	-10.58	O 60	O GLY 533 (A)	H-donor	2.99	-1.1
		O 62	O ALA 285 (A)	H-donor	2.82	-2.3
**5**	-10.20	O 61	O LEU 286 (A)	H-donor	3.00	-1.4
**6**	-9.93	O 36	O GLU 114 (A)	H-donor	3.17	-0.9
		O 81	SD MET 567 (A)	H-donor	3.08	-0.5
		O 87	O ALA 536 (A)	H-donor	3.14	-1.1
		O 87	N MET 567 (A)	H-acceptor	3.28	-0.7
**6**	-10.75	O 80	SD MET 567 (A)	H-donor	2.98	-0.5
		O 86	O ALA 285 (A)	H-donor	2.68	-2.2
		O 88	5-ring HIS 645 (A)	H-pi	3.45	-0.6
**16**	-0.99	O 84	N SER 288 (A)	H-acceptor	3.19	-1.8
**15**	+1.00	O 70	SD MET 567 (A)	H-donor	2.85	-1.4
**8**	-6.98	O 3	NZ LYS 534 (A)	H-acceptor	3.29	-0.5
**9**	-5.75	NI	NI	NI	NI	NI
**10**	-6.33	NI	NI	NI	NI	NI
**11**	-7.33	O 93	NZ LYS 513 (A)	H-acceptor	3.11	-3.6
**12**	-8.08	C 88	SD MET 567 (A)	H-donor	3.73	-0.6
		O 97	O SER 521 (A)	H-donor	2.78	-1.6
		O 97	N SER 288 (A)	H-acceptor	3.47	-0.5
**13**	-6.85	C 88	SD MET 567 (A)	H-donor	3.98	-0.5
		O 91	O ALA 285 (A)	H-donor	2.70	-1.4
		O 93	SD MET 567 (A)	H-donor	3.46	-0.5
		O 97	O ARG 520 (A)	H-donor	3.31	-0.5
		O 95	N ILE 523 (A)	H-acceptor	2.75	-2.6
**14**	-2.31	NI	NI	NI	NI	NI
**17**	-7.78	O 24	SD MET 567 (A)	H-donor	3.18	-2.4
**Acarbose**	-9.42	N 12	O ARG 520 (A)	H-donor	3.00	-0.8
		O 65	O ALA 509 (A)	H-donor	2.90	-0.7
		O 75	O LEU 286 (A)	H-donor	2.60	-1.4
		O 80	SD MET 567 (A)	H-donor	3.90	-1.1
		O 18	NZ LYS 776 (A)	H-acceptor	3.06	-1.3
		O 84	N ILE 523 (A)	H-acceptor	2.66	-2.6

NI = No visible interactions their docking score may be due to their hydrophobic interaction

Table S4: 2D interactions with 2QLY for α-glucosidase

Ligands	2D
**1**	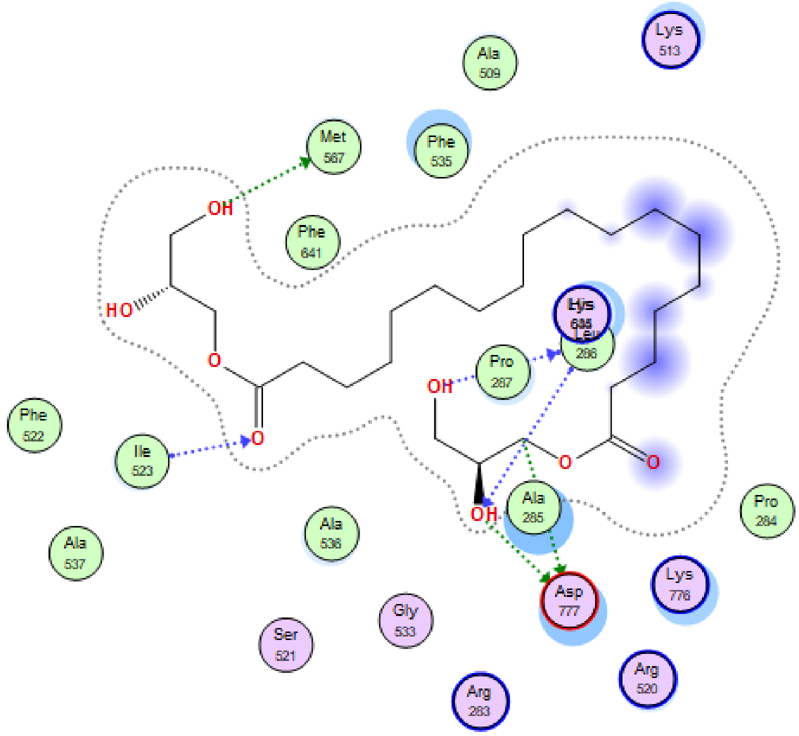
**2**	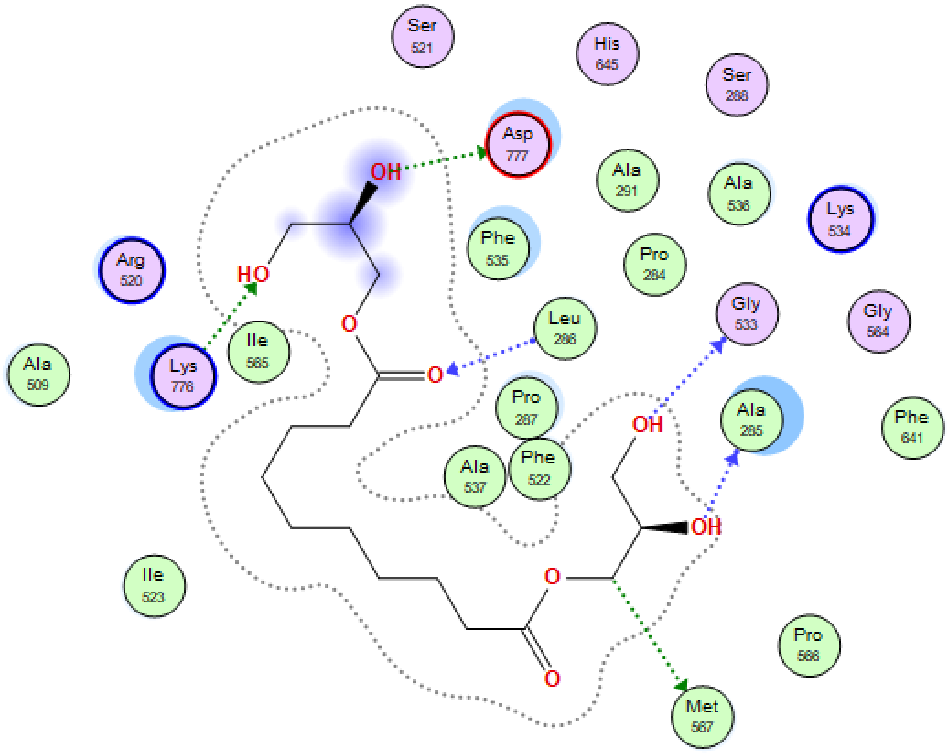

3

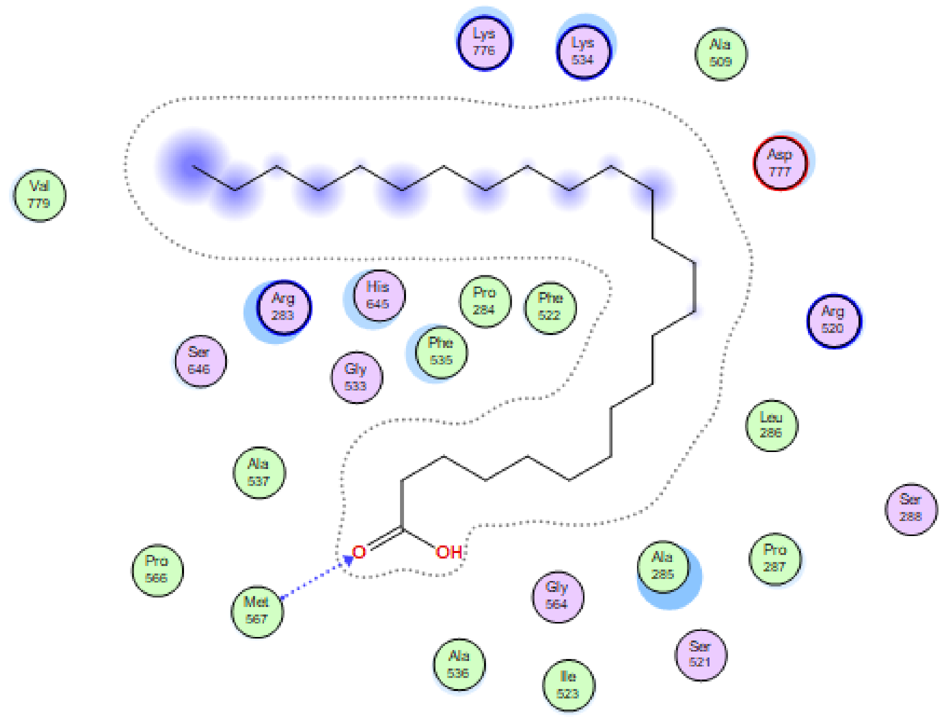
Lys 776
Lys 534
Ala 509
Asp 777
Val 779
Arg 283
His 645
Pro 284
Phe 522
Arg 520
Ser 646
Gly 533
Phe 535
Leu 286
Ala 537
Ser 288
O
OH
Pro 566
Met 567
Gly 564
Ala 285
Pro 287
Ala 536
Ile 523
Ser 521

4

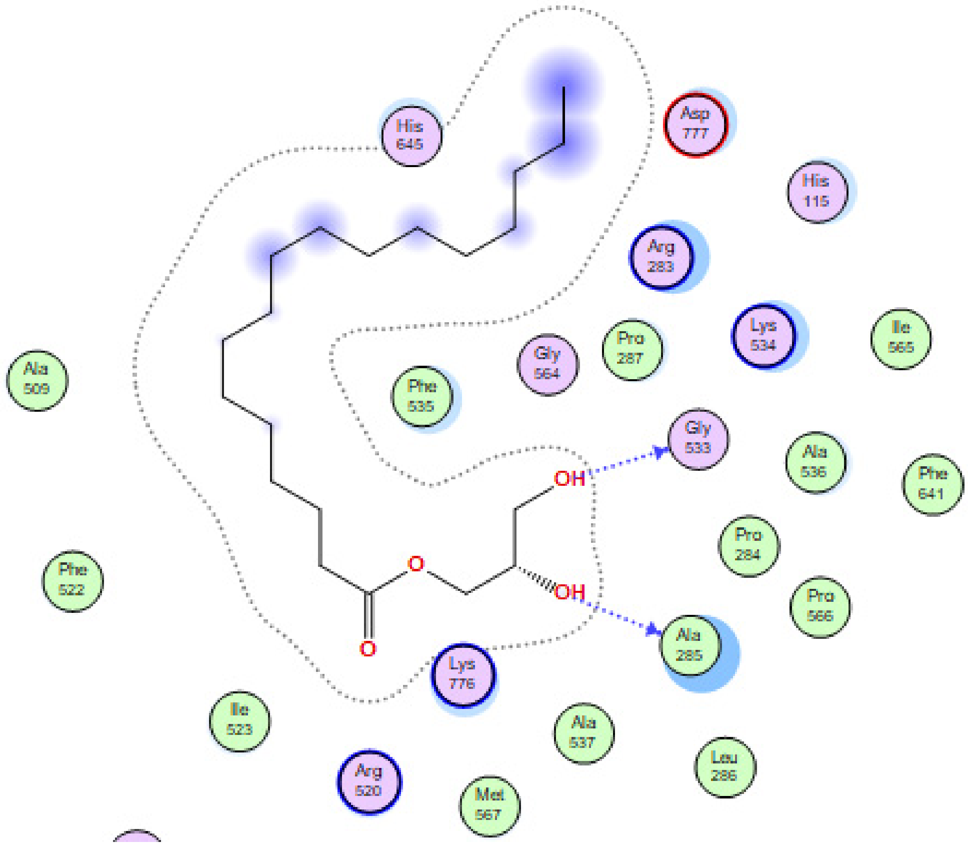
His 645
Asp 777
His 115
Arg 283
Ala 509
Gly 564
Pro 287
Lys 534
Ile 565
Phe 535
Gly 533
Ala 536
Phe 641
OH
Pro 284
O
OH
Phe 522
Pro 566
O
Ala 285
Lys 776
Ile 523
Ala 537
Leu 286
Arg 520
Met 567

**5**	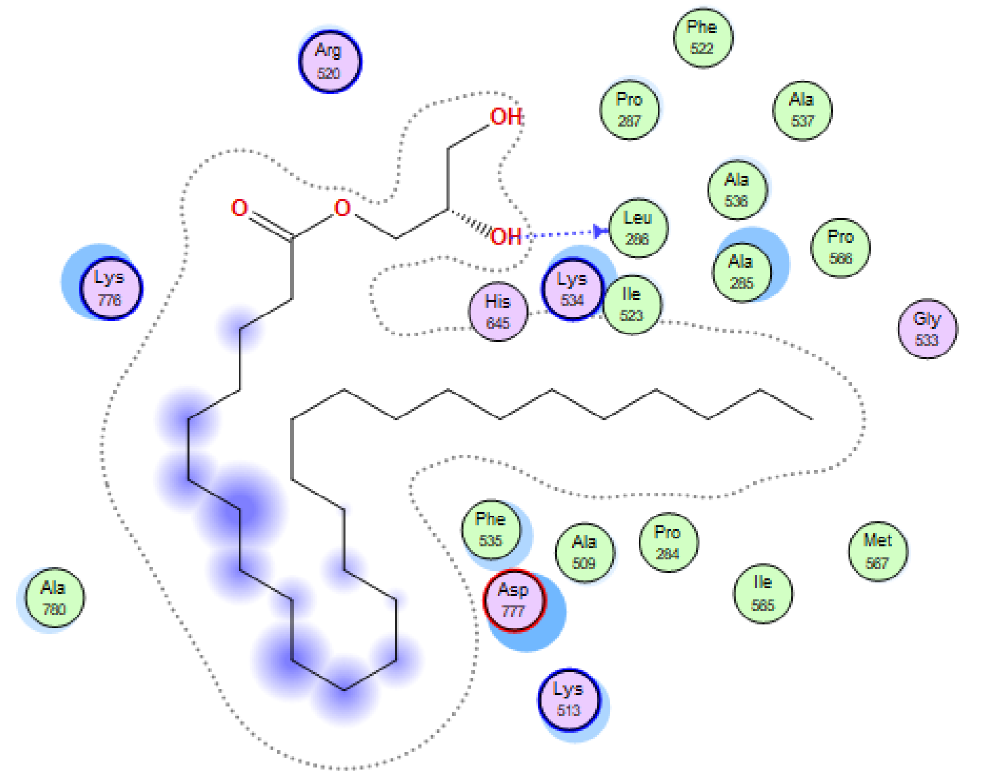 Arg 520<br>Phe 522<br>Pro 287<br>Ala 537<br>OH<br>O<br>O<br>OH<br>Leu 286<br>Ala 536<br>Lys 776<br>Lys 534<br>Pro 566<br>Ala 285<br>His 645<br>Ile 523<br>Gly 533<br>Phe 535<br>Ala 509<br>Pro 284<br>Met 567<br>Ile 565<br>Ala 780<br>Asp 777<br>Lys 513
**6**	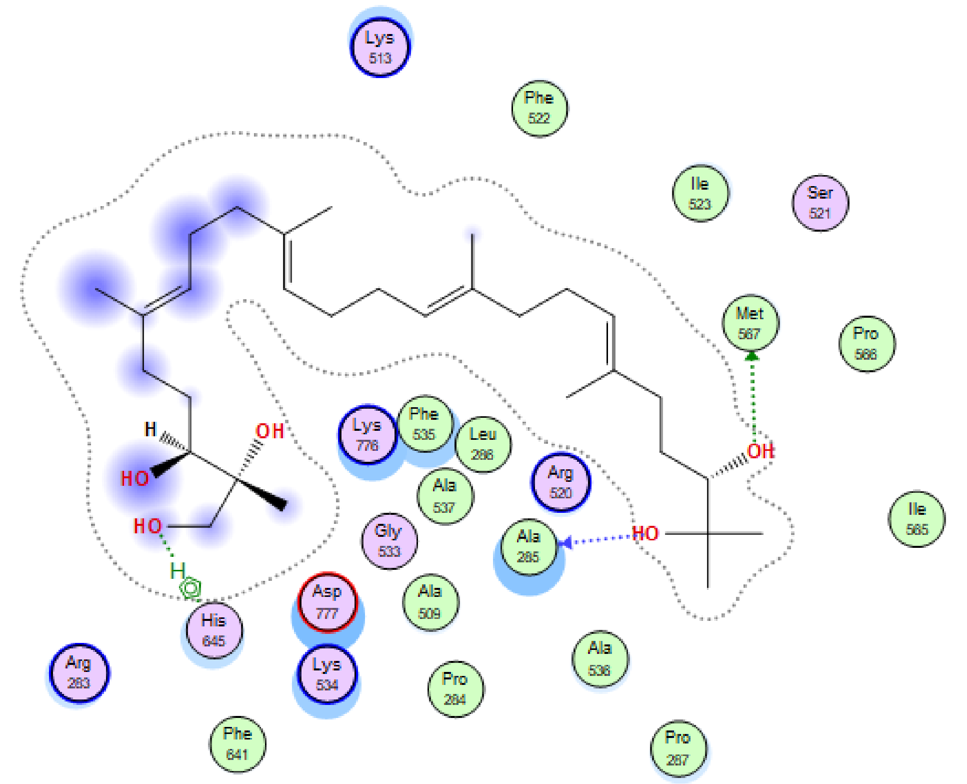 Lys 513<br>Phe 522<br>Ile 523<br>Ser 521<br>Met 567<br>Pro 566<br>H<br>OH<br>HO<br>Lys 776<br>Phe 535<br>Leu 286<br>Ala 537<br>Arg 520<br>OH<br>HO<br>Gly 533<br>Ala 285<br>HO<br>Ile 565<br>H<br>Asp 777<br>Ala 509<br>His 645<br>Ala 536<br>Arg 283<br>Lys 534<br>Pro 284<br>Phe 641<br>Pro 287

**7**	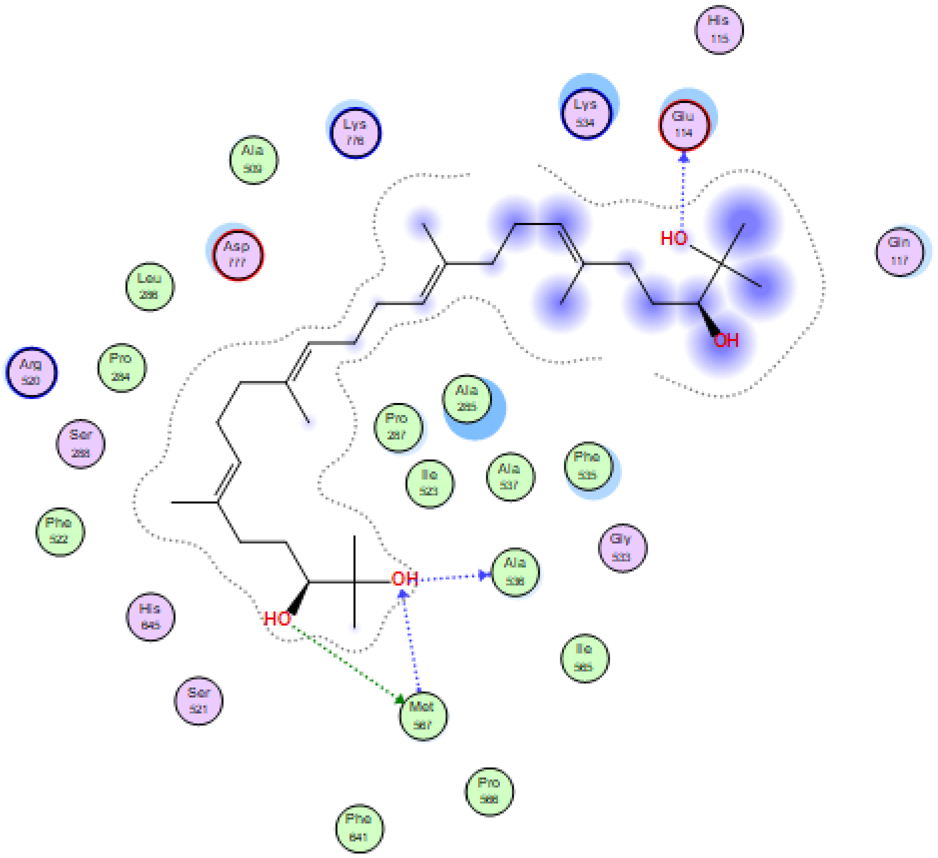
**8**	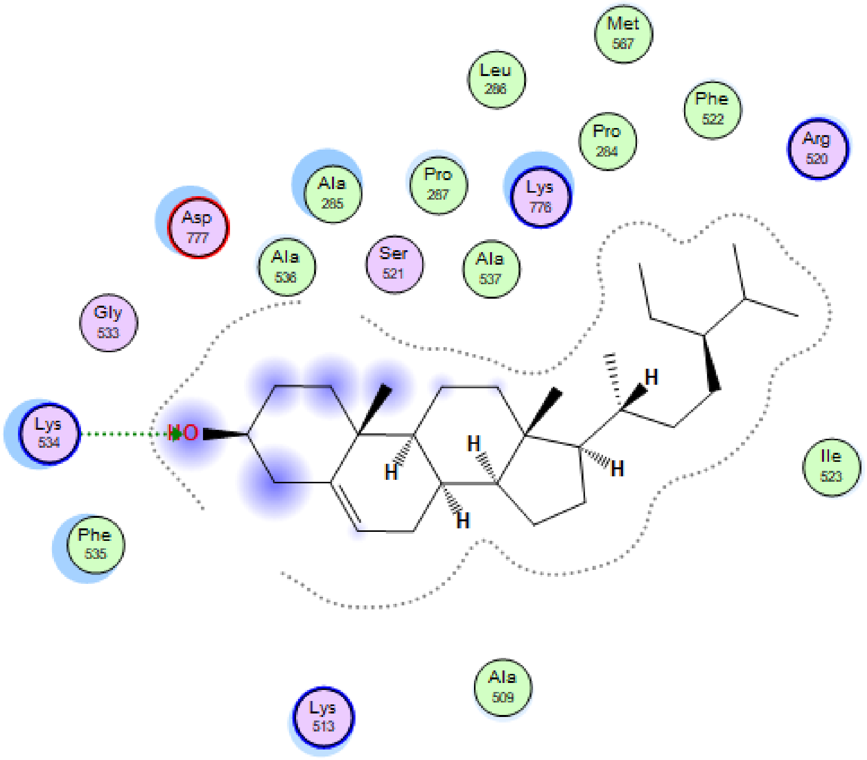

His
Lys
Glu
Lys
Ala
Asp
Leu
Arg
Pro
Ser
Gln
HO
OH
Ala
Pro
Ile
Ala
Phe
Gly
Phe
OH
Ala
HO
His
Ser
Ile
Met
Pro
Phe

Met 567
Leu 286
Phe 522
Pro 284
Arg 520
Ala 285
Pro 287
Lys 776
Asp 777
Ala 536
Ser 521
Ala 537
Gly 533
Lys 534
HO
H
H
H
H
H
Ile 523
Phe 535
Ala 509
Lys 513

**9**	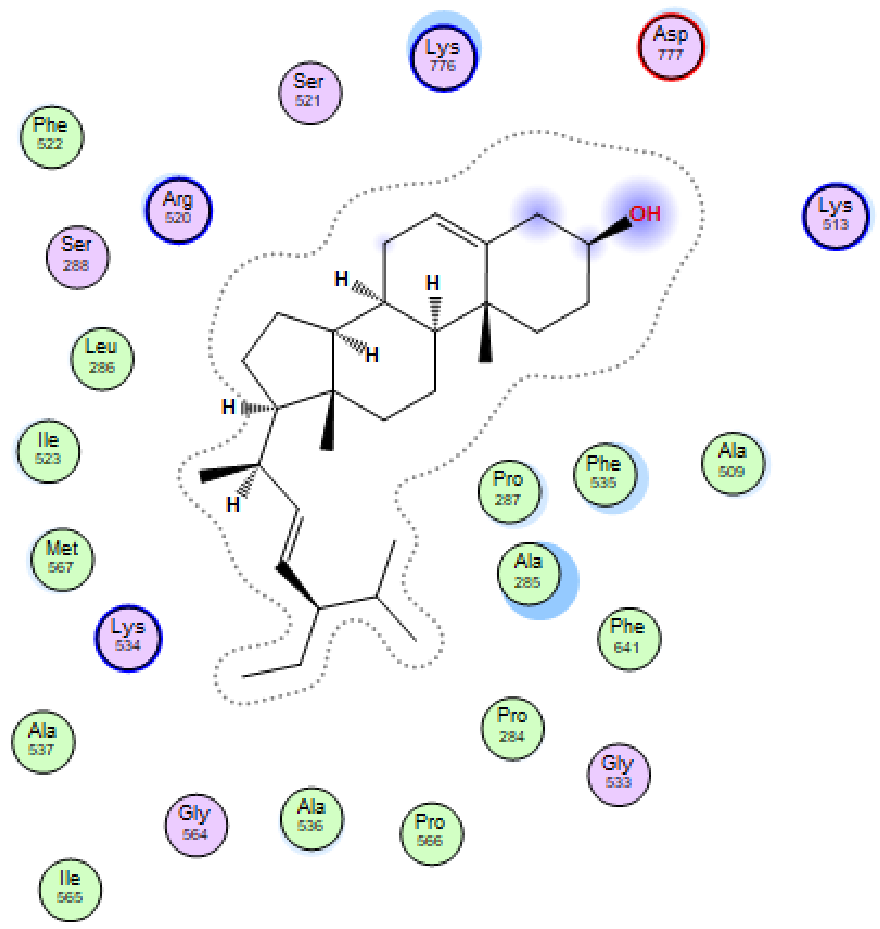Phe 522<br>Ser 521<br>Lys 776<br>Asp 777<br>Arg 520<br>Ser 288<br>OH<br>Lys 513<br>Leu 286<br>Ile 523<br>Pro 287<br>Phe 535<br>Ala 509<br>Met 567<br>Ala 285<br>Lys 534<br>Phe 641<br>Pro 284<br>Ala 537<br>Gly 533<br>Gly 564<br>Ala 536<br>Pro 566<br>Ile 565
**10**	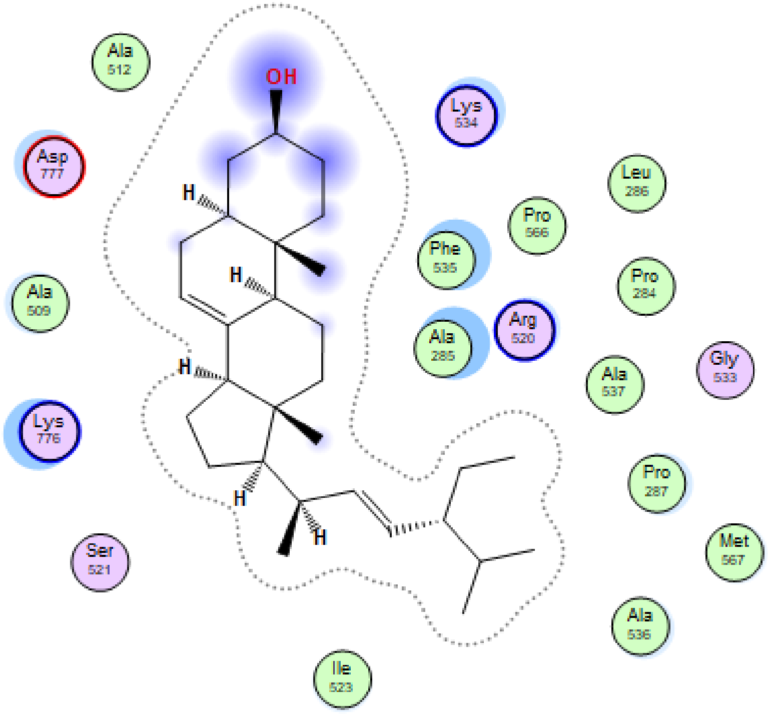Ala 512<br>OH<br>Lys 534<br>Asp 777<br>Leu 286<br>Pro 566<br>Phe 535<br>Ala 509<br>Pro 284<br>Ala 285<br>Arg 520<br>Ala 537<br>Gly 533<br>Lys 776<br>Pro 287<br>Ser 521<br>Met 567<br>Ala 536<br>Ile 523

**11**	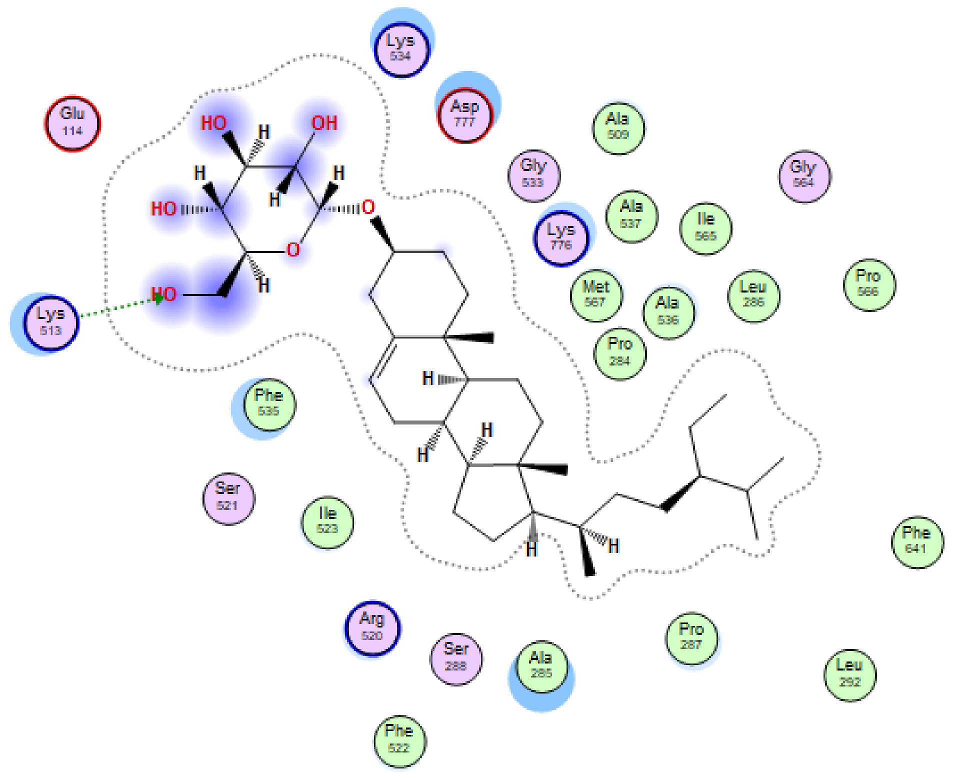Lys 534<br>Glu 114<br>Asp 777<br>Ala 509<br>Gly 533<br>Gly 564<br>Ala 537<br>Ile 565<br>Lys 776<br>Pro 566<br>Met 567<br>Leu 286<br>Ala 536<br>Lys 513<br>Pro 284<br>Phe 535<br>Ser 521<br>Ile 523<br>Phe 641<br>Arg 520<br>Ser 288<br>Ala 285<br>Pro 287<br>Leu 292<br>Phe 522
**12**	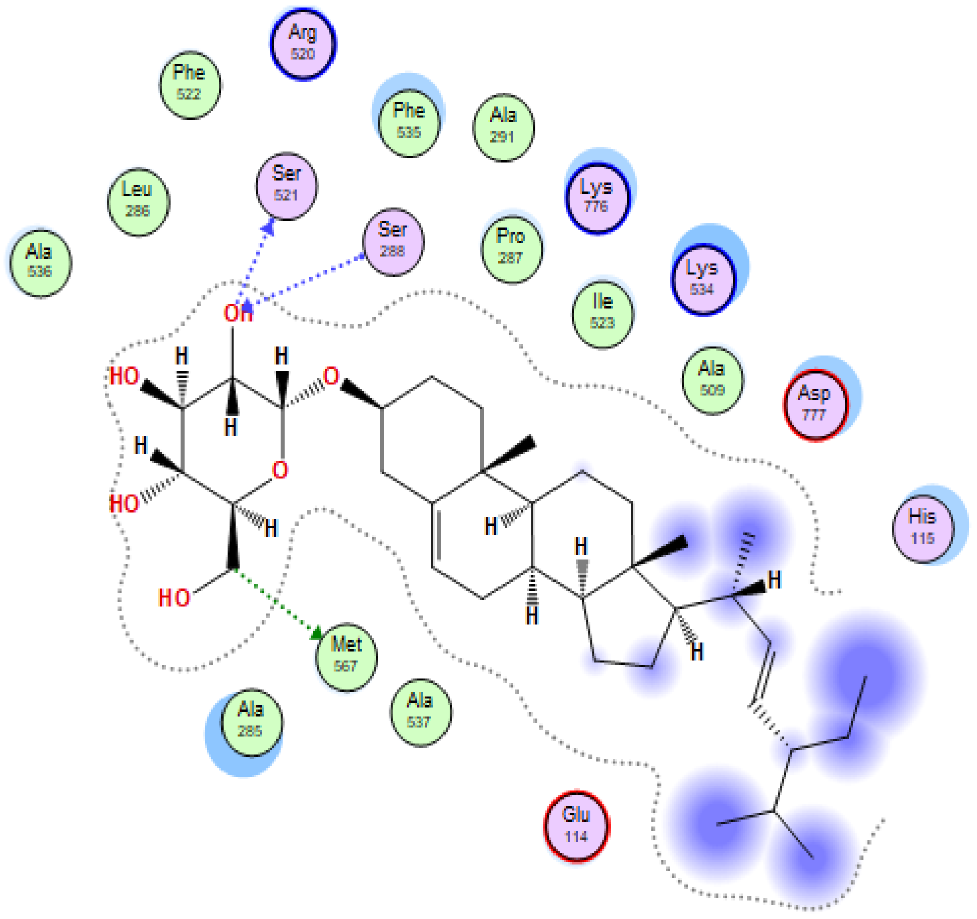Arg 520<br>Phe 522<br>Phe 535<br>Ala 291<br>Leu 286<br>Ser 521<br>Lys 776<br>Ala 536<br>Ser 288<br>Pro 287<br>Lys 534<br>Ile 523<br>Ala 509<br>Asp 777<br>His 115<br>Met 567<br>Ala 285<br>Ala 537<br>Glu 114

<table>
<tr><td>13</td><td>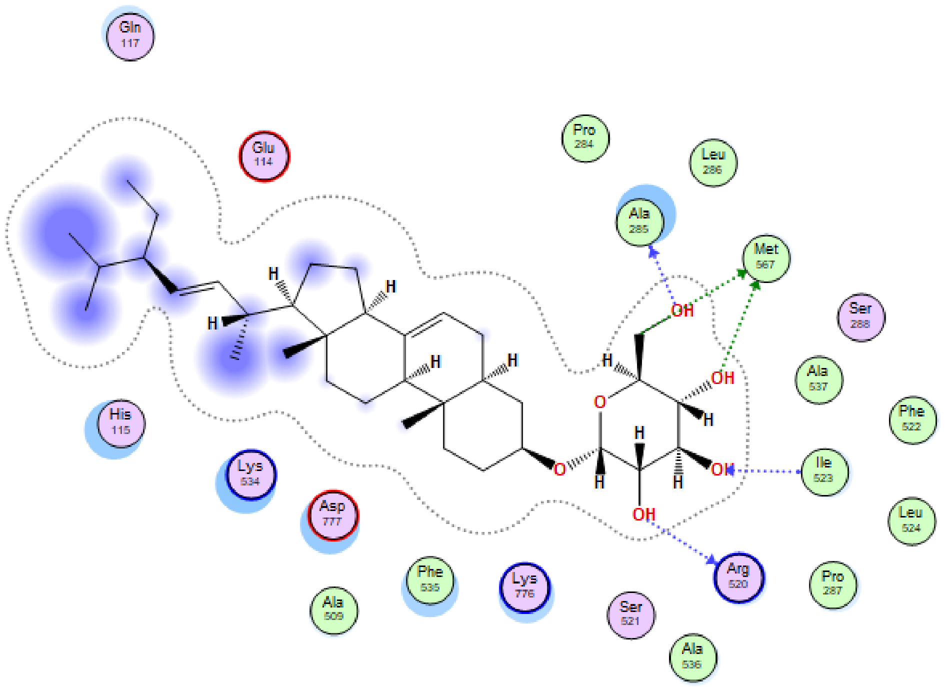
</td></tr>
<tr><td>14</td><td>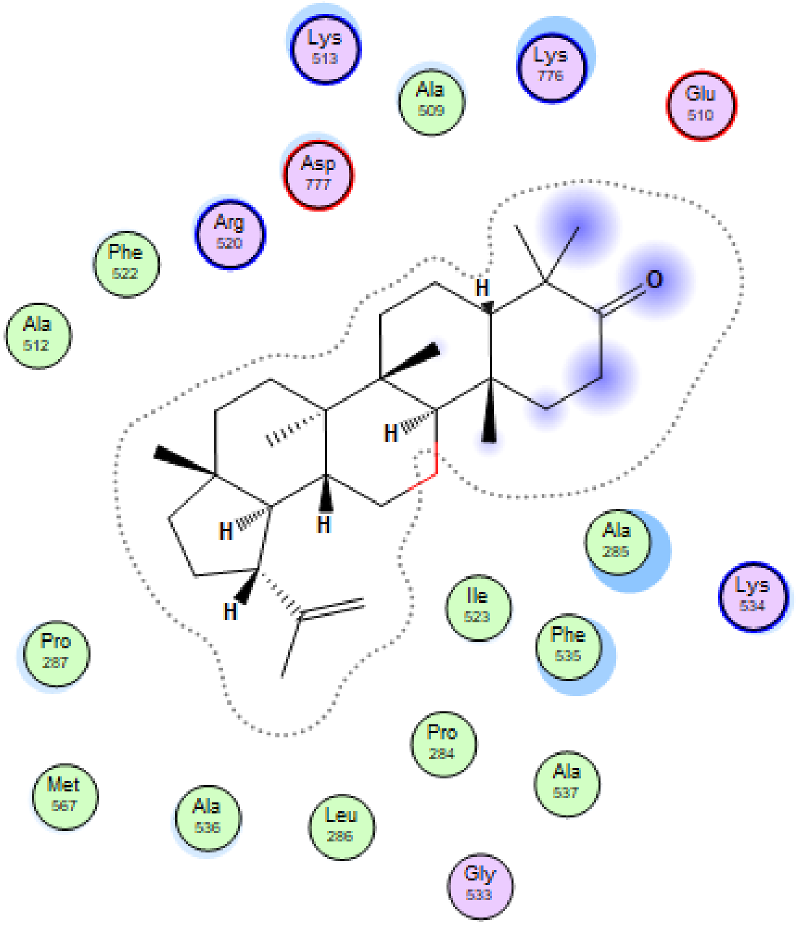
</td></tr>
</table>

**15**	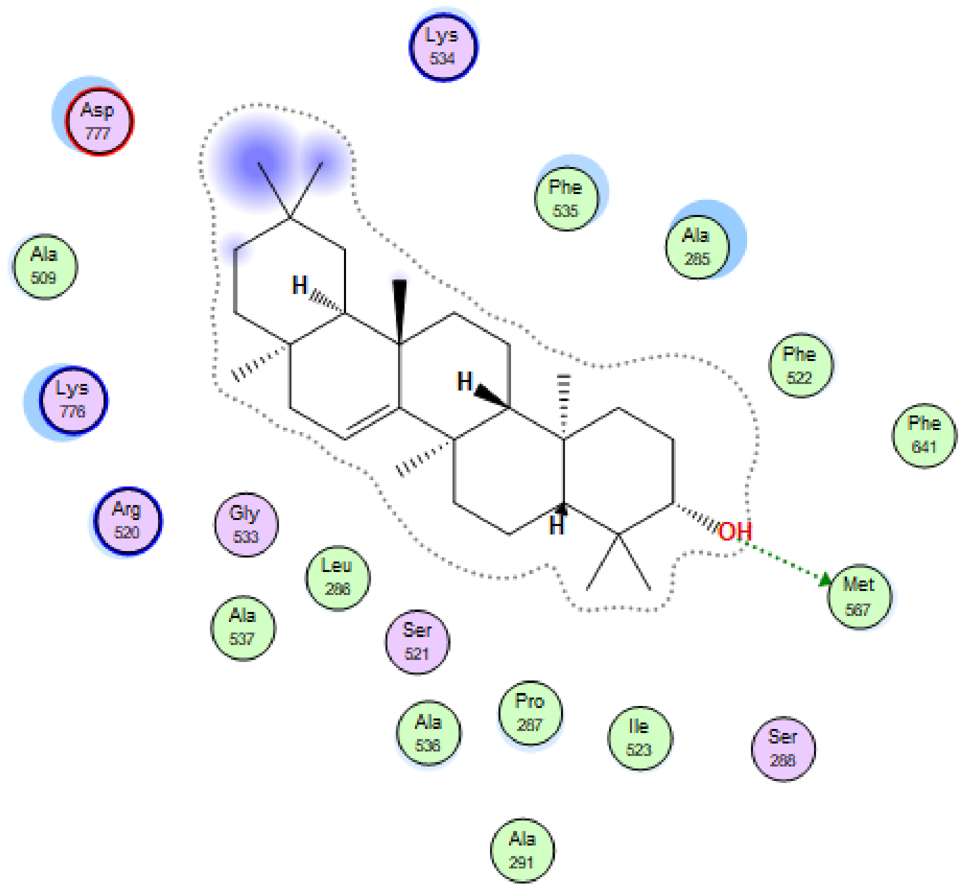Lys 534<br>Asp 777<br>Phe 535<br>Ala 285<br>Ala 509<br>H<br>H<br>Phe 522<br>Lys 776<br>Phe 641<br>Arg 520<br>Gly 533<br>H<br>OH<br>Leu 286<br>Met 567<br>Ala 537<br>Ser 521<br>Ala 536<br>Pro 287<br>Ile 523<br>Ser 288<br>Ala 291
**16**	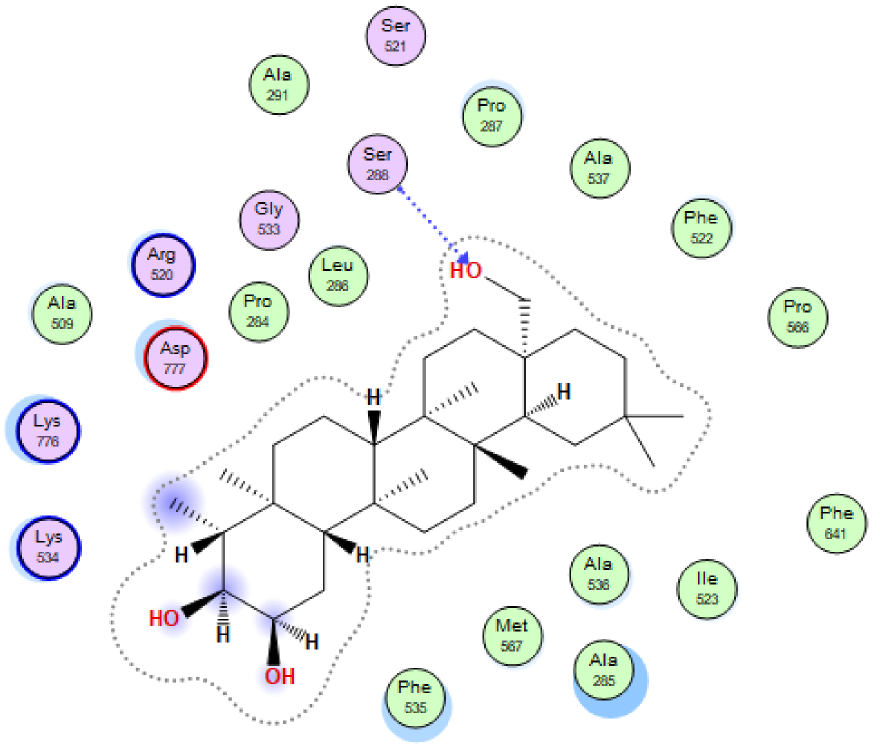Ser 521<br>Ala 291<br>Pro 287<br>Ser 288<br>Ala 537<br>Gly 533<br>Phe 522<br>Arg 520<br>Leu 286<br>HO<br>Ala 509<br>Pro 284<br>Pro 566<br>Asp 777<br>H<br>H<br>Lys 776<br>Phe 641<br>H<br>H<br>Lys 534<br>Ala 536<br>Ile 523<br>HO<br>H<br>H<br>Met 567<br>OH<br>Ala 285<br>Phe 535

<table>
<tr><td>17</td><td>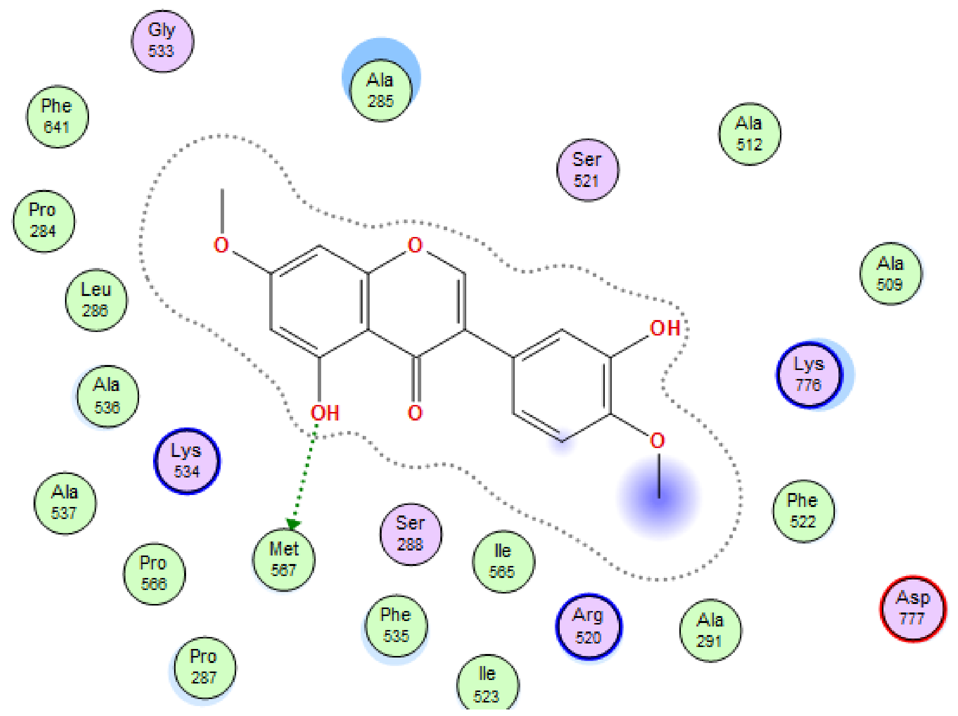
</td></tr>
<tr><td>Acarbose</td><td>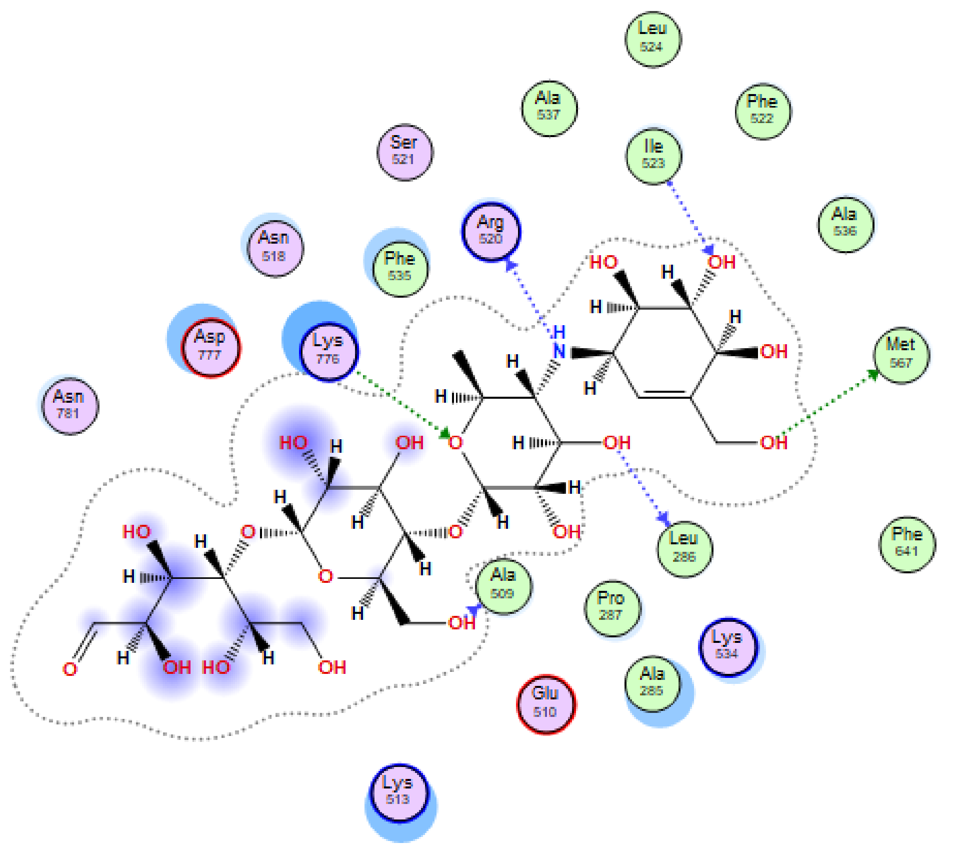
</td></tr>
</table>

Table S5: Adsorption, Distribution, Metabolism and Excretion (ADME) properties of compounds **1–7** and **11–13**

	**Water Solubility**									
	**1**	**2**	**3**	**4**	**5**	**6**	**7**	**11**	**12**	**13**
**Log *S* (ESOL)**	-3.88	-0.99	-8.25	-5.05	-8.67	-8.67	-8.67	-7.70	-7.26	-7.10
**Solubility**	6.09e-02 mg/ml ; 1.32e-04 mol/l	3.63e+01 mg/ml ; 1.04e-01 mol/l	2.13e-06 mg/ml ; 5.57e-09 mol/l	3.07e-03 mg/ml ; 8.91e-06 mol/l	1.03e-06 mg/ml ; 2.12e-09 mol/l	1.03e-06 mg/ml ; 2.12e-09 mol/l	1.03e-06 mg/ml ; 2.12e-09 mol/l	1.15e-05 mg/ml ; 2.00e-08 mol/l	3.15e-05 mg/ml ; 5.49e-08 mol/l	4.53e-05 mg/ml ; 7.88e-08 mol/l
**Class**	Soluble	Very soluble	Poorly soluble	Moderately soluble	Poorly soluble	Poorly soluble	Poorly soluble	Poorly soluble	Poorly soluble	Poorly soluble
**Log *S* (Ali)**	-7.00	-2.51	-12.79	-8.08	-13.71	-13.71	-13.71	-9.67	-8.86	-8.60
**Solubility**	4.58e-05 mg/ml ; 9.91e-08 mol/l	1.08e+00 mg/ml ; 3.08e-03 mol/l	6.25e-11 mg/ml ; 1.63e-13 mol/l	2.85e-06 mg/ml ; 8.28e-09 mol/l	9.53e-12 mg/ml ; 1.97e-14 mol/l	9.53e-12 mg/ml ; 1.97e-14 mol/l	9.53e-12 mg/ml ; 1.97e-14 mol/l	1.23e-07 mg/ml ; 2.14e-10 mol/l	7.92e-07 mg/ml ; 1.38e-09 mol/l	1.44e-06 mg/ml ; 2.51e-09 mol/l
**Class**	Poorly soluble	Soluble	Insoluble	Poorly soluble	Insoluble	Insoluble	Insoluble	Poorly soluble	Poorly soluble	Poorly soluble
**Log *S* (SILICOS-IT)**	-5.01	-1.87	-8.89	-5.70	-9.62	-9.62	-9.62	-4.40	-3.69	-3.69
**Solubility**	4.48e-03 mg/ml ; 9.68e-06 mol/l	4.68e+00 mg/ml ; 1.34e-02 mol/l	4.96e-07 mg/ml ; 1.30e-09 mol/l	6.86e-04 mg/ml ; 1.99e-06 mol/l	1.16e-07 mg/ml ; 2.39e-10 mol/l	1.16e-07 mg/ml ; 2.39e-10 mol/l	1.16e-07 mg/ml ; 2.39e-10 mol/l	2.28e-02 mg/ml ; 3.94e-05 mol/l	1.18e-01 mg/ml ; 2.06e-04 mol/l	1.18e-01 mg/ml ; 2.06e-04 mol/l
**Class**	Moderately soluble	Soluble	Poorly soluble	Moderately soluble	Poorly soluble	Poorly soluble	Poorly soluble	Moderately soluble	Soluble	Soluble
	**Pharmacokinetics**									
	**1**	**2**	**3**	**4**	**5**	**6**	**7**	**11**	**12**	**13**
**GI absorption**	High	High	Low	High	Low	Low	Low	Low	High	High
**BBB permeant**	No	No	No	Yes	No	No	No	No	No	No
**P-gp substrate**	Yes	No	No	Yes	No	No	No	No	Yes	Yes
**CYP1A2 inhibitor**	No	No	Yes	Yes	No	No	No	No	No	No
**CYP2C19 inhibitor**	Yes	No	No	No	No	No	No	No	No	No
**CYP2C9 inhibitor**	No	No	No	No	No	No	No	No	No	No
**CYP2D6 inhibitor**	No	No	No	Yes	No	No	No	No	No	No

**CYP3A4 inhibitor**	No	No	No	No	No	No	No	No	No	No
**Log K_p (skin permeation)**	-5.94 cm/s	-8.33 cm/s	-0.11 cm/s	-3.52 cm/s	-0.53 cm/s	-0.53 cm/s	-0.53 cm/s	-4.32 cm/s	-4.86 cm/s	-5.04 cm/s
	**Druglikeness**									
	**1**	**2**	**3**	**4**	**5**	**6**	**7**	**11**	**12**	**13**
**Lipinski**	Yes; 0 violation	Yes; 0 violation	Yes; 1 violation: MLOGP>4.15	Yes; 0 violation	Yes; 1 violation: MLOGP>4.15	Yes; 1 violation: MLOGP>4.15	Yes; 1 violation: MLOGP>4.15	Yes; 1 violation: MW>500	Yes; 1 violation: MW>500	Yes; 1 violation: MW>500
**Ghose**	No; 1 violation: #atoms>70	Yes	No; 2 violations: WLOGP>5.6, #atoms>70	Yes	No; 4 violations: MW>480, WLOGP>5.6, MR>130, #atoms>70	No; 4 violations: MW>480, WLOGP>5.6, MR>130, #atoms>70	No; 4 violations: MW>480, WLOGP>5.6, MR>130, #atoms>70	No; 4 violations: MW>480, WLOGP>5.6, MR>130, #atoms>70	No; 4 violations: MW>480, WLOGP>5.6, MR>130, #atoms>70	No; 4 violations: MW>480, WLOGP>5.6, MR>130, #atoms>70
**Veber**	No; 1 violation: Rotors>10	No; 1 violation: Rotors>10	No; 1 violation: Rotors>10	No; 1 violation: Rotors>10	No; 1 violation: Rotors>10	No; 1 violation: Rotors>10	No; 1 violation: Rotors>10	Yes	Yes	Yes
**Egan**	No; 1 violation: TPSA>131.6	No; 1 violation: TPSA>131.6	No; 1 violation: WLOGP>5.88	Yes	No; 1 violation: WLOGP>5.88	No; 1 violation: WLOGP>5.88	No; 1 violation: WLOGP>5.88	Yes	Yes	Yes
**Muegge**	No; 1 violation: Rotors>15	No; 1 violation: Rotors>15	No; 2 violations: XLOGP3>5, Rotors>15	No; 2 violations: XLOGP3>5, Rotors>15	No; 2 violations: XLOGP3>5, Rotors>15	No; 2 violations: XLOGP3>5, Rotors>15	No; 2 violations: XLOGP3>5, Rotors>15	No; 1 violation: XLOGP3>5	No; 1 violation: XLOGP3>5	No; 1 violation: XLOGP3>5
**Bioavailability Score**	0.55	0.55	0.85	0.55	0.55	0.55	0.55	0.55	0.55	0.55
	**Medicinal Chemistry**									
	**1**	**2**	**3**	**4**	**5**	**6**	**7**	**11**	**12**	**13**
**PAINS**	0 alert	0 alert	0 alert	0 alert	0 alert	0 alert	0 alert	0 alert	0 alert	0 alert
**Brenk**	1 alert: more_than_2_esters	1 alert: more_than_2_esters	0 alert	0 alert	0 alert	0 alert	0 alert	1 alert: isolated_alkene	1 alert: isolated_alkene	1 alert: isolated_alkene
**Lead-likeness**	No; 3 violations: MW>350, Rotors>7, XLOGP3>3.5	No; 2 violations: MW>350, Rotors>7	No; 3 violations: MW>350, Rotors>7,	No; 2 violations: Rotors>7, XLOGP3>3.5	No; 3 violations: MW>350, Rotors>7,	No; 3 violations: MW>350, Rotors>7,	No; 3 violations: MW>350, Rotors>7,	No; 3 violations: MW>350, Rotors>7,	No; 3 violations: MW>350, Rotors>7,	No; 3 violations: MW>350, Rotors>7,

			XLOGP3>3.5		XLOGP3>3.5	XLOGP3>3.5	XLOGP3>3.5	XLOGP3>3.5	XLOGP3>3.5	XLOGP3>3.5
**Synthetic accessibility**	4.82	3.88	3.36	4.00	5.22	5.22	5.22	8.02	7.93	7.81
	**Physicochemical Properties**									
	**1**	**2**	**3**	**4**	**5**	**6**	**7**	**11**	**12**	**13**
**Formula**	C24H46O8	C16H30O8	C25H50O2	C20H40O4	C30H60O4	C30H54O4	C30H54O5	C35H60O6	C35H58O6	C35H58O6
**Molecular weight**	462.62 g/mol	350.40 g/mol	382.66 g/mol	344.53 g/mol	484.80 g/mol	478.75 g/mol	494.75 g/mol	576.85 g/mol	574.83 g/mol	574.83 g/mol
**Number of heavy atoms**	32	24	27	24	34	34	35	41	41	41
**Number of aromatic heavy atoms**	0	0	0	0	0	0	0	0	0	0
**Fraction C*sp*^{3}**	0.92	0.88	0.96	0.95	0.97	0.73	0.73	0.94	0.89	0.89
**Number of rotatable bonds**	25	17	23	19	29	17	18	9	8	8
**Number of H-bond acceptors**	8	8	2	4	4	4	5	6	6	6
**Number of H-bond donors**	4	4	1	2	2	4	5	4	4	4
**Molar Refractivity**	124.70	86.24	124.06	101.86	149.93	149.15	150.31	165.61	165.14	165.14
**TPSA**	133.52 Å²	133.52 Å²	37.30 Å²	66.76 Å²	66.76 Å²	80.92 Å²	101.15 Å²	99.38 Å²	99.38 Å²	99.38 Å²
	**Lipophilicity**									
	**1**	**2**	**3**	**4**	**5**	**6**	**7**	**11**	**12**	**13**
**Log $P_{o/w}$ (iLOGP)**	4.02	3.04	5.68	4.91	6.75	6.75	6.75	5.25	5.51	5.41
**Log $P_{o/w}$ (XLOGP3)**	4.48	0.15	12.00	6.87	12.29	12.29	12.29	7.74	6.96	6.71
**Log $P_{o/w}$ (WLOGP)**	3.02	-0.10	9.06	4.75	8.66	6.94	5.91	5.85	5.63	5.63
**Log $P_{o/w}$ (MLOGP)**	1.75	-0.06	6.21	3.40	5.47	5.47	5.47	3.96	3.85	3.85
**Log $P_{o/w}$ (SILICOS-IT)**	5.47	2.01	9.21	5.86	10.25	10.25	10.25	5.02	4.84	4.84
**Consensus Log $P_{o/w}$**	3.75	1.01	8.43	5.16	8.68	8.68	8.68	5.56	5.36	5.29

Gastrointestinal (GI), blood-brain barrier (BBB), cytochrome P450 (CYP), P-glycoprotein (P-gp), pan assay interference structures (PAINS), topological polar surface area (TPSA).

Table S6: ADME Radar representations of compounds **1–7** and **11–13**

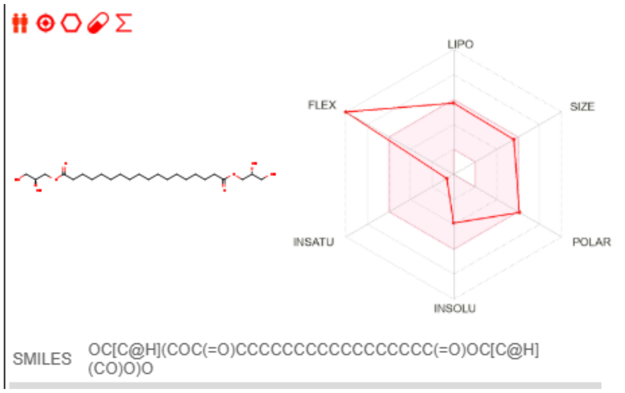

Compound **1**

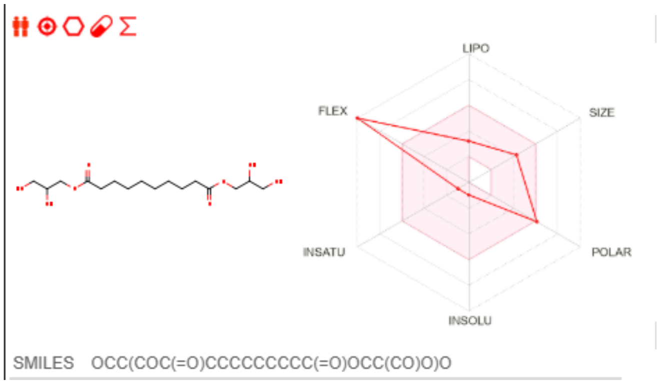

Compound **2**

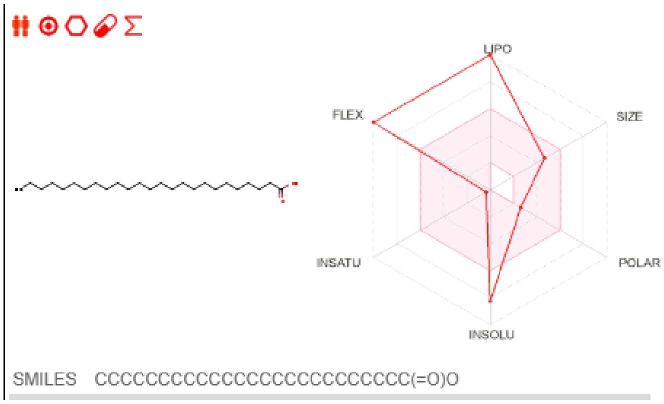

Compound **3**

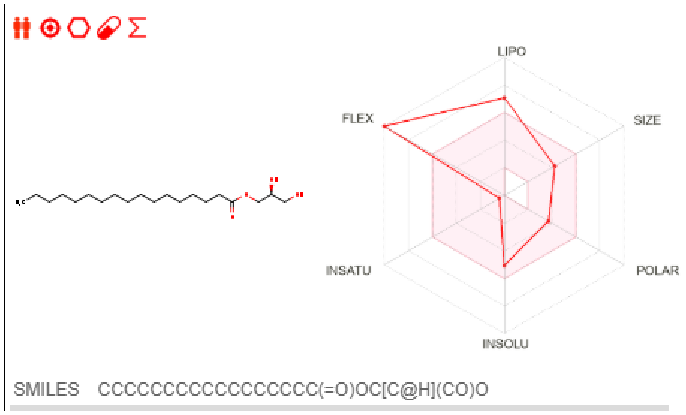

Compound **4**

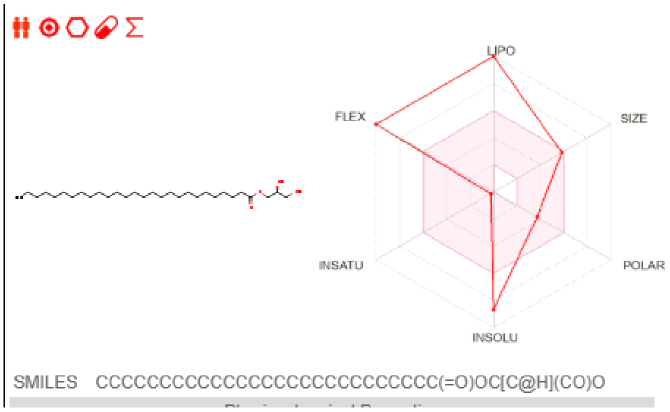

Compound **5**

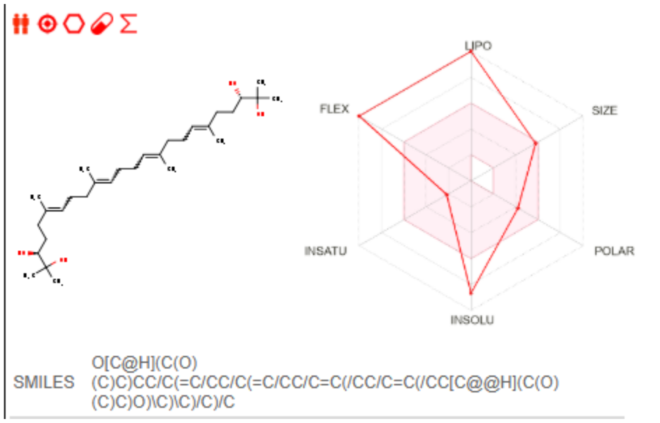

Compound **7**

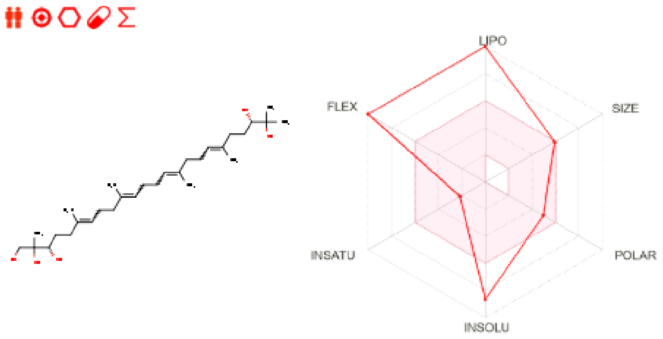

SMILES OCC([C@H](CC/C(=C/CC/C(=C/CC/C=C(/CC/C=C(/CC[C@@H](C(O)(C)C)O)\C)\C)/C)/C)O)(O)C

Compound **7**

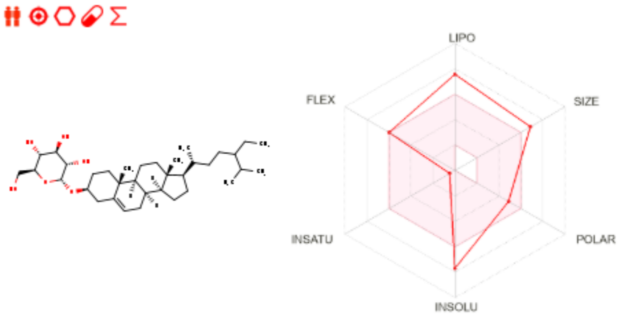

SMILES CCC(C(C)C)CC[C@H]([C@H]1CC[C@@H]2[C@]1(C)CC[C@H]1[C@@H]2CC=C2[C@]1(C)CC[C@@H](C2)O[C@@H]1O[C@@H](CO)[C@@H]([C@H]([C@@H]1O)O)O)C

Compound **11**

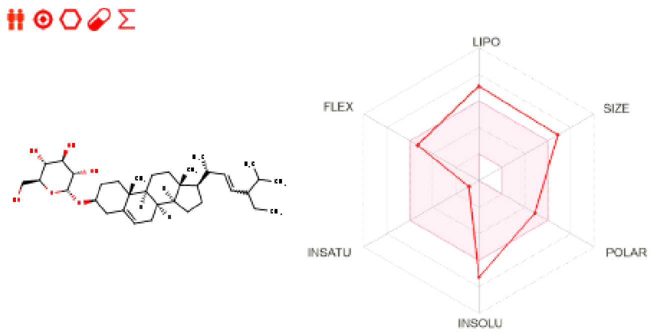

SMILES CCC(C(C)C)/C=C/[C@H]([C@H]1CC[C@@H]2[C@]1(C)CC[C@H]1[C@@H]2CC=C2[C@]1(C)CC[C@@H](C2)O[C@@H]1O[C@@H](CO)[C@@H]([C@H]([C@@H]1O)O)O)C

Compound **12**

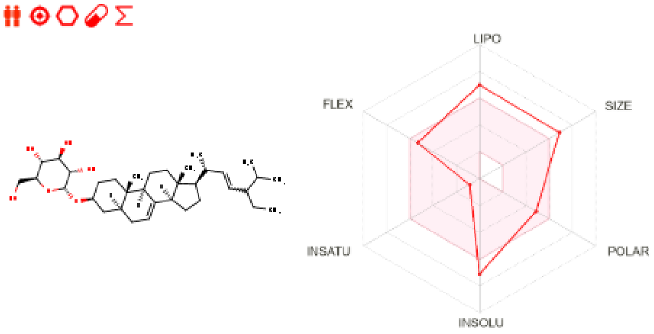

SMILES CCC(C(C)C)/C=C/[C@H]([C@H]1CC[C@@H]2[C@]1(C)CC[C@H]1C2=CC[C@@H]2[C@]1(C)CC[C@@H](C2)O[C@@H]1O[C@@H](CO)[C@@H]([C@H]([C@@H]1O)O)O)C

Compound **13**
